# Neuronal microexons modulate arousal via the cAMP-PKA-CREB pathway in zebrafish

**DOI:** 10.1126/sciadv.ady8291

**Published:** 2026-06-19

**Authors:** Tahnee Mackensen, Luis Pedro Iñiguez, Thomas Soares Mullen, Cristina Rodriguez-Marin, François Kroll, Giulia Zuccarini, Jordi Fernandez-Albert, Laia Sancho-Vila, Jon Permanyer, Isaac H. Bianco, Michael Orger, Jason Rihel, Manuel Irimia

**Affiliations:** ^1^ Universitat Pompeu Fabra, Barcelona, Spain.; ^2^ Centre for Genomic Regulation (CRG), Barcelona Institute of Science and Technology, Dr. Aiguader 88, Barcelona 08003, Spain.; ^3^ Biozentrum, University of Basel, Basel, Switzerland.; ^4^ Champalimaud Neuroscience Programme, Champalimaud Centre for the Unknown, Lisbon 1400-038, Portugal.; ^5^ Sorbonne Université, INSERM U968, CNRS UMR 7210, Institut de la Vision, Paris, France.; ^6^ Department of Cell and Developmental Biology, University College London, London, UK.; ^7^ Department of Neuroscience, Physiology & Pharmacology, University College London, London, UK.; ^8^ ICREA, Pg. Lluis Companys 23, Barcelona 08010, Spain.

## Abstract

Proper regulation of arousal maintains the balance of rest and activity and enables appropriate responses to stimuli; its disruption is a hallmark of many neurodevelopmental disorders. Although transcriptional mechanisms of arousal control are well defined, the contribution of posttranscriptional processes such as alternative splicing remains unclear. Here, we identify a critical role for the microexon splicing regulator *srrm3* in maintaining arousal homeostasis in zebrafish. *srrm3* mutants exhibit persistent hyperarousal characterized by sleep loss, sensory hypersensitivity, and elevated behavioral and neuronal activity. We identify the cyclic adenosine monophosphate (cAMP)–cAMP-dependent protein kinase (PKA)–cAMP response element–binding protein (CREB) signaling axis as a central driver of mutant hyperarousal. Specifically, pharmacological inhibition of cAMP signaling rescues mutant hyperactivity and associated transcriptional changes whereas wild-type cAMP activation phenocopies the mutant. Down-regulation of immediate early genes and reduced CREB phosphorylation further suggest adaptation to sustained neuronal activation. These findings establish *srrm3*-dependent microexon splicing as a key molecular layer of arousal regulation linking RNA-processing defects to neuromodulatory imbalance.

## Teaser

Loss of the conserved splicing factor for neuronal microexons disrupts neuromodulatory signaling and drives hyperarousal.

## INTRODUCTION

Animals continuously adapt their behavior to internal states and external demands, balancing rest and activity, stress, and recovery (*1*). This ability is orchestrated by the regulation of arousal, a process that governs the organism’s readiness to interact with the environment by modulating the activation of the central nervous system (*2*). Arousal includes a tonic component that sets the baseline level of responsiveness lower during sleep and higher during wakefulness and a phasic component that reflects externally triggered transient increases in alertness, for example, in response to sudden sound or light stimuli (*3*, *4*). Proper regulation of arousal ensures that neural and behavioral responses remain within an optimal range: Too little arousal leads to drowsiness or reduced responsiveness, whereas excessive arousal can result in heightened activity (or insomnia) and sensory hypersensitivity, states commonly associated with stress and neurodevelopmental disorders (*2*, *5*).

Arousal states are largely evolutionarily conserved across the animal kingdom, from jellyfish (*6*), fruit fly (*7*, *8*), and zebrafish (*9*–*11*) to humans (*12*), reflecting their fundamental importance to survival. In vertebrates, they are maintained by neuromodulatory systems, including noradrenergic, dopaminergic and cholinergic circuits (*7*, *8*), established early in development to dynamically adjust behavioral states (*13*, *14*). At the molecular level, these neuromodulators signal through diverse G protein–coupled receptors (GPCRs), many of which modulate intracellular cyclic adenosine monophosphate (cAMP) levels to engage downstream effectors. Canonically, elevated cAMP activates the cAMP-dependent protein kinase (PKA)–cAMP response element–binding protein (CREB) pathway, which translates transient changes in neuronal activity into long-lasting transcriptional responses (*15*, *16*). Activation of this pathway promotes the expression of activity-dependent genes, ultimately fine-tuning synaptic connections to maintain balanced neuronal excitability and arousal levels (*15*).

In contrast, far less is known about the posttranscriptional mechanisms that shape neuronal responsiveness and arousal control. Posttranscriptional control via alternative splicing is highest in the brain (*17*). Alternative splicing allows for the generation of multiple transcripts from a single gene, thus generating the protein diversity required for the precise temporal and spatial regulation of neural circuit formation (*18*). During neurodevelopment, the splicing factors SRRM3 and SRRM4 orchestrate a remarkable program of neuronal-specific microexon splicing (*19*, *20*). Microexons, defined here as 3– to 51–nucleotide (nt) exons, are highly conserved across vertebrates (*21*) and are widely dependent on the enhancer of microexons (eMIC) domain in the C-terminal region of SRRM3/4 (*19*, *22*–*24*). Their inclusion increases sharply during late neurogenesis and can subtly alter protein interactions, localization or efficiency, thereby fine-tuning processes such as axon guidance and synapse formation (*19*, *25*) with the potential to influence synaptic transmission and circuit formation (*26*, *27*).

Disruption of the neuronal microexon program alters neuronal activity and behavioral state regulation across species, suggesting a role in maintaining balanced arousal. In mice, *Srrm3/4* double mutants have severely reduced microexon inclusion and show impaired synapse formation and excitatory-inhibitory imbalance (*23*), whereas deletion of a single microexon in the synaptic organizer *Ptprd* is sufficient to disrupt sleep and increases locomotor activity, directly linking microexon loss to arousal-related behavior (*28*). Similarly, zebrafish *srrm3/4* double mutants display increased locomotor activity (*29*) and *Drosophila* lacking the eMIC domain of Srrm234 sleep less, particularly at night onset (*21*). Moreover, mouse mutants of *Srrm4* or of individual microexons such as *Cpeb4* further exhibit autism spectrum disorder (ASD)–like traits (*30*, *31*). Consistently, microexon misregulation has been observed in individuals with ASD (*19*, *30*, *32*) and schizophrenia (*33*), both disorders associated with sensory hypersensitivity and disturbed arousal regulation, including abnormal sleep-wake cycles (*5*, *34*). Together, these observations point to a conserved role of SRRM3/4-dependent splicing in stabilizing neuronal excitability and behavioral state, yet the mechanisms by which microexon mis-splicing perturbs neuromodulatory signaling and arousal control remain poorly understood.

Addressing this gap of knowledge requires an integrated approach that links posttranscriptional regulation to neuronal activity and behavior. Zebrafish provide an ideal vertebrate model to investigate how loss of *srrm3*-dependent microexons affects arousal control as they exhibit a complex behavioral repertoire by 5 days postfertilization (dpf) and have conserved arousal circuits, including the dopaminergic and noradrenergic systems (*14*, *35*). At this stage, neurogenesis is still ongoing (*36*), and microexons continue to shape critical developmental processes (*16*). Using *srrm3*^∆eMIC^ mutants, which lack the eMIC domain required for microexon inclusion, we combined high-throughput behavioral profiling, whole-brain activity imaging, and transcriptomic and pharmacological assays to connect microexon mis-splicing to changes in neuronal activity and behavior. We find that loss of microexons leads to persistent hyperarousal, marked by increased behavioral and forebrain activity, altered catecholaminergic pathway function, and misregulation of cAMP-PKA-CREB signaling, accompanied by a likely compensatory up-regulation of microexon-harboring genes. These findings establish microexon splicing as a crucial molecular layer in maintaining arousal homeostasis and link developmental RNA processing to neuromodulatory control of brain state.

## RESULTS

### Hyperactivity, sleep loss, excess thigmotaxis, and sensory hypersensitivity reflect hyperarousal in *srrm3*^∆eMIC^ larvae

Abnormal increases or decreases in spontaneous locomotor activity, as well as alterations in sleep amount or rhythms, are hallmarks of arousal-system dysregulation (*5*, *14*, *34*). We thus set out to determine how misregulation of the microexon program as a whole affects zebrafish larva sleep-wake behavior*.* We performed high-throughput behavioral tracking of 5 to 8 dpf *srrm3*^crg3/crg3^ zebrafish larvae that lack the *srrm3* eMIC domain (*37*), which is necessary and sufficient for microexon inclusion (*22*, *24*). This line was used throughout the study, and the homozygous mutant is hereafter referred to as *srrm3*^∆eMIC^, whereas the heterozygous mutant is referred to as *srrm3*^+/∆eMIC^.

**Fig. 1.**
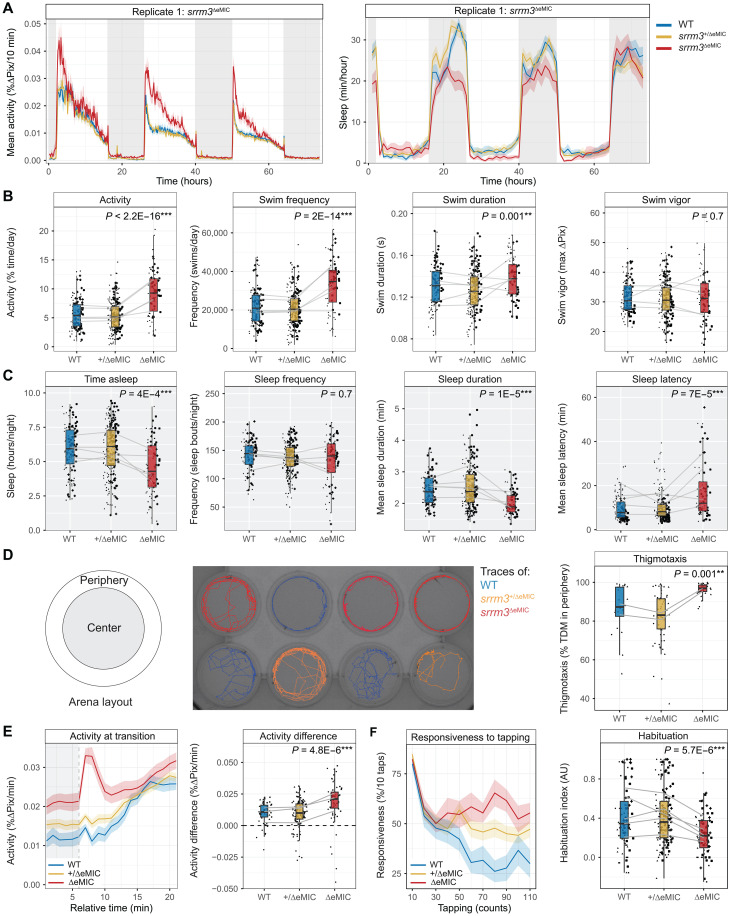
*srrm3*^∆eMIC^ larvae show hyperactivity, sleep loss, sensory hypersensitivity, and excess thigmotaxis. (**A**) Representative activity [%∆pixels (%∆Pix)/10 min] (left) and sleep (min/hour) (right) traces during 74 hours (5 to 8 dpf) on a 14-hour light/10-hour dark cycle (white/gray backgrounds). (**B** and **C**) Daytime activity (6 dpf) and nighttime sleep (5 to 6 dpf) parameters. For visualization, outliers were removed. *N* = 5 clutches with 13 to 57 larvae each per genotype. (**D**) Thigmotaxis at 6 dpf. Left: Arena layout and representative example trajectories (120 s of movement) around the 40th min of tracking. Right: Mean thigmotaxis (% TDM in periphery) during 1 hour of tracking (+10 ± 3% TDM in periphery, *P* = 0.001, LMM). *N* = 2 clutches with 10 to 43 larvae each per genotype. (**E**) Averaged (five transitions per larvae) light-on startle response at 6 dpf. Left: Means ± SEM traces (%∆Pix/min) from one representative clutch. Right: Activity (%∆Pix/min) difference (+0.010 ± 0.002%∆Pix, *P* = 4.8 × 10^−6^, LMM) 1 min poststimulus minus 1 min prestimulus. *N* = 3 clutches (dot shape), with 12 to 47 larvae each per genotype. (**F**) Habituation to mechanical taps at 6 dpf. Left: Representative trace (means ± SEM) of mean responsiveness (%) to 10 x 90-s ISI taps (1 to 10) and to 100 x 5-s ISI taps (11 to 100). Right: HI, where 1: full habituation and 0: none (HI: −0.19 ± 0.04 AU, *P* = 5.7 × 10^−6^, LMM). *N* = 5 clutches with 5 to 46 larvae each per genotype. (A to F) Statistics using the LMM with the likelihood ratio test, showing model coefficients ± SE and *P* values for WT versus *srrm3*^∆eMIC^ siblings. Each dot represents one larva, shaped by clutch. Lines connect genotype means across clutches. Detailed statistics and sample sizes are in data S1.

Measuring locomotor activity of *srrm3*^∆eMIC^ larvae across multiple days and nights, we observed a hyperactivity phenotype, with 61% more activity during the day and 66% more activity at night compared to wild-type (WT) siblings (Fig. 1, A and B, and fig. S1, A and B), which was also observed in a second founder line (*srrm3*^crg5/crg5^) (fig. S1, A and B). This hyperactivity, most prominent in the morning, was due to more frequent and longer swim periods but not due to increased swim vigor (active swim max ∆pixel), when compared to WT siblings (Fig. 1, A and B, and fig. S1B). *srrm3*^∆eMIC^ larvae also showed pronounced disturbances [sleep was defined as ≥1 min of inactivity based on an increased arousal threshold (*10*)], including a 47% delay in sleep onset [+5.7 ± 1 min, model coefficient ± SE from the linear mixed-effects model (LMM), *P* = 7 × 10^−5^] and shorter sleep episodes (−0.4 ± 0.1 min, coefficient ± SE from the LMM, *P* = 1 × 10^−5^), resulting in an average loss of up to 1.5 hours every 10 hours of sleep at night compared to WT larvae (Fig. 1, A and C, and fig. S1, A and B). For some parameters, these effects were more pronounced in *srrm3*^∆eMIC^*;srrm4*^−/−^ double mutants, whereas *srrm3*^+/∆eMIC^*;srrm4*^−/−^ larvae did not show signs of hyperactivity (fig. S1, A and B).

Although *srrm3*^∆eMIC^ larvae responded strongly to light transitions (Fig. 1A), they were reported to exhibit severe vision deficits (*37*). To test whether reduced vision can cause the hyperactivity phenotype, we compared locomotor activity in *vsx1*^−/−^;*vsx2*^−/−^ larvae, which have severe visual impairments, against *vsx2*^−/−^ siblings without visual abnormalities (*38*). Both genotypes showed high sleep levels, and *vsx1*^−/−^;*vsx2*^−/−^ larvae exhibited reduced nighttime activity relative to *vsx2*^−/−^ controls (fig. S1, A and B). This suggests that visual impairment alone does not necessarily result in hyperactivity or sleep loss, in line with multiple studies reporting hypoactivity in visually impaired and blind fish (*39*–*41*). This indicates that the sleep-wake phenotypes of the *srrm3*^∆eMIC^ larvae are unlikely caused by their vision deficits.

The disrupted sleep-wake patterns in *srrm3*^∆eMIC^ larvae indicate elevated baseline (tonic) arousal, which could be driven or amplified by the larva’s internal state, such as high stress levels (*14*). We thus quantified thigmotaxis (“wall hugging”), a behavioral indicator often associated with stress- or anxiety-like states in zebrafish larvae, although it can also occur in other contexts, including seizures (*42*–*44*). Thigmotaxis was high across genotypes; however, levels in the *srrm3*^∆eMIC^ were significantly above those of WT siblings [Fig. 1D; +10 ± 3% total distance moved (TDM) in periphery, coefficient ± SE from the LMM].

Having observed heightened tonic arousal, we next tested phasic, stimulus-induced arousal by exposing larvae to sudden dark/light transitions and repeated mechanical taps. In response to a light-on stimulus, *srrm3*^∆eMIC^ larvae displayed an exaggerated response, even considering their higher levels of baseline activity (Fig. 1E and fig. S1C), and their reduced visual function (*37*). Moreover, *srrm3*^∆eMIC^ larvae showed reduced habituation to repeated mechanical tapping stimuli [Fig. 1F and fig. S1D; −0.19 ± 0.04 habituation index (HI), coefficient ± SE from the LMM, *P* = 5.7 × 10^−6^], reflecting a persistently elevated readiness to respond in line with higher phasic arousal. Together, our findings suggest elevated baseline and phasic arousal in *srrm3*^∆eMIC^ larvae, exemplified by hyperactivity, insomnia, excessive thigmotaxis, and exaggerated stimulus-driven responses.

### Movement repertoire and kinematics reveal a shift toward stress-associated and seizure-like swim patterns in *srrm3*^∆eMIC^ larvae

**Fig. 2.**
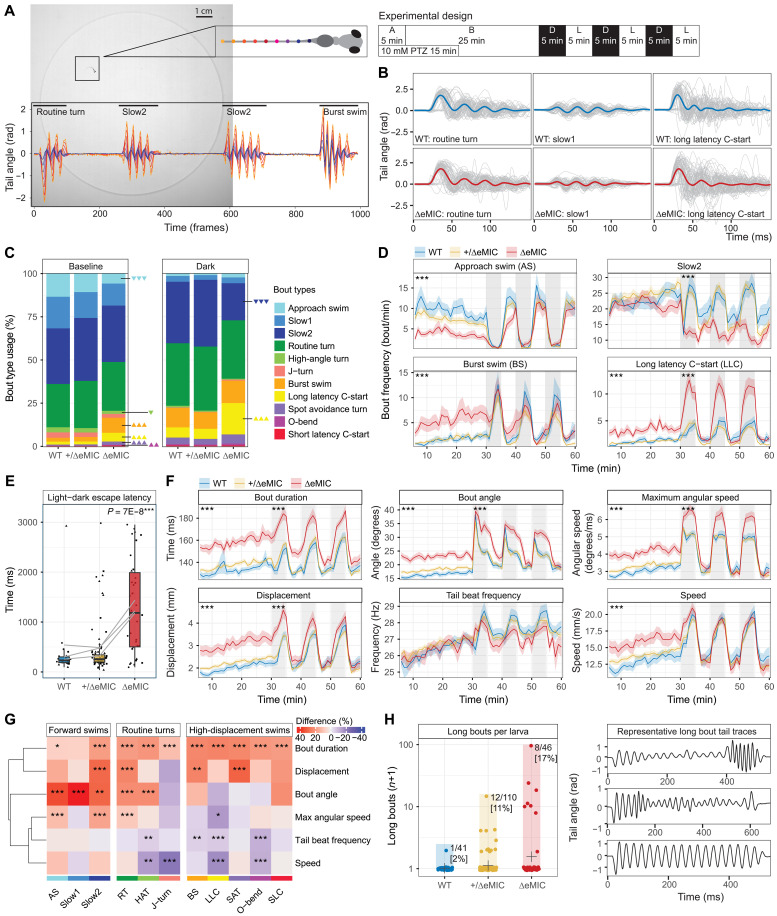
Bout usage and kinematics reflects heightened arousal in *srrm3*^∆eMIC^ larvae. (**A**) Left: Circular arena and tail-tracking scheme (nine segments, 300-μm spacing). Example WT bout sequence. Right: Experimental design: 5-min acclimation (A), 25-min baseline (B) (four clutches), then alternating 5-min dark/light (D/L) periods (three clutches) or 15-min 10 mM PTZ exposure (two clutches). (**B**) Representative tail traces (segment 7) for three bout types; 70 randomly sampled traces per type (gray); bold shows the mean. (**C**) Bout type usage (%). Triangles indicate the direction and significance of genotype differences (up: higher in *srrm3*^∆eMIC^; down: lower in *srrm3*^∆eMIC^). Statistics on raw counts using a negative binomial GLMM. Raw distributions in fig. S2D. (**D**) Mean bout frequency (bout/min) over time (means ± SEM). Significance marked for baseline and dark. (**E**) Mean latency to first escape after lights off (+235 ± 71%, coefficient ± SE from the LMM, *P* = 7 × 10^−8^). Dots represent larvae shaped by clutch. (**F**) Bout kinematics over time (means ± SEM), averaged over 1-min intervals. Significance marked for baseline and dark. (**G**) Percentage difference in bout kinematic means within each bout type (WT versus *srrm3*^∆eMIC^). Red: higher in ∆eMIC; blue: lower in ∆eMIC. *N* = 4 clutches with 3 to 33 larvae per genotype and bout type. (**H**) Long bouts (>500 ms). Left: Long bout count (log-scaled; +1 pseudocount); numbers indicate larvae (%) performing ≥1 long bout; cross: genotype mean. Right: Representative traces. *N* = 4 clutches with 7 to 34 larvae each per genotype. [(E) to (G)] Statistics compare WT and *srrm3*^∆eMIC^ siblings using Satterthwaite-approximated *t* tests on the LMM [log-transformed values for E; untransformed for (F) and (G)]. [(C) to (F)] *N* = 3 to 4 clutches with 7 to 33 larvae each per genotype. [(C), (D), (F), and (G)] Bonferroni correction for multiple testing. [(C) to (H)] Statistics and sample sizes are in data S2.

Although activity-based analyses indicate a hyperaroused state in *srrm3*^∆eMIC^ larvae, increased locomotion could, in principle, arise from motor impairments. To address this, we examined their movement repertoire and bout kinematics. Larval zebrafish locomotion consists of discrete bouts of tail movements that fall into a set of kinematically defined categories (*35*). Using high-speed videography (700 Hz) of freely swimming larvae under constant illumination (baseline) and, for some cohorts, during light-dark transitions (Fig. 2A), we identified 11 of the 13 canonical bout types based on 73 kinematic parameters each (*35*). The two absent categories, both capture swims, are predominantly executed in the presence of prey (*35*). The remaining bout types grouped into three categories: (i) low-displacement forward swims [approach swims (AS), Slow 1, and Slow 2]; (ii) low-displacement reorienting swims [routine turns (RTs), J-turns, and high-angle turns (HATs)]; and (iii) high-displacement swims [burst swims (BS), spot avoidance turns (SATs), O-bends, short-latency C-starts (SLCs), and long-latency C-starts (LLCs)] (*45*) (Fig. 2, A and B, and fig. S2A).

All 11 bout types were observed in both WT and *srrm3*^∆eMIC^ larvae, albeit at different frequencies (Fig. 2C). Within each bout category, the mean and distribution of bouts in a principal component (PC) space of kinematic features (*35*) were highly similar between genotypes (fig. S2, B and C), in stark contrast to larvae treated with the seizure-inducing compound pentylenetetrazol (PTZ) (fig. S2C). This indicates that the *srrm3*^∆eMIC^ phenotype does not reflect a gross motor deficit.

We therefore asked whether the difference lies instead in bout selection, known to reflect larva internal state (*46*, *47*). Under baseline conditions, WT larvae rarely use high-displacement swim types, which are typically recruited in response to external stressors such as sudden changes in illumination (Fig. 2, C and D). In contrast, *srrm3*^∆eMIC^ siblings used high-displacement bouts significantly more frequently, even at baseline (Fig. 2, C and D, and fig. S2, D and E). When exposed to sudden darkness, an acute stressor, both genotypes increased their usage of high-displacement swims, but *srrm3*^∆eMIC^ larvae continued to use them at significantly higher rates than WT [Fig. 2, C and D, and fig. S2D; LLC: +161 ± 51%, percentage ± percentage SE from transformed generalized LMM (GLMM) coefficients, *P*.adj = 8.7 × 10^−6^]. Notably, this occurred despite the significantly increased escape latency of *srrm3*^∆eMIC^ to the dark stimulus (Fig. 2E; latency: +235 ± 71%, percentage ± percentage SE from transformed LMM coefficients, *P* = 7.1 × 10^−8^), indicating heightened usage of stress-associated escape swims despite reduced visual responsiveness. Together, we observe heightened baseline and phasic arousal in the *srrm3*^∆eMIC^ also in the fine structure of single bout selection.

Building on the elevated use of stress-associated swim types (Fig. 2C) and pronounced circling along arena walls (Fig. 1D), previously linked to seizure susceptibility in zebrafish larvae (*48*), we next asked whether a subset of *srrm3*^∆eMIC^ larva bouts exhibits seizure-like kinematics. We examined six kinematic parameters previously associated with seizure-like swimming (*42*, *48*) (Fig. 2F). All parameters except tail-beat frequency were increased in *srrm3*^∆eMIC^ larvae compared to WT siblings across the experiment, with bout duration being particularly elevated across all bout types (Fig. 2, F and G, and fig. S2F). Moreover, abnormally long bouts (>500 ms) occurred in a significantly higher proportion of *srrm3*^∆eMIC^ (17%; 8/46 larvae) than WT (2%; 1/41 larvae) (Fig. 2H; *P* = 0.03, two-sided Fisher’s exact test). Among larvae exhibiting these events, *srrm3*^∆eMIC^ individuals executed unusually long bouts at markedly higher frequency (Fig. 2H; ∼21-fold increase, *P* = 7.1 × 10^−8^, two-sided Fisher’s exact test). These prolonged bouts also displayed significantly larger bout angles, higher maximum angular speeds, and greater displacement, swim speed, and tail-beat frequency compared to other *srrm3*^∆eMIC^ bouts (*P* ≤ 0.001, permutation tests), consistent with seizure-like kinematics (*42*, *48*). Together, although *srrm3*^∆eMIC^ larvae retain a normal bout repertoire, they show a marked shift toward stress-associated swims and an increased occurrence of rare, prolonged bouts with seizure-like kinematics, further supporting a state of heightened arousal.

### *srrm3*^∆eMIC^ larvae exhibit neuronal activity signatures of hyperarousal

**Fig. 3.**
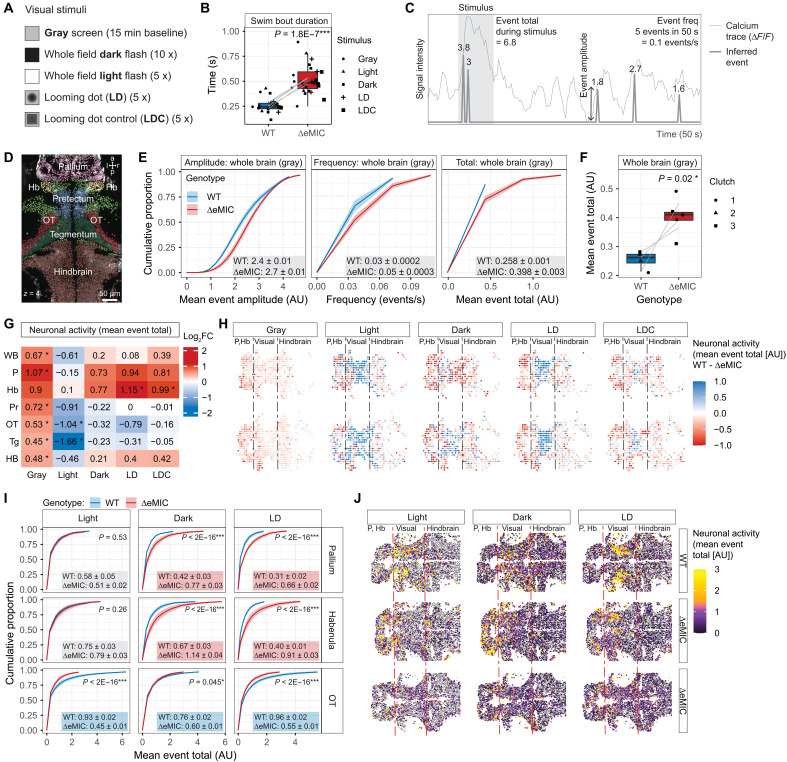
*srrm3*^∆eMIC^ show elevated neuronal activity and altered responses to visual stimuli. (**A**) Protocol: 15-min baseline and then pseudorandom visual stimuli (∼3 to 5 s, 5 to 10 repeats). (**B**) Swim duration (larva median). Dots: larvae; shapes: stimuli; lines connect stimulus means. Statistics: GLM controlling for stimulus and individual. (**C**) Example calcium trace (∆*F*/*F*, thin) with inferred spike events (bold); event total is the normalized mean sum of event amplitudes across stimulus windows (gray). (**D**) Example field of view with ROIs colored by the ZBB anatomical region. (**E**) Whole-brain ECDFs for event amplitude, frequency, and total (baseline). Statistics by the Wilcoxon test. *n* = 36,951 WT and *srrm3*^∆eMIC^ 38,940 neurons. (**F**) Whole-brain event total at baseline (dots: larva means; lines: connect clutch means). Statistics by the Wilcoxon test. (**G**) Region-wise log_2_ fold changes (FC) of event total (∆eMIC versus WT). Statistics by the Wilcoxon test. (**H**) ZBB-registered anatomy maps (rows: *z*-planes) showing spatial differences in event total (10-μm bins; truncated to ±1 for visualization). (**I**) ECDFs of event totals across three stimuli and regions. Statistics by the Wilcoxon test. *n* = 1407 to 9662 neurons per condition and region. Boxed values colored by the direction of change. [(E) and (I)] Lines: means ± SEM cut at *y* = 0.97. Boxed values show means ± SEM across neurons. (**J**) Anatomy plots (single *z*-plane) from one clutch with neurons (dots) colored by mean event total (values > 3 set to 3 for visualization). [(G) to (I)] Red: ∆eMIC higher; blue: WT higher; gray: not significant (n.s.). [(H) and (J)] Dashed lines as anatomical guidelines for P and Hb; visually responsive (Pr, Tg, and OT); hindbrain. [(B) and (E) to (J)] Sample sizes: *N* = 3 clutches with four to five larvae per genotype. Abbreviations: a, anterior; p, posterior; r, rightward; l, leftward; WB, whole brain; P, pallium; Hb, habenula; Pr, pretectum; OT, optic tectum; Tg, tegmentum; HB, hindbrain.

Changes in arousal are caused by differences in central nervous system activation, with neural circuits showing state-dependent shifts in firing across sleep-wake cycles and other arousal transitions (*47*, *49*). Hence, we next asked whether the hyperaroused behavioral state of *srrm3*^∆eMIC^ larvae is associated with altered patterns of brain activity. For this purpose, we assessed neuronal activity across brain regions using two-photon (2P) calcium imaging under baseline (no stimulus, gray screen) and during visual stimuli [light, dark, looming dot (LD), and looming dot control (LDC)] (Fig. 3A). Notably, *srrm3*^∆eMIC^ larvae remained behaviorally hyperactive even with their heads restrained under the microscope, exhibiting longer bouts across stimuli [Fig. 3B; +0.26 ± 0.04 s, coefficient ± SE, generalized linear model (GLM), *P* = 1.8 × 10^−7^] and increased bout frequency (fig. S3A; +1.3 ± 0.6 bouts per epoch, coefficient ± SE from the GLM, *P* = 0.02) compared to WT siblings.

For neuronal activity imaging, we generated an *srrm3*^∆eMIC^ line expressing a nuclear GCaMP6s calcium indicator pan-neuronally (*50*). Neuronal activity was estimated from deconvolved calcium data (*51*), using event frequency, amplitude, and event total (summed event amplitudes over the stimulus window) (Fig. 3C). We measured from neurons of the anterior dorsal brain of sibling larvae (Fig. 3D and fig. S3B) and found that all three parameters were significantly elevated in *srrm3*^∆eMIC^ neurons at baseline (Fig. 3E), with event frequency remaining increased even when excluding movement periods (fig. S3C). Similar differences were observed when activity was measured at the level of individual larvae (Fig. 3, F and G, and fig. S3, D and E).

Event amplitudes in *srrm3*^∆eMIC^ larvae remained elevated also across stimulus conditions (fig. S3F), despite comparable GCaMP6s levels between genotypes (fig. S3G), possibly reflecting more rapid burst firing and summation of calcium transients in *srrm3*^∆eMIC^ neurons (*52*). In contrast, visually responsive brain regions (*53*, *54*) generally showed reduced visually evoked activity (Fig. 3, G to J, and fig. S3H), consistent with the mutants’ impaired visual function (*37*). This effect was most prominent during the whole-field light flashes, where *srrm3*^∆eMIC^ optic tectum activity was ∼50% below WT levels (Fig. 3, I and J; *P* < 2.2 × 10^−16^, Wilcoxon test). Despite a reduced response in the optic tectum, *srrm3*^∆eMIC^ forebrain and hindbrain neuronal activity remained elevated during stress-associated stimuli of darkness and LD (Fig. 3, G to J, and fig. S3I). Thus, paralleling the behavioral data, we found that *srrm3*^∆eMIC^ larvae exhibit heightened neuronal activity both at baseline and, with the exception of visually responsive brain regions, during arousal-inducing stimuli, consistent with a hyperaroused brain state.

### Conserved neuronal microexons are mis-spliced in *srrm3*^∆eMIC^ neurons

**Fig. 4.**
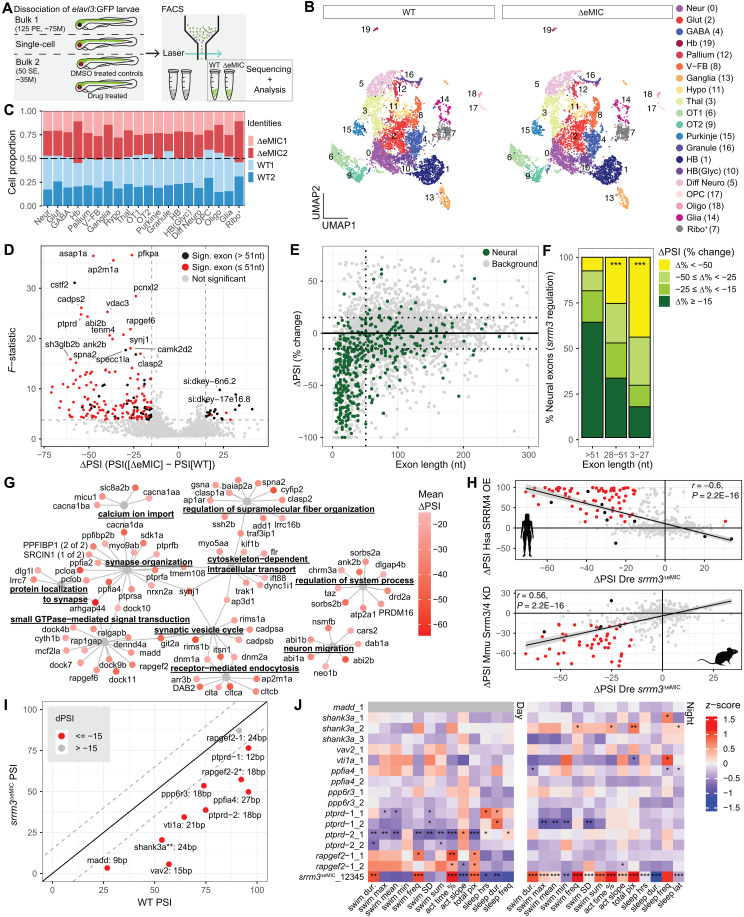
Widespread microexon skipping in *srrm3*^∆eMIC^ but unaltered neural cell type composition. (**A**) Experimental design: bulk and single-cell RNA-seq comparing WT with *srrm3*^∆eMIC^ siblings. Bulk 1 for splicing analysis; bulk 2 for DGE. PE, paired-end; SE, single-end. (**B**) Uniform Manifold Approximation and Projection (UMAP) of 20 single-cell clusters (PC1 to PC16), split by genotype. (**C**) Genotype contributions per cluster (n.s.; *t* test). [(B) and (C)] Cluster names in Materials and Methods. (**D**) Differential splicing of microexons (≤51 nt) and longer exons (>51 nt). Red and black points denote significantly mis-spliced exons (|ΔPSI| > 15, *F*-statistic > 3.8; dashed lines). (**E**) ΔPSI (%) versus exon length. Green: neuron-specific exons. Dashed lines indicate |∆PSI (%)| = 15 and exon length = 51 nt. (**F**) Distribution of ΔPSI (%) across exon length bins. Comparing highly skipped (∆% < −50) versus others: longer exons (>51 nt) show less skipping than shorter exons (3 to 27 nt, OR = 9.7, *P*.adj = 1.6 × 10^−15^; 28 to 51 nt, OR = 4.2, *P*.adj = 5 × 10^−4^). Two-sided Fisher’s exact test, Bonferroni-adjusted. (**G**) Subset of GO biological processes (*P*.adj < 0.05; excluding terms with ≥5 overlapping genes) enriched among genes with *srrm3*-regulated exons (∆PSI ≤ −15). Nodes: genes colored by mean ΔPSI. (**H**) Correlations of *srrm3*^∆eMIC^ ∆PSI with SRRM4 overexpression ∆PSI in human cells or with *Srrm3/4* knockdown ∆PSI in mouse (Pearson). Exons with |ΔPSI| ≥ 15 highlighted (red: ≤51 nt; black: >51 nt). Lines show linear regression fits ± SE. (**I**) Microexon PSI in WT versus *srrm3*^∆eMIC^ corresponding to available microexon mutant lines used in (J). Dashed lines mark |ΔPSI| = 15. *No behavioral data. **Low coverage. (**J**) Behavioral fingerprints of microexon mutants compared to WT siblings (*z*-scores). *srrm3*^∆eMIC^ shown for comparison. Gray: missing data. Biological replicates (1 to 5) or averaged replicates (12345) indicated. Likelihood ratio test statistics on the LMM and full parameter names are in data S1.

Having established elevated behavioral and neuronal activity in the *srrm3*^∆eMIC^, and considering that the only known molecular function of *srrm3* thus far is to promote microexon inclusion (*19*, *22*–*24*), we next examined the primary consequence of *srrm3* loss—disrupted neuronal microexon inclusion—and its secondary transcriptomic effects to identify molecular changes that could underlie the altered arousal state. We profiled neuronal transcriptomes by performing bulk RNA sequencing (RNA-seq) on fluorescence-activated cell sorting (FACS)–sorted *elavl3*:GFP^+^ cells, from 5 dpf WT and *srrm3*^∆eMIC^ mutant siblings (Fig. 4A), generating high-depth 125-nt paired-end reads, suitable for both alternative splicing and differential gene expression analysis (Fig. 4A). Mutants showed a marked down-regulation of vision-related genes (including *opn1sw1*, *pde6ha*, *gnat2*, and *grk7a*), consistent with their known photoreceptor degeneration (*37*), and raising the possibility that retinal signals might mask gene expression changes driven by other neurons (fig. S4A and data S6). To address this, we enucleated zebrafish larvae (6 dpf) before FACS sorting and generated both a single-cell RNA-seq dataset and a complementary second, shallower bulk RNA-seq dataset of 50-nt single-end reads (Fig. 4A) used for downstream gene expression and pharmacological comparisons.

Before exploring specific transcriptomic changes, we first asked whether loss of *srrm3* alters neuronal cell type composition as mouse mutants of its paralog *Srrm4* show shifts in neuronal subtype abundance (*25*). From the single-cell RNA-seq data, we identified 20 cell clusters corresponding to distinct cell populations across anatomical regions, differentiation stages, and major cell types (Fig. 4B; fig. S4, B and C; and data S6). *srrm3* was expressed across WT neuronal clusters (fig. S4D), consistent with brain-wide *srrm3* expression as detected by hybridization chain reaction (HCR) (fig. S4E). Comparing the proportions of *srrm3*^∆eMIC^ and WT cells across clusters revealed no significant differences (Fig. 4C; *P*.adj > 0.05 across clusters, *t* test), indicating no detectable shift in cell type composition or loss of specific populations. This agrees with an absence of gross anatomical abnormalities in *srrm3*^∆eMIC^ brains at 6 dpf (except for reduced eye size) (fig. S4F), suggesting that the primary consequences of *srrm3* loss lie at the level of neuronal physiology and signaling rather than cell type composition.

Next, we focused on the detection of mis-spliced microexons in the deep bulk RNA-seq dataset. Among alternative exons with sufficient read coverage (3752; see Materials and Methods), 5% (199, in 183 genes) were significantly differentially spliced [absolute ∆ percentage spliced in (|∆PSI|) ≥ 15, *F*-statistic > 3.8] in *srrm3*^∆eMIC^ neurons (data S6). As expected, most of these exons (89%) were skipped in the mutants, with 75% of them classified as microexons (≤51 nt) (Fig. 4D). Notably, neuron-specific microexons were also more strongly affected as 44% of 3- to 27-nt exons showed >50% reduced inclusion compared to 8% for long exons (>51 nt), a 5.5-fold increase (Fig. 4, E and F; *P*.adj = 1.6 × 10^−15^, two-sided Fisher’s exact test). In line with previous studies (*19*, *29*), *srrm3*-dependent exons were enriched in genes involved in a range of biological processes, including “receptor-mediated endocytosis” and “synapse organization” (Fig. 4G). Moreover, they showed a significant overlap with orthologous exons misregulated in *Srrm3/4*-knockdown mouse cell lines (*22*) (Fig. 4H; *r* = 0.6, *P* < 2.2 × 10^−16^, Pearson correlation) and those affected by human *SRRM4* overexpression (*24*) (Fig. 4H; *r* = −0.6, *P* < 2.2 × 10^−16^, Pearson correlation), consistent with the high evolutionary conservation of the *srrm3/4* microexon program across vertebrates (*19*, *24*).

To probe the potential contribution of individual microexons to the *srrm3*^∆eMIC^ hyperarousal phenotype, we pursued two complementary strategies: (i) leveraging the *srrm3*^+/∆eMIC^, whose behavior is largely indistinguishable from WT, to identify microexons whose mis-splicing may be phenotypically silent and (ii) analyzing sleep-wake signatures in single microexon zebrafish mutants. Similar to the behavioral data, bulk RNA-seq revealed only minimal overlap in differentially expressed genes (DEGs) between *srrm3*^*+/*∆eMIC^ and *srrm3*^∆eMIC^ larvae, including reduced *srrm3* expression itself (fig. S5, A to C, and data S6). Similarly, only six exons were significantly (*F*-statistic > 3.8, |∆PSI| > 15) mis-spliced in the *srrm3*^*+/*∆eMIC^ larvae (fig. S5D and data S6), and only one zebrafish-specific microexon, in *shroom2a*, was significantly more skipped in both mutant genotypes (DreEX0007899, +/∆eMIC: ∆PSI = −40; ∆eMIC: ∆PSI = −46). Thus, the heterozygous data did not reveal a subgroup of microexons that could be excluded as contributors to the hyperarousal phenotype. We next examined previously generated microexon mutants of *shank3*, *vti1a*, *vav2*, and *madd* (*29*), as well as published behavioral data for *ppfia4*, *ppp6r3*, *rapgef2*, and *ptprd* microexon mutants (*55*) (Fig. 4, I and J). Whereas most microexon mutants displayed mild or no sleep-wake phenotypes, deletion of a microexon in *rapgef2* recapitulated multiple aspects of the *srrm3*^∆eMIC^ daytime hyperactivity, although not the nighttime changes. In contrast, *ptprd* microexon mutants slept more and were less active, thus showing phenotypes opposite to those of the *srrm3*^∆eMIC^ larvae (Fig. 4J). Together, although individual microexon deletions can affect sleep and activity, the *srrm3*^∆eMIC^ hyperarousal phenotype unlikely arises from loss of a single microexon but instead from the combined misregulation of dozens of neuronal microexons, none of which are significantly altered in the *srrm3*^*+/*∆eMIC^.

### Up-regulation of microexon-containing genes suggests a compensatory response

**Fig. 5.**
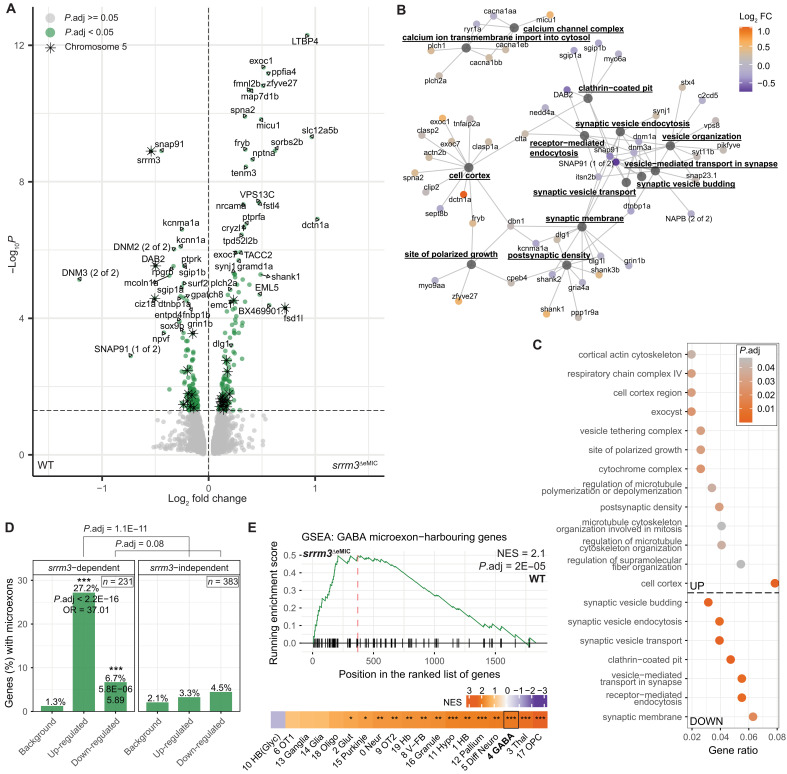
Up-regulated genes in *srrm3*^∆eMIC^ disproportionately harbor mis-spliced microexons. (**A**) Differential gene expression between *srrm3*^∆eMIC^ and WT neurons (6 dpf, enucleated). DEGs (*P*.adj < 0.05) are shown in green; up-regulated genes in the *srrm3*^∆eMIC^ appear to the right, and down-regulated genes appear to the left. Stars mark DEGs on the same chromosome as *srrm3* (chromosome 5). *N* = 8 biological replicates per genotype (10 to 24 larvae each). (**B**) Network of enriched GO terms (*P*.adj < 0.05) for DEGs between WT and *srrm3*^∆eMIC^, colored by log_2_FC (orange: up-regulated; purple: down-regulated). (**C**) Enrichment of unique GO terms for up-regulated (top) and down-regulated (bottom) genes ranked by gene ratio and colored by *P*.adj (*P*.adj < 0.05; set size: 10 to 200). (**D**) Enrichment of *srrm3*-dependent microexon genes (∆PSI < −15, ≤51 nt) among DEGs relative to background (231/18,341 expressed genes). Up-regulated genes showed stronger enrichment (50/184) than down-regulated genes (12/179). *srrm3*-independent microexon genes showed no enrichment. Statistics by two-sided Fisher’s exact tests with Bonferroni correction. Enrichment was significantly greater for *srrm3*-dependent than *srrm3*-independent genes (up-regulated: *Z* = 6.90, *P*.adj = 1.1 × 10^−11^; down-regulated: *Z* = 2.04, *P*.adj = 0.08; *z* test for OR differences). (**E**) GSEA of microexon-harboring genes (∆PSI < −15, length ≤51 nt) on log_2_FC between WT and *srrm3*^∆eMIC^ across single-cell clusters. *N* = 2 biological replicates per genotype with 13 to 21 larvae each. Example enrichment curve shown for GABAergic neurons (NES = 2.1, *P*.adj = 2 × 10^−5^). *P* values FDR corrected for multiple testing across clusters. Full cluster names are in Materials and Methods.

We next examined changes in gene expression, which are likely secondary to microexon mis-splicing (*37*) and can reveal which cellular pathways are disrupted in *srrm3*^∆eMIC^ larvae. Differential expression analysis from RNA-seq of enucleated WT and *srrm3*^∆eMIC^ siblings identified 363 DEGs (Fig. 5A and data S6; *P*.adj < 0.05). Down-regulated genes were enriched for synaptic vesicle and endocytosis-related Gene Ontology (GO) terms, whereas up-regulated genes were linked to cytoskeletal processes; both sets also contained calcium-channel genes (Fig. 5, B and C, and data S6). Notably, several DEGs harbored conserved microexons with previously characterized functions (Fig. 5, A and B), such as *cpeb4* (DreEX0023919: ∆PSI = −27) in neurotransmission (*31*) or *micu1* (DreEX0046514: ∆PSI = −42; DreEX6103867: ∆PSI = −17) in the regulation of mitochondrial calcium levels (*56*). Consistent, with *micu1* microexon skipping impairing mitochondrial calcium handling, pharmacological inhibition of the Micu1-gated mitochondrial calcium uniporter resulted in reduced survival of *srrm3*^∆eMIC^ larvae compared to WT siblings (fig. S6A).

These patterns thus point to a potential compensation for reduced protein function upon microexon skipping by up-regulating the host gene expression levels. To test for such a compensatory pattern more globally, we performed a gene set enrichment analysis (GSEA) using genes with *srrm3*-dependent microexons (∆PSI < −15; length ≤51 nt) as a query set. These genes were significantly and strongly enriched among up-regulated transcripts [fig. S6B; normalized enrichment score (NES) = 2.6, *P*.adj = 1 × 10^−10^]. Fifty of 184 (27%) up-regulated and 12 of 179 (7%) down-regulated genes harbored *srrm3*-dependent microexons, compared to 1% among all expressed genes in the background set [Fig. 5D; up: odds ratio (OR) = 37, *P* < 2.2 × 10^−16^; down: OR = 6; *P* = 2.9 × 10^−6^ two-sided Fisher’s exact test]. Single-cell RNA-seq data supported these findings: *srrm3*-dependent microexon-harboring genes were significantly enriched among up-regulated genes in 14 of 19 clusters, including glutamatergic (excitatory) and GABAergic (inhibitory) neurons (Fig. 5E, fig. S6C, and data S6). Notably, the enrichment among DEGs was specific to *srrm3*-dependent microexon-harboring genes and was not observed for *srrm3*-independent microexon-harboring genes (Fig. 5D). One possible explanation for the down-regulated subset is nonsense-mediated decay (NMD) because skipping of certain microexons can introduce frameshifts (*23*). However, among down-regulated microexon-harboring genes, only 1 of 11 exons were predicted to disrupt the reading frame upon skipping, arguing against NMD as a major driver of expression changes (fig. S6D). Given that the vast majority of *srrm3*-dependent exons—and all of the differentially included microexons in up-regulated genes—are predicted to preserve the protein reading frame (fig. S6D), their host-gene up-regulation is consistent with a compensatory response to reduced protein function upon microexon skipping in *srrm3*^∆eMIC^ zebrafish neurons. Supporting this, paralogs of mis-spliced genes were often coregulated, particularly if the paralog pair had a high sequence similarity (*P* = 1 × 10^−4^, permutation test) (fig. S6E).

Together, we observed broad transcriptional changes in *srrm3*^∆eMIC^ neurons, alongside a selective up-regulation of genes harboring *srrm3*-dependent microexons. This pattern is consistent with adaptive compensation following microexon skipping, as observed in single-microexon mutants (*29*), although it remains unclear to what extent such up-regulation restores altered protein function. Multiple disrupted pathways emerge as plausible links between microexon loss and altered neuronal physiology, including calcium handling, enriched for mis-spliced microexons, and synaptic vesicle endocytosis, enriched among DEGs.

### Behavioral pharmacology reveals cAMP signaling as a central driver of *srrm3*^∆eMIC^ daytime hyperactivity

**Fig. 6.**
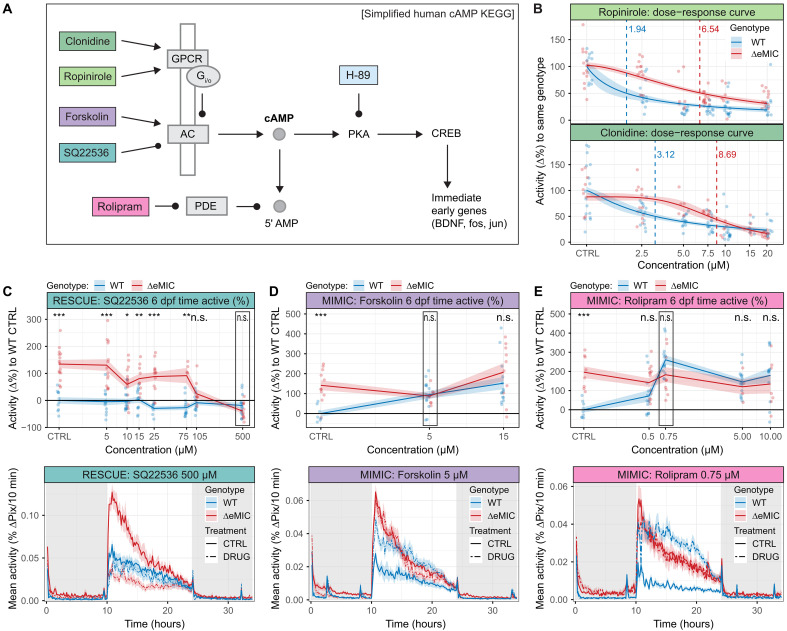
cAMP signaling is central to *srrm3*^∆eMIC^ daytime hyperactivity. Drug colors across the figure reflect the expected effects on cAMP: green: indirect reduction (clonidine and ropinirole); turquoise: direct reduction (SQ22563; *srrm3*^∆eMIC^ rescue); pink/purple: increase (forskolin and rolipram; *srrm3*^∆eMIC^ mimic) or the downstream effector PKA; light blue: reduced activity (H-89). (**A**) Simplified cAMP pathway [(Kyoto Encyclopedia of Genes and Genomes (KEGG)] highlighting drug targets. AC, adenylyl cyclase; GPCR, G protein–coupled receptor; PDE, phosphodiesterase. (**B**) Dose-response curves for ropinirole and clonidine. Points show % change in daytime activity (% time active) relative to same-genotype controls (CTRL) for each larva; curves (estimate ± SE) show 4PL fits (lower asymptote = 0). Concentrations on the log-scaled *x* axis. Dashed lines mark ED_50_ estimates ± SE: ropinirole WT = 1.9 ± 1.3, ∆eMIC = 6.5 ± 1.2 (*P* = 5.3 × 10^−5^); clonidine WT = 3.1 ± 1.3, ∆eMIC = 9.7 ± 1.2 (*P* = 1.1 × 10^−4^). Statistics: *t* test using compParm. *n* = 5 to 16 larvae per condition. (**C** to **E**) Top: Percentage change in daytime activity (% time active) of larvae (dots) relative to WT controls (black line) across concentrations of SQ22536 (rescue), forskolin (mimic), and rolipram (mimic). Lines show means ± SEM. Boxes indicate experiments shown below. Concentrations on the log-scaled *x* axis. Statistics by the Wilcoxon test. *n* = 5 to 15 (SQ22536), 7 to 15 (forskolin), 4 to 16 (rolipram) larvae per condition. Bottom: Mean activity traces (%∆Pix/10 min) for drug-treated (dashed) and control (solid) larvae recorded over 34 hours (5 to 7 dpf) under 14-hour light/10-hour dark (white and gray background, respectively). *n* = 8 to 15 larvae per condition.

Given that the disrupted splicing and gene-expression programs affect fundamental neuronal functions critical for internal state regulation (*57*) and that *srrm3*^∆eMIC^ larvae show marked hyperarousal, we next asked whether these molecular changes are associated with altered function of the canonical arousal regulators, dopamine and noradrenaline. In zebrafish, noradrenergic signaling promotes wakefulness (*49*) and dopaminergic activation drives locomotion (*58*). We therefore targeted these arousal-promoting pathways pharmacologically and monitored the effect on daytime hyperactivity (Fig. 6A).

Activation of the dopaminergic system with the D2-like receptor agonist ropinirole caused a dose-dependent reduction in activity in both genotypes, but *srrm3*^∆eMIC^ larvae were significantly less sensitive to the treatment (Fig. 6B and fig. S6A). Similarly, reducing noradrenergic tone with the α2-adrenergic receptor agonist clonidine induced a dose-dependent reduction in activity for both genotypes, but again with reduced sensitivity in *srrm3*^∆eMIC^ larvae (Fig. 6B and fig. S6A). These results indicate an altered responsiveness of both arousal pathways in the mutant but do not identify either dopamine or noradrenaline as the sole upstream driver of daytime hyperactivity.

Because both ropinirole (D2-like) and clonidine (α2-adrenergic) act on G_i/o_-coupled GPCRs that inhibit adenylyl cyclase to reduce cAMP synthesis (*59*, *60*), the similarly blunted response in *srrm3*^∆eMIC^ suggests a shared downstream pathway alteration (Fig. 6A). We thus hypothesized that elevated cAMP signaling might underlie the hyperactivity in *srrm3*^∆eMIC^ larvae. Consistently, reducing cAMP synthesis with the adenylyl cyclase inhibitor SQ22536 rescued *srrm3*^∆eMIC^ hyperactivity, normalizing activity to WT levels without noticeably affecting WT behavior (Fig. 6C and fig. S6A). In contrast, increasing cAMP levels, either by enhancing synthesis with the adenylyl cyclase activator forskolin or by decreasing its degradation with the phosphodiesterase inhibitor rolipram, did not further increase *srrm3*^∆eMIC^ activity but induced hyperactivity in WT larvae, remarkably mimicking the daytime activity levels observed in *srrm3*^∆eMIC^ (Fig. 6, D and E, and fig. S6A). These results strongly support elevated cAMP signaling as a central driver of the daytime hyperactivity phenotype in *srrm3*^∆eMIC^ larvae.

### Misregulation of the cAMP-PKA-CREB signaling pathway in *srrm3*^∆eMIC^ larvae

**Fig. 7.**
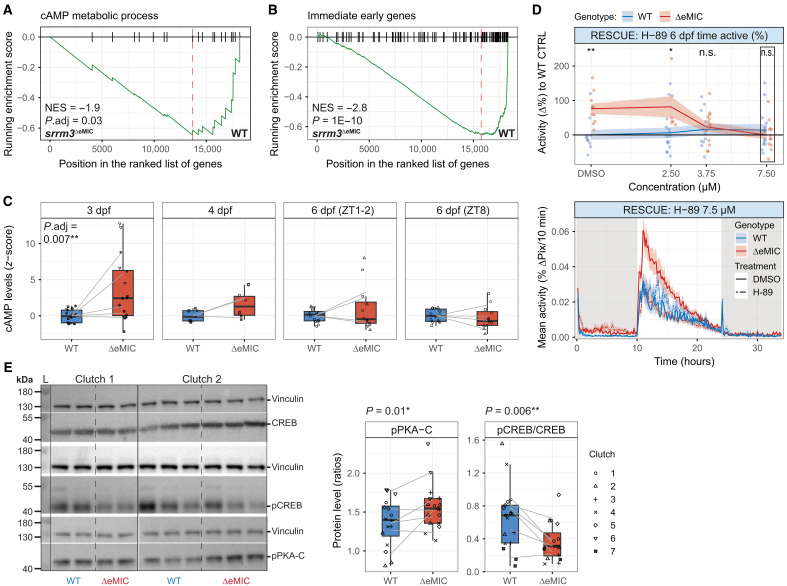
Misregulation of the cAMP-PKA-CREB pathway in *srrm3*^∆eMIC^ larvae. (**A** and **B**) GSEA on log_2_FC (*srrm3*^∆eMIC^ versus DMSO-treated WT) for (A) “cAMP metabolic process” and (B) mouse ortholog IEGs (*61*). (**C**) Whole-larvae cAMP levels at 3 dpf (ZT5 to ZT8), 4 dpf (ZT5 to ZT6.5), and 6 dpf (ZT1 to ZT2 or ZT8). Each point: one pooled sample (five to eight larvae), shaped by clutch. Lines connect genotype means across clutches. *N* = 3 to 5 clutches per time point with *n* = 2 to 4 samples per genotype and condition. (**D**) Top: Percentage change in daytime activity (% time active) relative to WT controls (black line) for H-89–treated larvae (dots). Lines show means ± SEM; the *x* axis is log-scaled. Box indicates the experiment shown below. Statistics by the Wilcoxon test. *n* = 6 to 15 larvae per condition. Bottom: Mean activity (%∆Pix/10 min) over 34 hours (5 to 7 dpf) for H-89–treated (dashed) and control (solid) larvae under 14-hour light/10-hour dark (white and gray background, respectively). *n* = 10 to 13 larvae per condition. (**E**) Western blots (two representative clutches) (left) and quantification of pPKA-C and pCREB/CREB ratios (each normalized to vinculin) across all clutches. Each point: lysate from five to eight larval heads (6 dpf). Lines connect genotype means across clutches. Statistics by the likelihood ratio test on the LMM. *N* = 6 clutches per protein of interest with *n* = 2 to 3 biological replicates per genotype and clutch. L, ladder.

To investigate molecular correlates of cAMP pharmacological manipulation, we first examined coordinated expression changes in cAMP-associated genes. GSEA revealed significant down-regulation of phosphodiesterases (enzymes degrading cAMP) and genes annotated with the GO term “cAMP metabolic process,” a set largely composed of adenylyl cyclases (Fig. 7A and fig. S8, A and B). In addition, we found significant down-regulation of the zebrafish orthologs of mammalian activity-induced immediate early genes (IEGs) (*61*), including *fosab*, *junba*, and *bdnf* (Fig. 7B), many of which are under the regulation of the cAMP-inducible transcription factor (TF) CREB (*62*–*64*) (Fig. 6A). Reduced *fosab* expression was validated by HCR staining (fig. S8C). Although decreased phosphodiesterase genes expression in *srrm3*^∆eMIC^ neurons might contribute to increased cAMP pathway tone, the down-regulation of IEGs and adenylyl cyclases is consistent with homeostatic adaptation to sustained neuronal activity (*65*, *66*) and prolonged engagement of the cAMP pathway (*67*, *68*).

We next measured cAMP levels directly using an enzyme-linked immunosorbent assay (ELISA) (*69*). Unexpectedly, at 6 dpf, steady-state cAMP concentrations were similar between *srrm3*^∆eMIC^ and WT larvae (Fig. 7C). Because steady-state cAMP is subject to tight autoregulation (*70*), this may reflect adaptive normalization rather than the absence of pathway alteration. We therefore examined earlier developmental stages and found that, in untreated *srrm3*^∆eMIC^ mutants, cAMP levels were elevated at 3 dpf but quickly normalized (Fig. 7C), consistent with time-dependent adaptation. This interpretation is further supported by reduced IEG expression at 6 dpf (Fig. 7B). In line with this adaptive model, pharmacological elevation of cAMP in WT larvae showed a marked increase at 1 hour followed by a gradual return to control levels by ∼43 hours, despite continued stimulation (fig. S8, D and E).

We next investigated activation of key effectors of the cAMP pathway, possibly less affected by rapid adaptation. When cAMP binds the regulatory subunit of PKA, the catalytic subunit (PKA-C) is released to phosphorylate CREB at Ser^133^ (pCREB), enabling CREB-dependent transcription (Fig. 6A) (*15*). Consistently, pharmacological inhibition of PKA with H-89 rescued *srrm3*^∆eMIC^ hyperactivity while leaving WT sibling activity largely unchanged (Fig. 7D and fig. S8F), closely mirroring the effects of adenylyl cyclase inhibition (Fig. 6C). Western blotting revealed significantly elevated phosphorylation of PKA-C at Thr^197^ (pPKA-C), required for its activation (Fig. 7E). However, pCREB levels in the *srrm3*^∆eMIC^ were markedly reduced (Fig. 7E), consistent with the decreased expression of CREB target genes (Fig. 7B and fig. S8C). Together, these biochemical, transcriptional, and pharmacological data reveal misregulation of the cAMP-PKA-CREB pathway in *srrm3*^∆eMIC^.

### *srrm3*^∆eMIC^ transcriptomic signatures are partially mimicked and reversed by cAMP pharmacological modulation

**Fig. 8.**
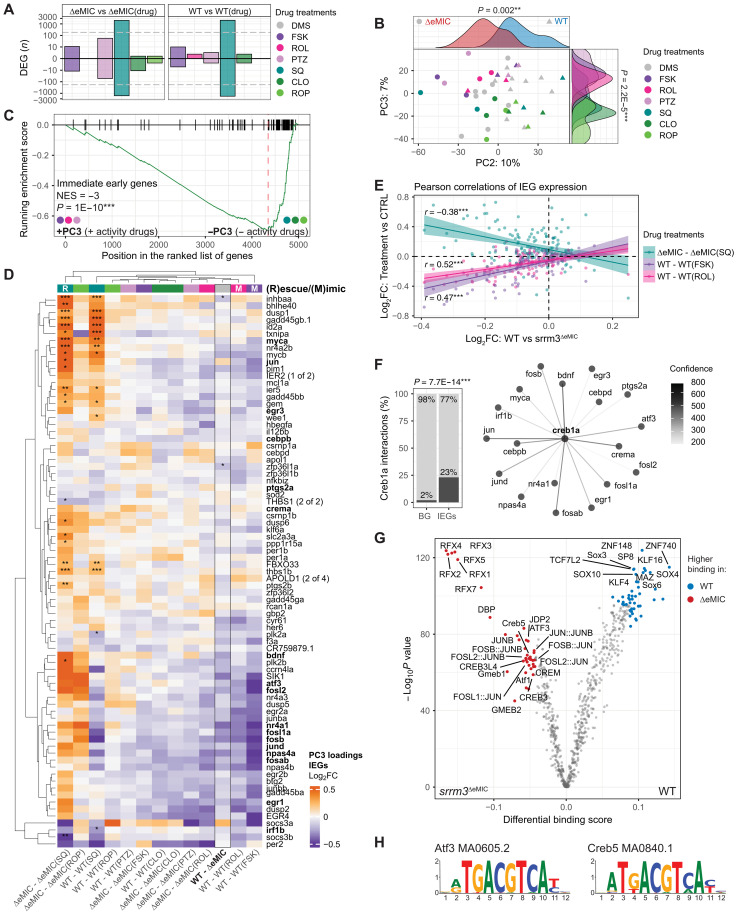
cAMP pathway modulators partially mimic or reverse *srrm3*^∆eMIC^-induced gene expression changes. (**A**) DEG number by comparison (pseudo-log_10_ scale). Gray line: number of DEGs for WT versus *srrm3*^∆eMIC^ (*N* = 8 biological replicates). Drug treatments: *N* = 2 biological replicates (data S4). (**B**) PCA of top 5000 variable genes after variance stabilization and batch correction. PC2 separates WT and *srrm3*^∆eMIC^ (*W* = 6, *P* = 0.002, Wilcoxon test). PC3 separates activity-increasing (PTZ, ROL, and FSK) from activity-reducing (ROP, SQ, and CLO) treatments (*W* = 6, *P* = 2.2 × 10^−5^, Wilcoxon test). Points: samples shaped by genotype and colored by treatment. (**C**) GSEA of PC3 loadings using mouse ortholog IEGs (*61*) as the test set. (**D**) Heatmap of IEG log_2_FC across treatments. Color bar indicates conditions [M, behavioral mimic; R, behavioral rescue (Fig. 6)]. Log_2_FC capped at ±0.5 for visualization. Creb1a interactors (F) in bold. (**E**) Correlations between IEG log_2_FC (WT versus *srrm3*^∆eMIC^; *x* axis) and treatment effects (*y* axis): mimics correlate with the mutant pattern [WT versus WT(FSK): *r* = 0.47 (*P*.adj = 1.8 × 10^−7^); WT versus WT(ROL): *r* = 0.52 (*P*.adj = 3.8 × 10^−9^)], whereas the rescue anticorrelates [∆eMIC versus ∆eMIC(SQ): *r* = −0.38 (*P*.adj = 6.9 × 10^−5^)]. Lines show linear regression fits ± SE. Pearson with Bonferroni correction. (**F**) IEGs are enriched for Creb1a interactors (OR = 14.8, *P* = 7.7 × 10^−14^; two-sided Fisher’s exact test). Network shows interactions (STRING). Edges indicate interaction strength (200 to 1000 confidence). (**G**) Differential TF motif binding (points = 746 vertebrate motifs) from ATAC-seq using TOBIAS for consensus sites of open chromatin (data S3). Blue: higher in WT; red: higher in *srrm3*^∆eMIC^; gray: n.s. *N* = 1 biological replicate per genotype (10 to 12 larvae per sample). (**H**) Selected activity-dependent TF motifs with differential binding in (G). Treatments: CLO, clonidine; DMSO, dimethyl sulfoxide (control); FSK, forskolin; PTZ, pentylenetetrazol; ROL, rolipram; ROP, ropinirole; SQ, SQ22536.

Given that cAMP pathway–modulating drugs both phenocopy and rescue the *srrm3*^∆eMIC^ daytime hyperactivity (Fig. 6, C to E), we next asked whether these behavioral effects correspond to shared transcriptomic signatures. For this purpose, we used bulk RNA-seq of FACS-sorted *elavl3*:GFP^+^ cells (predominantly neurons) of *srrm3*^∆eMIC^ and WT siblings treated with drugs that increase cAMP levels (the adenylyl cyclase activator forskolin, the phosphodiesterase inhibitor rolipram, and PTZ, which indirectly raises cAMP by increasing neuronal activity) and drugs that decrease cAMP levels (the adenylyl cyclase inhibitor SQ22536 and the G_i/o_-coupled GPCR agonists clonidine and ropinirole). Each condition was matched to its within-genotype and within-clutch dimethyl sulfoxide (DMSO) control (Fig. 4A). A principal components analysis (PCA) of all samples revealed that the largest source of variance (PC1: 17%) corresponds to treatment with SQ22536 versus all other conditions (fig. S9A). This is in line with the large number of DEGs identified upon SQ22536 treatment for both genotypes (*P* < 0.05; ∆eMIC: *n* = 3619, WT: *n* = 3829) and contrasts with the relatively few DEGs induced by all other treatments (Fig. 8A and fig. S9B; mean = 17 genes, range = 0 to 109 genes) (data S6). SQ22536-induced DEGs were enriched for terms related to neural development (fig. S9C), as previously reported in zebrafish (*71*). Components of the adenylate cyclase-modulating GPCR pathway were also enriched among up-regulated genes upon SQ225536 treatment of either WT or *srrm3*^∆eMIC^ (fig. S9C), highlighting a transcriptional effect at the level of cAMP synthesis.

Beyond the dominant effect of SQ22536 (captured by PC1), we observed coherent transcriptomic patterns corresponding to treatment type. PC2 separated samples by genotype (*P* = 0.002, Wilcoxon test), whereas PC3 distinguished treatments according to their behavioral effects: activity-promoting drugs (forskolin, rolipram, and PTZ) versus activity-reducing drugs (clonidine, ropinirole, and SQ22536) (Fig. 8B; *P* = 2.2 × 10^−5^, Wilcoxon test). Notably, GSEA using PC3 loadings as the background revealed that IEGs were significantly depleted among treatments that increased activity (Fig. 8C), consistent with adaptive down-regulation after prolonged stimulation (18 to 20 hours) (*66*, *72*). This pattern parallels the reduced IEG expression in *srrm3*^∆eMIC^ larvae (Fig. 7B) and the return of cAMP levels to baseline during extended rolipram exposure (fig. S8E). Clustering analysis supported these relationships as transcriptional profiles of activity-inducing treatments (forskolin and rolipram) grouped with those of *srrm3* depletion (Fig. 8D) and IEG changes in WT following these treatments correlated positively with those observed in *srrm3*^∆eMIC^ larvae (Fig. 8E and fig. S8D). Conversely, SQ22536, which mitigated *srrm3*^∆eMIC^ daytime hyperactivity (Fig. 6C), significantly reversed many IEG alterations in the *srrm3*^∆eMIC^ (Fig. 8E and fig. S9, D and E), with similar rescue patterns among genes annotated with “cAMP metabolic processes” (fig. S9F). These results implicate cAMP signaling in these transcriptomic responses.

Multiple IEGs encode TFs, including the AP1 family members (Fos and Jun), whose DNA binding motifs partially overlap with CREB (*73*), downstream of cAMP-PKA and a major regulator of activity-induced transcription (*62*–*64*). Moreover, an exceedingly high proportion of IEGs interact at the protein level with Creb1a (Fig. 8F; 23% versus 2%; *P* = 6.7 × 10^−15^, two-sided Fisher’s exact test). We therefore asked whether the persistent hyperactivity of *srrm3*^∆eMIC^ larvae is associated with altered TF chromatin occupancy. Assay for Transposase-Accessible Chromatin using sequencing- (ATAC-seq) on FACS-sorted *elavl3*:GFP^+^ cells from enucleated 6 dpf *srrm3*^∆eMIC^ and WT larvae revealed increased occupancy at AP1 motifs (Atf3, Junb, and Fosb::Jun) (*74*) and CREB-like motifs in *srrm3*^∆eMIC^ compared to WT (Fig. 8, G and H; fig. S9, G to I; and data S3) despite reduced pCREB and transcript levels of some of these factors (Fig. 7, B and E). This pattern parallels findings in mice, in which AP1 factors maintained prolonged chromatin engagement long after initial neuronal activation (*72*). Together with the partial mimicry and rescue of IEG and cAMP pathway gene expression by pharmacological manipulation of cAMP signaling, these data indicate that *srrm3*^∆eMIC^ neurons undergo persistent activity-induced chromatin remodeling, reflected in enhanced AP1 and CREB motif occupancy.

## DISCUSSION

**Fig. 9.**
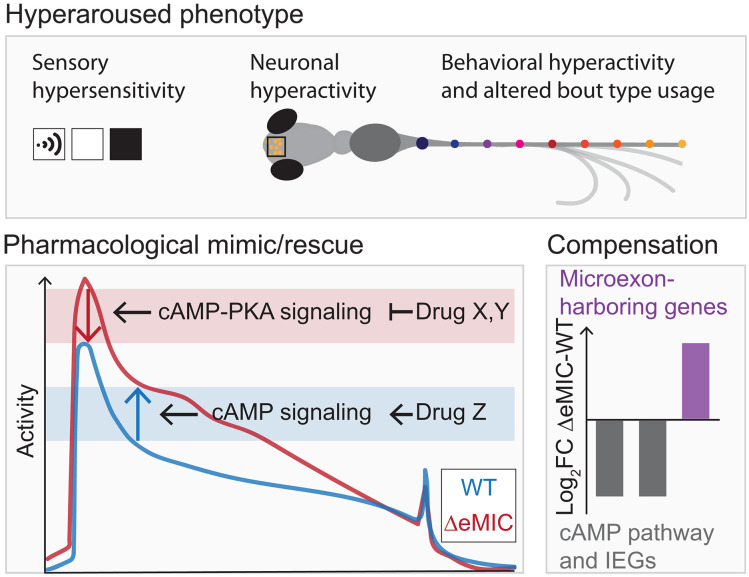
Model summary. *srrm3*^∆eMIC^ larvae show hyperarousal, reflected by increased responsiveness to sensory stimuli (phasic hyperarousal) and heightened neuronal and behavioral activity at baseline (tonic hyperarousal). The daytime hyperactivity can be rescued upon inhibition of cAMP-PKA signaling and can be mimicked upon activation of cAMP signaling in WT larvae. Gene expression changes are suggestive of compensatory mechanisms and homeostatic regulation with microexon-harboring genes being up-regulated in the srrm3^∆eMIC^, but the cAMP pathway and IEGs, downstream of cAMP-PKA-CREB signaling, are down-regulated.

*srrm3*^∆eMIC^ larvae display a constellation of behavioral and neuronal phenotypes—including reduced sleep, sensory hypersensitivity, altered swim-type usage, occasional seizure-like swims, and elevated forebrain activity—that together indicate a persistent shift in arousal regulation (Fig. 9). Rather than reflecting a nonspecific locomotor defect, these patterns point to dysregulation of both tonic (baseline) and phasic (stimulus-evoked) arousal (*3*, *14*). Despite visual impairment (*37*), *srrm3*^∆eMIC^ larvae showed prolonged behavioral responses to visual and mechanical stimuli compared to WT siblings, a finding indicative of heightened phasic arousal. In parallel, the increased prevalence of stress-associated swims at baseline and fragmented sleeping patterns suggest elevated tonic arousal, aligning with the overall hyperactivity and consistent with elevated spontaneous brain activity. These results parallel findings in *Drosophila* eMIC mutants that also display sleep loss and increased seizure susceptibility (*21*), pointing toward a deeply conserved role for neuronal microexons in shaping arousal states across species.

Sleep disturbances and sensory hypersensitivity are frequently comorbid in neurological disorders including schizophrenia and ASD (*5*, *34*), where microexon mis-splicing is also commonly observed (*19*, *30*–*33*). However, the contribution of microexon dysregulation to clinical manifestations of these conditions has remained largely unclear. Although we relied on activity-based rather than electrophysiological sleep readouts, our data support a role for microexon mis-splicing in arousal regulation, encompassing both sleep-wake patterns and neuronal excitability, consistent with previous reports linking microexons to altered neuronal excitability (*30*, *31*). Until this study, little was known about the molecular signaling cascades that connect global microexon disruption to altered excitability and behavior. Taking advantage of behavioral pharmacology, we identified cAMP-PKA signaling, downstream of conserved arousal pathways such as the dopaminergic and noradrenergic system, as a central driver of *srrm3*^∆eMIC^ daytime hyperactivity and possibly arousal. Manipulating this pathway bidirectionally modulated locomotor activity, with cAMP-PKA inhibition normalizing *srrm3*^∆eMIC^ mutant hyperactivity and cAMP elevation inducing hyperactivity in WT larvae (Fig. 9). These findings align with previous research implicating cAMP-PKA signaling in the regulation of arousal and anxiety across species. Elevated cAMP levels induce hyperlocomotion in both zebrafish larvae and mice (*69*, *75*). Furthermore, in zebrafish, forskolin treatment triggers excessive thigmotaxis (*69*) and a shift from short-latency to long-latency startle responses (*57*), whereas stressed mice show higher levels of PKA phosphorylation (*76*). In humans, dysregulated cAMP-PKA signaling has also been associated with anxiety disorders (*77*). These phenotypes closely mirror those observed in *srrm3*^∆eMIC^ larvae, including anxiety-like behaviors, hyperlocomotion, and a high usage of stress-associated long-latency escapes.

Persistent activation of the cAMP-PKA pathway is known to engage strong homeostatic feedback mechanisms (*70*), including down-regulation of adenylyl cyclases after prolonged stimulation (*67*, *68*). Consistent with this, steady-state cAMP levels at 6 dpf were similar between genotypes, despite being elevated in the *srrm3*^∆eMIC^ mutant larvae at 3 dpf. Moreover, pharmacologically induced cAMP increases in WT larvae returned to baseline within hours despite continuous stimulation, together pointing to robust homeostatic control. At the transcriptional level, we observed reduced expression of cAMP pathway genes and several IEGs, including *fosab*, *bdnf*, and *nr4a1*, all of which are CREB regulated (*62*–*64*) (Fig. 9). These results likely reflect an adaptive response to persistent cAMP-PKA-CREB activation and elevated neuronal activity rather than diminished signaling. The reduced expression of CREB-dependent IEGs is mirrored at the protein level. Although pPKA-C was elevated in *srrm3*^∆eMIC^ larvae, pCREB levels were paradoxically reduced. CREB phosphorylation and IEG levels rise rapidly following transient neuronal activation, including after forskolin treatment (*78*, *79*). However, sustained activity is known to blunt subsequent CREB phosphorylation and IEG induction in multiple contexts including seizure models (*66*, *72*, *80*), chronic stress (*65*, *81*), and following injury (*82*). The dissociation between PKA activation and pCREB could arise from shifts in the balance of other CREB-regulating kinases and phosphatases (e.g., CaMKs and PP1/PP2A) (*80*, *83*, *84*) or from chromatin-dependent constraints on CREB phosphorylation dynamics (*79*). Consistent with this broader activity-dependent adaptation, ATAC-seq revealed increased occupancy at AP1 and CREB-like motifs, indicating altered engagement of activity-dependent TFs. This is reminiscent of transcriptional priming or metaplasticity, in which prolonged neuronal activity leaves a molecular footprint that shapes future gene responsiveness (*72*, *85*). For example, seizure-like activity in mice induces prolonged AP1 motif occupancy and a subsequent blunting of *C-fos* induction (*72*), closely paralleling the diminished *fosab* expression we observed in *srrm3*^∆eMIC^ larvae.

How does *srrm3*-dependent microexon misregulation lead to the observed molecular, cellular, and behavioral phenotypes? In principle, two nonmutually exclusive models could explain the *srrm3*^∆eMIC^ hyperarousal phenotype: (i) a dominant contribution from a small subset of functionally critical microexons or (ii) the combined impact of widespread microexon mis-splicing. Our analyses of hyperactivity and sleep-wake cycles for nine individual microexon mutants revealed largely no or mild phenotypes, in line with previous studies in zebrafish (*29*, *55*) and the proposed role of microexons in fine-tuning and specializing protein function rather than drastically modifying it (*86*). Nevertheless, analysis of individual microexons can provide mechanistic insight into specific components of the hyperarousal phenotype. For example, *rapgef2*, a key mediator of GPCR-driven cAMP signaling (*87*), contains several microexons. Mis-splicing of one such microexon (24 nt) is sufficient to increase daytime locomotor activity, partially overlapping with the *srrm3*^∆eMIC^ behavioral phenotype. In addition, elevated intracellular calcium can promote cAMP signaling through activation of calcium-sensitive adenylyl cyclases (*88*). In line with this, using 2P calcium imaging revealed elevated event amplitudes across brain regions and stimulus conditions for *srrm3*^∆eMIC^ neurons, indicative of increased calcium influx. Several genes involved in calcium ion homeostasis, including *micu1*, whose pharmacological targeting leads to differential effects in *srrm3*^∆eMIC^ and WT larvae, and the calcium channel gene *cacna1da* harbor mis-spliced microexons with previously reported effects on protein function (*56*, *89*), suggesting that disruption of calcium signaling may substantially contribute to cAMP dysregulation and hyperarousal. Last, mis-spliced microexons in calcium- and calmodulin-dependent protein kinase II (CaMKII) subunit genes, including in *camk2d1*, *camk2d2*, and *camk2g2*, with the latter known to affect nuclear translocation of CaMKII (*90*), could affect CREB phosphorylation states (*84*), providing yet another, nonmutually exclusive, mechanism for cAMP pathway alteration.

However, several lines of evidence argue against a model in which disruption of one or a few microexons is sufficient to account for the full *srrm3*^∆eMIC^ phenotype. First, individual microexon perturbations often produce opposing effects. For example, *ptprd* microexon mutants in zebrafish display reduced locomotor activity (*55*), opposite to the hyperactivity in *srrm3^∆eMIC^* larvae. Similarly, opposing effects have been reported for individual microexons in neurite growth (*29*) or exocytosis (*91*). Second, approximately one-third of up-regulated genes in *srrm3*^∆eMIC^ harbor mis-spliced microexons. This widespread transcriptional response supports a phenotypic contribution of multiple microexons and is consistent with compensation following altered protein function rather than NMD (*23*, *92*). The compensation hypothesis is supported by most *srrm3*-dependent microexons predicted to preserve the reading frame and by known effects of microexon inclusion for some of these up-regulated transcripts: Microexon inclusion can strengthen protein-protein interactions (e.g., in the presynaptic organizers Ptprd and Ptprfa) (*93*), enhance protein activity (e.g., via increased Ptk2 autophosphorylation) (*94*), or modulate Ca^2+^ handling (e.g., via increased mitochondrial Ca^2+^ uptake through Micu1 gating) (*56*). Further consistent with a compensatory role, genes hosting individually deleted microexons in zebrafish are significantly more often up-regulated than down-regulated and typically show no or only mild phenotypes (*29*). Secondary changes in transcript abundance upon loss of splicing factor function have been described [e.g., (*37*, *95*)]; however, to our knowledge, such a strong up-regulation of mis-spliced transcripts has not been reported before. Determining to what extent this transcriptional up-regulation functionally compensates for the loss of each individual microexon-mediated protein feature will require future mechanistic studies.

Together, these observations indicate that, although individual microexons can influence discrete molecular or behavioral features, the *srrm3*^∆eMIC^ hyperarousal phenotype is unlikely to be explained by loss of any single microexon. Rather, our findings support a model in which the cumulative mis-splicing of many functionally diverse neuronal microexons, accompanied by likely compensatory transcriptional responses, underlies the phenotype.

In summary, the widespread disruption of microexon splicing in *srrm3*^∆eMIC^ affects diverse proteins involved in vesicle transport, endocytosis, ion channel function, and cytoskeletal organization, ultimately impairing cAMP-PKA-CREB signaling, dysregulation of which provides a plausible mechanistic explanation for the heightened arousal phenotype of *srrm3*^∆eMIC^ larvae. Given recurrent mis-splicing of microexons in various neurological disorders (*19*, *30*–*33*), many of which show dysregulated arousal states (*5*, *34*), future research may focus on correcting these splicing defects. Potential strategies include indirect modulation of downstream pathways, such as targeting cAMP-PKA-CREB signaling as proposed for depression (*96*), or direct correction of mis-spliced individual (*97*) or groups of microexons (*98*) using antisense oligonucleotides. Moreover, pharmacological modulation of microexon splicing, in part via master regulators, represents a promising therapeutic avenue (*99*). Together, our findings uncover neuronal microexons as modulators of vertebrate arousal state via cAMP-PKA-CREB signaling, offering insight into how their dysregulation may contribute to hyperarousal, characterized by sensory and sleep-wake disturbances relevant to neurodevelopmental disorders.

### Limitations

This work has several limitations that point to important directions for future research. First, the relationship between microexon mis-splicing and hyperarousal remains largely correlative. Although *srrm3* is the master regulator of neuronal microexons and these exons are the most selectively disrupted splicing class in *srrm3*^∆eMIC^, at 5 to 6 dpf, many genes are differentially expressed, and we cannot assign specific behavioral phenotypes to individual microexons or exclude secondary contributions from transcriptional changes. Similarly, although we robustly show misregulation of the cAMP-PKA-CREB pathway, the upstream trigger of this dysregulation remains unresolved. We outline several candidate mechanisms in Discussion, but establishing the causal chain from loss of the microexon program to altered signaling will require additional targeted perturbations, such as rescue experiments and single or combinatorial microexon mutants. Last, our data do not allow us to clearly separate developmental miswiring from acute, ongoing signaling effects. Multiple mis-spliced microexons influence neurodevelopmental processes such as neurite outgrowth, suggesting that early disruption may contribute to the phenotype at 5 to 6 dpf. At the same time, we observe clear evidence for acute dysfunction: ongoing microexon mis-splicing at 6 dpf, elevated neuronal activity, misregulated cAMP-PKA-CREB signaling, and robust pharmacological rescue. Future work will require temporally controlled manipulations to disentangle the phenotypic contribution of early developmental alterations versus acute signaling imbalances.

## MATERIALS AND METHODS

### Experimental design

The objective of this study was to determine how disruption of the microexon master splicing regulator *srrm3* affects arousal regulation and underlying molecular pathways in zebrafish. We combined genetic, behavioral, pharmacological, biochemical, and transcriptomic approaches to assess arousal phenotypes and their mechanistic basis. Experiments were designed to compare genotypes and treatment conditions using appropriate controls and biological replicates.

### Zebrafish husbandry, lines, and genotyping

All procedures complied with national and institutional regulations at the PRBB, UCL, and the Champalimaud Foundation and followed EU Directive 2010/63/EU. They were approved by the Barcelona Biomedical Research Park Institutional Animal Care and Use Ethic Committee Institutional Animal Care and Use Ethic Committee (PRBB-IACUEC) (MIR-23-0042), UCL Animal Welfare Ethical Review Body, and UK Home Office under the Animal (Scientific Procedures) Act 1986 and by Champalimaud Foundation Ethics Committee and the Portuguese Direcção Geral Veterinária (0421/000/000/2019), respectively. Zebrafish (*Danio rerio*) were maintained at 28° to 28.5°C on a 14-hour light/10-hour dark cycle at the PRBB or for the 2P functional imaging at UCL. Crosses were set from heterozygous parents to enable within-clutch comparisons of WT, heterozygous, and mutant siblings for all experiments. Embryos were raised in E3 medium (with or without methylene blue for imaging experiments) at ∼50 larvae per plate at 28°C on a 14-hour light/10-hour dark cycle (lights on: 9:00 a.m.). Experiments were performed at 4 to 8 dpf; sex cannot be determined at these stages. Genotyping followed established protocols (*29*), using fin clips at 3 dpf (*100*) or whole larvae after experiments. WT larvae were from the AB line (ZFIN: ZDB-GENO-960809-7). The main founder used for this study (referred to as *srrm3*^∆eMIC^) (crg3, ZFIN: ZDB-ALT-230328-5) was created in a Tg(*elavl3*:GFP) background carrying a 5–base pair (bp) deletion in the eMIC domain disrupting the open reading frame (ORF). All other lines are described in the Supplementary Materials.

### Behavioral experiments using the DanioVision tracking system

#### DanioVision setup and tracking

Behavior was recorded in the DanioVision chamber (Noldus Information Technology, Wageningen, The Netherlands) using EthoVision XT (v15-17) at 25 Hz, with the chamber temperature set to 28°C. Larvae were placed individually in E3 medium; genotypes were randomized and determined postexperiment (*29*). Most assays used 96-well square plates (650 μl; Whatman 7701-1651 UNIPLATE), except thigmotaxis, which used 24-well circular plates (1 ml; Nunc Cell-Culture treated multidishes, Thermo Fisher Scientific, 142475). Larvae that postexperimentally failed to respond to tactile stimulation with a P200, were malformed, or had unclear genotypes were excluded. Activity (%∆pixels) was analyzed using pixel-based detection with thresholds adjusted per lighting condition (18 to 20 pixels). For thigmotaxis, center-point tracking with automatic detection settings was used.

#### Sleep-wake experiments and analysis

Larvae were plated the evening before experiments at 4 to 5 dpf and tracked for up to 74 hours under a 14-hour light/10-hour dark cycle [lights on: 9:00 a.m., zeitgeber time 0 (ZT0)]. Light intensity was kept at 5% of the operating system [∼500 lux; light meter (UT382 USB, H160575608)]. E3 was topped up each morning between ZT0 and ZT1, with the exception of *vav2* and *vti1a* microexon mutant lines for which water was topped up at ZT4. To compare *srrm3*^∆eMIC^ to published microexon mutants [(common) gene name + VastID]: *ppfia4* (DreEX0057348), *ppp6r3* (DreEX0057914), *ptprd-1* (DreEX0002174, MeB), *ptprd*-2 (DreEX1003032, MeA6), and *rapgef2-1* (DreEX0060838), raw data from nights 4 to 5 and 6 to 7 dpf and days 5 to 6 dpf were reanalyzed (*55*). In-house data were analyzed for nights 5 to 7 dpf and day 6 dpf using FramebyFrame v1.1.0 (*101*) in R after converting DanioVision files to Zebrabox Viewpoint format. Sleep was defined as ≥1-min immobility (*10*, *101*). Quality control in R used vpSorter followed by ggActivityTraceGrid. Behavioral parameters and *z*-scores for the heatmaps were obtained with multiBehaviourParameter and calculateFingerprint, respectively. Statistics used the likelihood ratio test on LMMs: lmer(values ∼ Group/Genotype + (1|Clutch), data = night or day) (data S1). Percent changes were derived from model estimates following published formulas (*101*), where the *y* intercept represents the estimated WT mean and the WT mean plus the estimate represents the ∆eMIC mean: for positive WT mean: 100 × (mean_∆eMIC/mean_WT) − 100; for negative WT mean: [(100 × (mean_∆eMIC/mean_WT) − 100] × (−1). For activity-sleep traces, for visualization water-refill intervals were replaced by the preceding time window.

#### Photomotor assay

Larvae were plated in the morning and acclimated for 1.5 hours and then presented with alternating 1-hour dark and 1-hour bright light (100%, ∼10,000 lux) over 10 hours. Responsiveness (mean difference) was calculated as the difference in activity (%ΔPix) 1 min before versus after each transition, averaged per larva. Statistics by Satterthwaite-approximated *t* tests on the LMM in R: lmer(mean difference ∼ Genotype + (1|Clutch)).

#### Mechanical tapping habituation assay

At daytime, after 1-hour acclimatization, larvae received 10 high interstimulus interval (ISI) (90 s) taps (prehabituation condition) followed by 100 low-ISI (5 s) taps (habituation condition) at maximum power (*8*). Only larvae responding to ≥7/10 high-ISI taps were analyzed. Responses were defined as activity (%ΔPix) within 1 s after tap > 1 s before tap. HI followed (*102*) by calculating the difference in response probability for the last 40 low-ISI taps to the response probability during the first 10 high-ISI taps: HI = 1 – (Plast40 / Pfirst40) Statistics by Satterthwaite-approximated *t* tests on the LMM in R: lmer(HI ∼ Genotype + (1|Clutch)).

#### Thigmotaxis assay

Larvae were assayed across three technical replicates (ZT1 to ZT9) to maximize the sample size (*n* = 3 × 24). After 30-min acclimatization at full light (∼10,000 lux, 100%), locomotion was recorded for 1 hour. Larvae with poor tracking [Pearson correlation < 0.9 between activity (%ΔPix) and distance moved (mm)] were removed (23/144). Wells were divided into center [radius (*r*) = 5.63 cm] and periphery (*r* = 8 cm), yielding ∼100 cm^2^ per region. Thigmotaxis (% TDM) = (TDM_Periphery / TDM_WholeArena) × 100. Statistics by Satterthwaite-approximated *t* tests on the LMM in R: lmer(thigmotaxis ∼ Genotype + (1|Clutch)).

### High-resolution tail tracking and bout type analysis

#### Experimental design and setup

Experiments were conducted in the Orger lab at the Champalimaud Centre for the Unknown (Lisbon, Portugal). Larvae from *srrm3*^+/∆eMIC^ incrosses were shipped at 24 to 48 hours postfertilization (hpf) and raised in groups of ∼100 on large petri dishes (*r* = 14 cm) in a 14-hour light/10-hour dark incubator. Behavior was tracked at 5 to 6 dpf across four clutches (C1 to C4) using the setup described in (*35*, *45*). Experiments were performed blind to genotype, which was determined posttracking (*29*). Larvae acclimated for 5 min before recording a 25-min baseline under constant illumination (∼1500 lux). For clutches C1, C3, and C4, this was followed by alternating 5-min dark/light periods; for C1 and C2, an alternative 15-min recording was performed under constant light in 10 mM PTZ (stock 100 mM in E3; Sigma-Aldrich, P6500-25G). Data preprocessing and bout classification followed (*35*, *45*) and are detailed in the Supplementary Materials.

#### Bout type usage

Larvae with <5 bouts/min during baseline, dark, or light periods were excluded (*n* = 2 across four clutches) and *n* = 1 as kinematics parameter outlier (data S2). For each larva, percentage bout type usage was calculated for baseline (25 min; *N* = 4 clutches) and dark (15 min total; *N* = 3 clutches). Statistics using a negative binomial GLMM in R on bout counts with glmmTMB v1.1.13: glmmTMB(count ∼ Genotype + (1|Clutch)). Percentages and SE were obtained from log-linear estimates (*103*) using: percentage = (exp(estimate) − 1) × 100; percentage SE = exp(estimate) × estimate SE × 100. *P* values were Bonferroni corrected for the number of bout types tested per condition (data S2).

#### Dark-light escape response latencies

Analysis was performed on the same larvae as for “Bout type usage” (data S2). The mean escape latency was computed by measuring the duration (ms) until the first escape (O-bend, SAT, LLC, and SLC) within a 3-s window after the light-dark transition (C1, C3, and C4: *n* = 3 transitions). Larvae with no escapes within this 3-s window were excluded (*n* = 5; data S2). Statistics used the same LMM structure as in “Bout type usage” above: lmer(log(latency) ∼ Genotype + (1|Clutch), data *=* data). Percentages and SEs were estimated as in “Bout type usage.”

#### Bout kinematics

Analysis was performed on the same larvae as for “Bout type usage” (data S2). Bout duration was defined as end minus start time; displacement as sqrt((boutDistance*X*)^2^ + (boutDistance*Y*)^2^); speed as sqrt((boutSpeed*X*)^2^ + (boutSpeed*Y*)^2^); meanBoutFreqCorr as the number of detected half-betas divided by duration; boutAngle as the change in heading angle; and boutMaxAngularSpeed as the maximal tail-angle change (*35*). Bouts with |boutAngle| > 360° (20/600,469) and bouts missing any of the tested kinematic parameters (1530/600,449) were removed. For within-bout type analyses, larvae with <3 bouts of a given type were excluded (data S2). Kinematic parameters across and within bout types were compared using Satterthwaite-approximated *t* tests on the LMM in R: lmer(values ∼ Genotype + (1|Clutch)) and, when applicable, Bonferroni corrected for the number of bout types tested. Percentage differences were plotted using ComplexHeatmap (v2.20.0) as: [(mean_∆eMIC − mean_WT) / |mean_WT|] × 100.

#### Long bout analysis

Long bouts were defined as >500 ms. Tail-angle traces were plotted (Python) and manually annotated as noise/unclear or genuine long bouts, blinded to genotype. Statistical significance for kinematic comparisons was obtained by permutation testing using 1000 equally sized random bout sets (matched by genotype). For statistical details, see the Supplementary Materials.

### 2P calcium imaging

#### Setup and experimental design

Experiments were conducted in the Bianco lab at UCL (London, UK). The custom-built 2P imaging setup and experimental procedure were previously described (*104*). Larvae [nacre; *Tg(elavl3:H2B-GCaMP6s)^jf5^*] were embedded in 3% low-melting agarose with eyes and tail free to move the afternoon before imaging at 5 or 6 dpf. Visual stimuli were presented, and tails were tracked for behavioral readouts (*104*). Details are in the Supplementary Materials.

#### Image registration

Functional stacks were registered to the Zebrafish Brain Browser (ZBB) using ANTs (v2.3.5) following the multistep workflow of (*104*). Each functional volume was first aligned to a high-resolution anatomy stack (1-μm *z*-spacing) from the same larva; the anatomy stack had been preregistered to the ZBB “huC_nls_mCar1.nrrd” ZBB reference (1x1x1 *x*,*y*,*z* μm/px), allowing concatenated transformations to place functional data into the ZBB space.

#### Calcium imaging analysis: Preprocessing

Data were processed using the standardized MATLAB pipeline described in (*104*). Motion correction (*105*) yielded >75% well-aligned epochs for all larvae except one ∆eMIC sample (40%), which was excluded from stimulus analyses but retained for baseline/gray screen with good alignments available. Regions of interest (ROIs) were extracted using a MATLAB script for cell detection (table S1), assigned to brain regions via anatomical masks, and manually adjusted as needed. Only ROIs within the *elavl3*^+^ brain mask were included. Grouping of anatomical regions: optic tectum (optic tectum–neuropil and optic tectum–stratum periventriculare), hindbrain (corpus cerebelli, valvula cerebelli, and hindbrain), pretectum, tegmentum, habenula, and pallium.

#### Swim bout kinematics in head-restrained larvae

Bout frequency was computed per ∼30-s epoch and bout median across all stimulus epochs, including those where neuronal data were discarded for motion artifacts. Statistics in R used a GLM: glm(parameter ∼ Genotype + stimulus + ID).

#### Calcium imaging analysis: Event amplitude, frequency, and event total

Neuronal activity was inferred from deconvolved GCaMP6s calcium traces using OASIS (*51*) in MATLAB. Inferred spike trains (events) were used for downstream analysis in R. Because spike inference depends on signal-to-noise and indicator brightness, we measured GCaMP6s fluorescence at 800 nm (independent of neuronal activity) in representative ROIs across pallium, habenula, and optic tectum by calculating mean gray values using Fiji/ImageJ2 (v2.14.0/1.54f) (fig. S3G). ROIs with extreme event amplitudes [>99 arbitrary units (AU)] were removed, likely representing artifacts (*51*). Event metrics were calculated per stimulus (light, dark, LD, and LDC; minimum: two epochs) or baseline (gray; last ∼6 min) for each ROI:

1) Mean event amplitude (AU): mean nonzero event amplitude.

2) Event frequency (events/s): nonzero events per frame × 3.6 Hz (acquisition rate).

3) Mean event total: sum of events per window, normalized to a 12-frame (∼3 s) epoch.

Statistics used Wilcoxon tests at both ROI (neuron) and per-larva levels.

#### Calcium imaging analysis: Visualization

Empirical cumulative distribution functions (ECDFs) were computed using R stats per larva and averaged on a common *x*-grid (means ± SEM; truncated at *y* = 0.97). For anatomical maps, ROI coordinates were binned in 10-μm cubes, e.g., ROIs coordinates within *x* = 10 to 20, *y* = 10 to 20, and *z* = 10 to 20 were assigned *x* = 15, *y* = 15, and *z* = 15. Mean values per bin and genotype were computed, bins with <3 ROIs were removed, and ∆eMIC bin averages were subtracted from WT.

### Bulk and single-cell RNA-seq

#### Sample preparation, dissociation, and FACS

Genotype comparisons used sibling larvae; drug-treatment comparisons used within-genotype, within-clutch siblings (data S4). Bulk RNA-seq for splicing analysis was performed on nonenucleated 5 dpf larvae. All other sequencing experiments used 6 dpf larvae, age-matched to sleep-wake assays, that were enucleated before dissociation to avoid gene expression changes due to photoreceptor degeneration (fig. S4A) (*37*). Larvae were dissociated and then FACS sorted following (*29*). Details are in the Supplementary Materials.

#### RNA extraction before bulk RNA-seq

RNA from FACS-sorted *elavl3*:GFP^+^ neurons was isolated using two different methods: (i) TRIzol extraction for bulk RNA-seq used in splicing analysis. Cell pellets were centrifuged for 3 min at 5000 rpm, resuspended in TRIzol (Thermo Fisher Scientific, 15596018), and frozen at −80°C. RNA was extracted using Phasemaker tubes (Invitrogen A33248) following the manufacturer’s protocol. Briefly, lysates were bead beaten (2 × 30 s, 5-min break on ice), phase separated, precipitated with glycogen, and eluted in RNase-free water. (ii) PicoPure extraction for all other datasets. RNA was isolated using the PicoPure Mini kit (Thermo Fisher Scientifc, KIT0204). Pellets were lysed in extraction buffer (30 min, 42°C), and cleared lysates were processed according to the manual: RNA was bound to PicoPure columns, washed, DNase treated (Qiagen, 79254), further washed, and eluted in 30-μl elution buffer.

#### Bulk RNA-seq

RNA (20 to 50 ng) was handed to the CRG Genomics Facility for library preparation using the SMART-Seq v4 Ultra Low Input RNA kit followed by NEBNext Ultra II. Samples for splicing analysis were sequenced as 125- to 150-bp paired-end reads (∼80 million reads per sample), and drug/DMSO-treated samples were sequenced as 50-bp single-end reads (∼35 million reads per sample) on Illumina platforms. Full sequencing metadata are in data S4.

#### Single-cell RNA-seq and data processing

Single-cell suspensions were generated from freshly FACS-sorted *elavl3*:GFP^+^ cells (see “Sample preparation, dissociation, and FACS”) and loaded onto the 10x Genomics Chromium platform (Next GEM 3′ v3.1). Libraries were sequenced on an Illumina NextSeq 500 (paired-end, 150 bp), using 150 cycles on either 66% (batch 1) or 100% (batch 2) of a high-output flow cell. Each sample contained ∼13,000 cells (345 cells/μl) targeting a recovery of ∼9000 cells. Raw data were processed with cellranger v6.1.2. A custom *D. rerio* GRCz10 reference was generated with cellranger mkref from the *D. rerio* (zebrafish) genome assembly version GRCz10.9 and the gene transfer format (gtf) file [protein-coding genes only (gene_biotype=protein_coding)] using cellranger mkgtf. Reads were aligned and unique molecular identifiers (UMIs) were counted with cellranger count, including intronic reads, with an expected recovery of 9000 cells.

#### Single-cell clustering and downstream analysis in R

After sequencing (145-172M reads per sample), we obtained 14,240 cells (WT1: 4024; WT2: 3200; ∆eMIC1: 2946; ∆eMIC2: 4070) and applied quality filtering in R using isOutlier (median absolute deviation > 3) from scater (v1.32.0) as guideline (>300 genes, <5% mitochondrial RNA, and 400 to 30,000 UMIs), retaining 12,862 cells (WT1: 3836; ∆eMIC1: 2808; WT2: 2785; ∆eMIC2: 3437). Analyses were performed with Seurat (v4.3.0): SCT transformation with regression of mitochondrial read percentage and selection of 3000 variable genes, batch integration, and PCA. Sixteen PCs (>70% variance explained; findPC v1.0) were used for Louvain clustering (nearest neighbors *k* = 30) on the integrated data (resolution 0.6; FindClusters), yielding 20 clusters. Cluster identities were assigned using Seurat marker genes [log_2_FC (fold change) > 0.25, >20% expression, Wilcoxon rank sum test; FindMarkers] and validated with the DanioCell database for single-cell gene expression of the zebrafish larva brain and other publications (*106*, *107*). A ribosomal-enriched cluster (Ribo^+^) was excluded. Genotype contributions were compared using propeller (speckle v1.4.0), with false discovery rate (FDR) correction. *srrm3* expression in WT clusters was visualized using ALRA (v0.0.0.9000) imputation. Differential expression for GSEAs was computed with Seurat FindMarkers (negative binomial model, batch covariate), retaining genes expressed in >30% of cells [Benjamini-Hochberg (BH)-adjusted]. GSEA was performed using clusterProfiler (v 4.12.0) and visualized with enrichplot (v 1.24.0). Cluster identities: Neur, neuronal; Glut, glutamatergic; GABA, GABAergic; Hb, habenula; Pallium, pallium; V-FB, ventral forebrain; Ganglia, cranial ganglion, Hypo, hypothalamus; Thal, thalamus; OT1, optic tectum 1; OT2, optic tectum 2; Purkinje, Purkinje cells (cerebellum); Granule, cerebellar granule cells; HB, hindbrain; HB(Glyc), hindbrain (glycinergic); Diff Neuro, differentiating neurons; OPC, oligodendrocyte precursor cells; Oligo, oligodendrocytes; Glia, glia (mature, microglia, and radial glia); Ribo^+^, ribosomal gene expression enriched.

#### Differential splicing analysis

Alternative splicing was quantified using vast-tools (v2.5.1). Paired-end reads were aligned to the *D. rerio* (vastdb.dre.23.06.20) with vast-tools align, merged with combine, and differential splicing was assessed with compare (parameters: --min_dPSI 15 --min_range 5 --paired --sp Dre --print_AS_ev --GO). *F*-statistics were computed using betAS (v1.2.0). Neuron-specific exons were taken from (*29*). ∆PSI (% change) was computed as (∆PSI ∆eMIC / ∆PSI WT) × 100. Exon-level protein-level impact annotations were obtained from VastDB and simplified into ORF-disrupting upon inclusion/exclusion, alternative protein isoform, or alternative untranslated region, excluding the categories “non coding” and “in the coding sequence with uncertain impact.” GO enrichment was performed in R using clusterProfiler with background genes from vast-tools compare (--GO). Cross-species comparisons (*22*, *24*) used orthologous human/mouse exons from VastDB, retaining only one-to-one relationships that passed coverage filters in both species. ∆PSI was calculated as: PSI(SRRM4 overexpression) − PSI(GFP control) and PSI(Srrm3/4 KD) − PSI(WT) for human and mouse, respectively.

#### Differential gene expression analysis

Reads were mapped to the *D. rerio* transcriptome (GRCz10) using Salmon (v1.5.1) with min accepted length for viable match = 25. Mapping rates were >60% with the exception of ∆eMIC-DMSO (run i) with 29% mapping and contamination (human and mouse) thus excluded (data S4). Count and counts per million (CPM) matrices were imported into R via tximport (v 1.32.0). Genes with >10 counts and CPM > 1 in at least one condition were retained. For the high-depth paired-end 125- and 150-bp datasets, differential expression was computed with DESeq2 (v1.44.00) (model: ∼ batch + genotype) and *P* values FDR corrected using fdrtools (v1.2.18) for bulk 1. For the shallower 50-bp dataset, limma (v3.60.0) was used with empirical Bayes moderation [model: ∼ genotype(treatment), eBayes] on DESeq2 (v1.44.0) VST-transformed (model ∼ 1) batch-corrected counts [empirical Bayes-moderated linear regression with batch as removed and genotype(treatment) as retained covariate; WGCNA (v1.72.5)]. GO (all terms, set size = 10 to 200) and GSEA analyses were conducted with clusterProfiler (v4.12.0), GSEA was plotted with enrichplot (v1.24.0), and Volcano plots were plotted with EnhancedVolcano (v1.22.0). For GO visualizations, terms with identical gene sets were removed. STRING protein-protein interaction networks were retrieved using STRINGdb (v2.16.0) with min interaction strength = 200 and visualized with igraph (v2.1.4) and ggraph (v2.2.1) using the 5000 most variable genes (PC loadings) as the background set. Chromosomal annotation used biomaRt (v2.60.1). To compute the sequence similarity between *srrm3*-regulated exon harboring genes and paralogous genes, paralogous gene sets were downloaded from the Ensembl biomart (v112) and a permutation test was used [*n* = 1000 permutations; *P* = (*r* + 1) / (*n* + 1); details are in the Supplementary Materials].

### In situ HCR

#### Probe design

HCR probes for *elavl3* were purchased from Molecular Instruments together with B1-Alexa647 and B2-Alexa546 amplifiers and required buffers. Custom probes for *fosab* and *srrm3* were designed and ordered from Integrated DNA Technologies following (*108*). Probe pairs failing GC%, melting temperature threshold, or predicted off-target criteria were excluded. Probe sequences and combinations (B2-*elavl3*; B1-*fosab*; B1-*srrm3*) are listed in data S5.

#### Experimental overview

Exp 1: *srrm3* expression in WT larvae (6 dpf; *N* = 1 clutch; fig. S4E).

Exp 2: Brain morphology via *elavl3* staining WT versus *srrm3*^∆eMIC^ (6 dpf; *N* = 3 clutches; fig. S4F).

Exp 3: Brain morphology and *fosab* expression with *elavl3* and *fosab* costaining WT versus *srrm3*^∆eMIC^ (6 dpf; *N* = 3 clutches; figs. S4F and S8C).

#### Fixation

Larvae were fixed at 6 dpf ∼ ZT1 to ZT3 for Exp 1 and 2 and ZT16 (after lights off) for Exp 3. Animals were transferred to a 40-μm strainer (Cultek, 88141378), briefly drained, and immediately immersed in 4% fresh paraformaldehyde (PFA) in 1 x phosphate-buffered saline (PBS). Fixation was performed either overnight at 4°C or 3 hours at room temperature with gentle rotation. Genotyping was carried out by fin-clip (3 dpf) (*100*) or postfixation in 1 x PBS. After fixation, larvae were dehydrated stepwise into 100% methanol for storage at −20°C. WT and *srrm3*^∆eMIC^ were processed in separate tubes.

#### HCR: Experimental procedure

The HCR staining was performed on larvae with a homozygous nacre background (*mitfaw2* allele) to avoid skin pigmentation as described in (*109*) adapted from (*108*). Up to 21 larvae were stained in the same tube. Detailed procedures are in the Supplementary Materials.

#### Mounting and imaging

Larvae (5 to 8 per dish) were mounted dorsal-down in 1.5% low-melting agarose (SeaPlaque Agarose, 50100) in 1 x PBS on glass-bottom dishes (35 mm; MatTek P35G-1.5-20-C). Imaging was performed on a Zeiss LSM 980 with an EC Plan-Neofluar 10×/0.30 objective, 1.2× scan zoom, bidirectional scanning, and GaAsP-PMT detection. *Z*-stacks were acquired at 4-μm spacing (5 μm for *srrm3* staining). Laser and detector settings: Exp 1 (*srrm3*): 639 nm, 4%, and gain 770 V (B1-Alexa647); Exp 2 (*elavl3*): 561 nm, 0.9%, and gain 700 V (B2-Alexa546); Exp 3 (*fosab* + *elavl3*): 639 nm, 3%, and gain 750 V (B1-Alexa647) and 561 nm, 2.2%, and gain 700 V (B2-Alexa546).

#### Image registration and visualization of srrm3-labeled brains (Exp 1)

Three-dimensional (3D) stacks were registered to the ZBB reference atlas using ANTs (v2.3.5) following (*104*). Outside-brain voxels were masked, and images were displayed as sagittal and dorsal maximum intensity projections in Fiji/ImageJ (v2.14.0/1.54f), using a Fire LUT.

#### Morphological analysis (Exp 2 and 3)

*elavl3*-labeled brains were registered to an in-house reference using the Computational Morphometry Toolkit (CMTK), following (*110*). Brain-volume comparisons were performed with MAPMapping (*110*) as described previously (*55*), using the default FDR = 0.00005 threshold.

#### Quantification and visualization of fosab staining (Exp 3)

*fosab*+*elavl3* stacks were registered to the ZBB reference with ANTs (v2.3.5), following (*104*). Background fluorescence was corrected in MATLAB by subtracting the pixel-intensity mode computed from voxels within a whole-brain mask as a robust estimate of baseline fluorescence. Negative values were set to zero. Stack-wise multi-Otsu thresholds (scikit-image in Python) for background estimation adapted from (*101*) yielded comparable results. For voxel-wise statistical comparisons, stacks were converted to 8-bit TIFF format, downsampled to 300 × 679 × 80 (*x*,*y*,*z*) and presmoothed with a 2D Gaussian filter (σ = 1). Pixel-wise intensity differences between genotypes were computed using MAPMapping (*110*). Significant ∆-median maps (FDR = 0.00005) were visualized in Fiji/ImageJ2 (v 2.14.0/1.54f) as sagittal and coronal maximum projections overlaid with Z-Brain outlines (*110*).

### Pharmacological assays

#### Behavioral pharmacology

Sleep-wake assays followed the procedures described in “Sleep-wake experiments and analysis.” At 5 dpf, larvae were plated using a P200 (tip cut) by gently tapping on the well edge, not transferring additional media, into alternating drug/control wells (premixed E3 + drug or matched control). Details on the drugs, concentrations, and preparation are in the Supplementary Materials. Drug/DMSO E3 mixes were refreshed every morning. For ropinirole and clonidine, activity change (%) relative to genotype-matched controls was calculated from percentage time active as: [(activity − mean activity of same-GT control) / |mean activity of same-GT control|] × 100. Dose-response curves (log_10_ concentrations) were fitted with a four-parameter logistic (4PL) model (lower asymptote fixed at 0) using drm from drc (v3.0-1) in R, and median effective dose (ED_50_) parameters were used as input for statistical testing (*t* test) with compParm. For SQ22536, forskolin, rolipram, and H-89 activity changes (%) in percentage time active were computed relative to WT controls and compared using Wilcoxon tests: [(activity − mean activity of WT control) / |mean activity of WT control|] × 100.

#### RNA-seq of pharmacologically treated larvae

Larvae were treated the afternoon before dissociation (mirroring treatment times for behavioral assays) with either drug + DMSO or to matched DMSO control (≤0.1%) (data S4). Concentrations were chosen to (i) reduce *srrm3*^∆eMIC^ daytime activity to WT levels (10 μM clonidine, 10 μM ropinirole, and 500 μM SQ22536) or (ii) elevate WT activity to *srrm3*^∆eMIC^ levels (0.75 μM rolipram and 7.5 μM forskolin). PTZ (1 mM) was used to increase neuronal activity without inducing seizures (*48*). Sample preparation, sorting, and sequencing followed “Bulk and single-cell RNA-seq.”

#### Survival assay

Larvae (4 to 6 dpf) were exposed to the mitochondrial calcium uniporter inhibitors ruthenium red (1.25 μM; R2751-1G, Sigma-Aldrich; stock = 1 mM in H_2_O) or Ru265 (4.7 μM; SML2991-5MG, Sigma-Aldrich; stock = 2 mg/ml in H_2_O) targeting the uniporter at higher specificity (*111*). Treatments were added directly to dishes containing mixed-genotype larvae (40 ml of E3). Survival was monitored hourly for 11 to 14 hours starting in the morning, with larvae alive at final time points included in Kaplan-Meier analyses. Genotyping was performed postexperiment (blinded) (*29*). Statistics in R used pairwise log-rank tests (survminer, v0.5.0).

### Western blot

At 5 dpf, larvae were divided into plates of equally numbered genotype-matched groups (*n* = 5 to 8 each) to minimize handling immediately before the experiment. On 6 dpf, larvae were anesthetized [5 min of tricaine (200 μg/ml)], enucleated, and heads collected on dry ice, briefly dropped into liquid nitrogen, and stored at −80°C. Tissue was lysed in radioimmunoprecipitation assay (RIPA) buffer [150 mM NaCl, 50 mM Tris-HCl, 1% Triton X-100, 0.5% sodium deoxycholate, 0.1% sodium dodecyl sulfate, and 1 mM EDTA] supplemented with protease inhibitors (Roche, catalog no. 11873580001) and PhosSTOP (Roche, 4906845001) using a Bioruptor Pico at 4°C (6 × 30-s on/off; Diagenode), shaken on ice (10 min, 350 rpm), and centrifuged (14,000 rpm, 15 min, 4°C). Protein concentration was measured in the supernatants by BCA assay (Thermo Fisher Scientific, 23227) and stored at −80°C. For Western blotting, samples were mixed with 4× loading buffer [200 mM Tris (pH 6.8), 400 mM dithiothreitol (DTT), 4% SDS, 0.2% bromophenol blue, and 20% glycerol] and denatured (95°C, 10 min), and 5 to 8 μg of protein was loaded per well on 10% Criterion TGX gels (5671035) and transferred to polyvinylidene difluoride (PVDF) membranes (Bio-Rad, 1620177). Total protein was visualized with DB71. Membranes were cut according to molecular weight to allow simultaneous detection of the protein of interest and vinculin from the same lane, blocked in 5% bovine serum albumin (BSA) in 1× TBS-T (0.1% Tween 20) for 45 min, and incubated with primary antibodies against pCREB, CREB, pPKA-C, and vinculin (Supplementary Materials). Horseradish peroxidase (HRP)–conjugated secondaries [goat anti-rabbit (Bio-Rad, 1706515), 1:10,000; sheep anti-mouse (Cytiva NA931V), 1:5000] were used. Signal was detected by enhanced chemiluminescence (ECL) and imaged on an iBright system (Invitrogen). Band intensities were quantified in Fiji/ImageJ from lane intensity profiles as the area under the curve (AUC) using a straight baseline drawn between flanking minima.

### Measuring cAMP levels

Larvae (5 to 10 per sample) were genotyped at 2 to 3 dpf and, when required, treated with rolipram/DMSO as appropriate and then snap frozen. Homogenization was performed in 170 μl of 0.1 M HCl using a handheld homogenizer with pestle followed by Bioruptor Pico sonication (4 × 20-s on/40-s off, 4°C). Lysates were spun down (13,000 rpm, 15 min, 4°C), and supernatants were used for cAMP ELISA (Enzo, ADI-900-066A). Following the manual, concentrations were estimated in R (drc::drm) using standards 1 to 5 and a 4PL model; samples with optical density outside the 4PL curve asymptotes were excluded, and full replicates were discarded if <2 samples per genotype remained. Of note, cAMP was measured across whole larvae; contributions from outside the brain (e.g., from the retina, heart, or muscles) cannot be excluded.

### ATAC sequencing

#### Sample preparation and sequencing

ATAC-seq was performed on 60,000 FACS-sorted *elavl3*:GFP^+^ cells per sample from enucleated 6 dpf larvae (two clutches; *n* = 11 to 12 larvae per sample). Nuclei isolation, lysis, and transposition followed published protocols (*72*, *112*). Libraries were prepared and sequenced at the CRG Genomics Facility (NovaSeq 6000; see data S4). See the Supplementary Materials for details.

#### ATAC-seq data processing and analysis

Reads were trimmed with trimmomatic (v0.39) (SLIDINGWINDOW:4:20; ILLUMINACLIP:AT_Tn5_adapters_NexteraPE-PE.fasta:2:30:10), aligned to danRer10 genome using bwa (v0.7.18-r1243-dirty), and deduplicated/sorted with bamsormadup from biobambam2 (v2.0.183) and samtools (v1.21), resulting in ∼ 50M paired-end reads per sample. Nucleosome-free fragments (1 to 120 bp) were extracted using alignmentSieve from deeptools (v3.5.5) after Tn5 offset correction. Consensus peaks were called with macs3 (v3.0.2) (narrow peaks, no model, *q* = 0.01) on merged.bam files. Read counts per peak were obtained using featureCounts (v2.0.8) (20% min overlap) including all fragment lengths, and peaks were annotated with annoPeaks from ChIPpeakAnno (v3.38.1). TF footprinting was performed with TOBIAS (v0.17.1) using PFAM/JASPAR motifs (746 vertebrate motifs), restricted to clutch 1 due to altered fragment length profiles in ∆eMIC2 (fragment length medians: WT1 = 103; ∆eMIC1 = 103; WT2 = 104; ∆eMIC2 = 262).

### Statistical analysis

All statistical analyses were performed in R (v4.4.0). Normality was assessed using the Kolmogorov-Smirnov test; log-transformations or nonparametric tests were applied when assumptions were not met. Unless stated otherwise, significance was defined as α = 0.05 with *P* values or significance symbols shown in the figures [not significant (n.s.) > 0.05; *P < 0.05; **P < 0.01; ***P < 0.001]. LMMs used Satterthwaite-approximated *t* tests and were fitted using lme4 (v1.1-36) with lmerTest for inference; statistics in the text are reported as estimate ± SE and backtransformed when percentage changes are shown. For Wilcoxon tests, *W*-statistics are provided; for Fisher’s exact tests, ORs are reported. Measures of central tendency are means ± SEM unless noted otherwise. Multiple-testing corrections (Bonferroni or FDR) were applied when appropriate. Boxplots follow the R ggplot2 (v3.5.1) default [median, first and third quartiles (box limits), and 1.5 × interquartile range (IQR) whiskers]. Detailed statistical models, sample sizes, and corrections are reported in Materials and Methods or figure legends. An overview of key software/algorithms/packages used for analysis can be found in table S1.

## Supplementary Materials

### The PDF file includes:

Supplementary Text Figs. S1 to S9 Table S1 Legends for data S1 to S6 References
