# Supplementary material for: Neuronal microexons modulate arousal via the cAMP-PKA-CREB pathway in zebrafish
Source: Sci Adv. 2026 Jun 19;12(25):eady8291. doi: 10.1126/sciadv.ady8291 (PMC13281805; doi:10.1126/sciadv.ady8291)
Supplement: Supplementary file 1 — Supplementary Text Figs. S1 to S9 Table S1 Legends for data S1 to S6 References [file sciadv.ady8291_sm.pdf]

Science Advances
AAAS

Supplementary Materials for

Neuronal microexons modulate arousal via the cAMP-PKA-CREB pathway in zebrafish

Tahnee Mackensen *et al.*

Corresponding author: Tahnee Mackensen, tahnee.mackensen@gmail.com; Manuel Irimia, mirimia@gmail.com

Sci. Adv. **12**, eady8291 (2026)
DOI: 10.1126/sciadv.ady8291

The PDF file includes:

Supplementary Text
Figs. S1 to S9
Table S1
Legends for data S1 to S6
References

Other Supplementary Material for this manuscript includes the following:

Data S1 to S6

Supplementary Text

Extended Materials and Methods

Zebrafish lines

Microexon mutant lines (gene name and exon *VastID* from Vast database [*VastDB*]: *madd* (DreEX0044304), *shank3a* (DreEX0066086), *vav2* (DreEX0084167), *vti1a* (DreEX0084665); and master-regulator mutants were generated in-house by CRISPR/Cas9 (*29*, *37*) in a *Tg(elavl3:GFP)* background (*114*) and maintained as heterozygous. A second *srrm3* founder (*crg5*, ZFIN: ZDB-ALT-230328-7) with a 19 bp deletion was used for phenotype validation. The $srrm3^{crg3/crg3}$;$srrm4^{crg4/crg4}$ double mutant (ZFIN: ZDB-FISH-230512-7) was generated by intercrossing $srrm3^{+/crg3}$ and $srrm4^{+/crg4}$ (ZFIN: ZDB-ALT-230328-6). For behavioural controls, $vsx1^{+/-}$;$vsx2^{-/-}$ larvae (*38*) at 48 hours post fertilization (hpf) were kindly provided by the Martínez-Morales lab at the Centro Andaluz de Biologia del Desarrollo (CSIC/UPO/JA), Sevilla. For imaging (hybridization chain reaction [HCR], two-photon [2P]), the *mitfaw2* nacre background (*115*) was crossed into $srrm3^{+/\Delta eMIC}$ background to reduce pigmentation. 2P experiments used *Tg(elavl3:H2B-GCaMP6s)*^{jf5} (*50*) crossed into *nacre*;$srrm3^{+/\Delta eMIC}$. The larvae were maintained heterozygous for $srrm3^{\Delta eMIC}$ and *Tg(elavl3:H2B-GCaMP6s)*^{jf5}, screened for fluorescence, and genotyped at 3 days post fertilization (dpf) (*29*, *100*).

High-resolution behavioural tracking: Set-up, data pre-processing and swim bout categorization

The tracking setup was described previously (*35*, *45*). In brief, larvae were tracked in an acrylic arena with a rounded edge (radius of curvature: 11 mm, diameter: 10 cm, depth: 4 mm) and illuminated from below using an infrared light. The arena was sheltered from outside lighting. The larvae's eyes and tail (nine equally spaced segments of 300 µm) were tracked online at 700 Hz throughout the experiment via custom C# software and swim bouts were detected as previously described (*35*). Data was pre-processed in *MATLAB* as previously described (*35*, *45*), obtaining the following output files: "bouts.mat" with information on bout category, bout kinematics and PC coordinates; "camlog.mat" with frame-by-frame information on the tail tracking angle across segments; "stimlog.mat" with information on light/dark stimuli. Relevant information was extracted in *Python* and summarised into data frames, one with bout data, analysed in R and one with tail angle data analysed and visualised using *Python*. Bout categorization was carried out by assigning each bout to 1 of 13 bout types based on k nearest neighbours (k = 50) analysis using an existing balanced data set of labeled examples of each bout type (*35*, *45*). Each bout type was described by 73 kinematic parameters and embedded into a previously computed PC-space (*35*) based on a large set of behavioural data under various stimulus conditions. This space and centre of mass (CoM) of bout clusters were used for the principal component analysis (PCA) to visualise bout distributions and to calculate Euclidean distances to the CoM for each bout category in *MATLAB*. Euclidean distances were compared using Wilcoxon-test and p-values FDR adjusted for the number of bout categories tested (Data S2).

High-resolution behavioural tracking: Statistics for long bout kinematic comparisons

Statistical significance was obtained by permutation testing: for each kinematic parameter tested, 1,000 equally sized random bout sets (matched by genotype) were generated, and the p-value computed as $(r+1)/(n+1)$, where r is the number of permutations more extreme than the observed value (*116*, *117*). For example, if the long bouts have a mean absolute bout

angle of 67 degrees and out of n = 1000 randomly sampled sets the mean bout angle was found to be higher for r = 5 permutations, then the probability of observing, a mean absolute bout angle of > 67 degrees, by chance would be estimated at *(5+1)/(1000+1) = 0.006*.

2P Calcium imaging: Set-up and experimental design
For functional imaging the sample was illuminated at 920 nm with a laser power of ~ 11.2 mW and for anatomical stacks at 800 nm with a laser power of ~ 9.1 mW. For clutch 1 (C1) images were taken at a resolution of 500 x 469 pixels with pixel size of 0.7 µm. For C2 and C3 image resolution was 693 x 368 pixels with a pixel size of 0.61 µm to image more of the pallium and hindbrain (Fig. 3D; Fig. S3B). The field of view (FOV) includes parts of the pallium, habenula, pretectum, optic tectum, tegmentum and anterior hindbrain. Acquisitions were made at 3.6 Hz for 3-4 planes per larva with 10 µm z-spacing, scanned one plane at a time. Visual stimuli were designed and presented as described in (*104*) in the order of 30 x grey screen epochs (~ 30 sec; 108 frames) followed by a pseudo-random presentation of 4 different stimuli every 30 sec, including 5 x whole-field light (~ 3 sec; 12 frames), 10 x whole field dark (~ 3 sec; 12 frames), 5 x looming dot (~ 6 sec; 21 frames), 5 x overall dimming as control for the looming dot (~ 6 sec; 21 frames) (*118*).

Sequencing experiments: Dissociation and FACS-sorting
Following (*29*), after a wash in Neurobasal^{TM} (NB) media, larvae were suspended in 350 µl NB + supplements (1% Penicillin/Streptomycin, 1% N2, 1% L-glutamine, 2% B-27) with 125-150 µl 0.5% trypsin-EDTA and subjected to three rounds of mechanical trituration (5, 4, and 3 min). Trypsinization was stopped with 800 µl NB + supplements + 2% fetal bovine serum (FBS), and cells were pelleted (4 min, 5000 rpm). Pellets were resuspended in NB + 2% FBS for bulk RNA-sequencing (RNA-seq) or in 1 x distilled phosphate-buffered saline (PBS) for single-cell RNA-seq, filtered through a 40 µm mesh (Cultek - 88141378), and kept on ice until FACS. For FACS, cells from *srrm3*$^{\Delta eMIC}$ and WT siblings with *Tg(elavl3:GFP)* background were sorted to isolate predominantly post-mitotic neurons. Propidium iodide (1:1000) was added to exclude dead cells. Sorting was performed on a BD Influx (488 nm excitation, 100 µm nozzle, 1.5-drop pure mode). Gates were set to remove debris, aggregates, and PI-positive cells following (*29*), and up to ~500,000 GFP$^+$ cells were collected per sample in NB + supplements. Cell viability averaged ~75% (Data S4). For single-cell RNA-seq, GFP$^+$ cells were sorted into 96-well plates containing 3.7 µl of 0.378% BSA in 1 x PBS for a final concentration of 0.04% BSA to minimise adhesion and aggregation and then transferred to low-binding tubes (525-0130, VWR) for downstream processing.

Differential gene expression analysis: Sequence similarity
For all *srrm3*-dependent microexon genes (ΔPSI < –15; ≤51 nt), we computed pairwise percentage sequence similarity in R with their paralogs (background set) and differentially expressed paralogs (target set), excluding those harbouring *srrm3*-regulated microexons themselves. A single target/background gene could have multiple similarity scores if it was a paralog of multiple *srrm3*-regulated microexon harbouring genes. To test whether differentially expressed paralogs of mis-spliced genes were more similar than expected, we compared similarity scores of the target set to the background distribution using a permutation test (n = 1,000 permutations); $p = (r+1)/(n+1)$, where r is the number of permutations more extreme than the observed value (*116*, *117*).

HCR: Experimental procedure
In brief, larvae were stepwise dehydrated from methanol and then washed 3 x in PTw (1 x PBS, 0.1 % Tween 20). Larvae were permeabilized and then pre-hybridized in probe

hybridization buffer (*Molecular Instruments*) at 37 °C for 30 min. This pre-hybridization solution was removed and a probe hybridization buffer with 0.8 pmol of probes was added for incubation over night (ON) at 37 °C. Larvae were washed in the probe wash buffer (*Molecular Instruments*), followed by washes with 5 x SCCT (sodium chloride sodium citrate, 0.1% Tween 20). Samples were then pre-amplified in 1 ml of amplification buffer (*Molecular Instruments*) for 30 min at room temperature (RT). Meanwhile 2 µl (3 µM stock) of each hairpin h1 and h2 of each amplifier (B1, B2) was added to the amplification buffer and heated for 90 sec at 95 °C before cooled down to RT in the dark. The pre-amplification solution was removed, the hairpin solution added and the larvae incubated in the dark ON at RT. Larvae were repeatedly washed in 5x SCCT and transferred to 1 x PBS. DAPI was added as a nuclear stain for ~ 1h at 1.0µg/mL. Samples were stored at 4 °C in 1 x PBS for up to 4 days before imaging.

Behavioural pharmacology: Drug preparation
Forskolin (powder), and ropinirole (powder) were dissolved at 10 mM in dimethyl sulfoxide (DMSO) while rolipram and H-89 were purchased at 10 mM in DMSO (ropinirole hydrochloride: R2530-100MG, Sigma; forskolin: F6886-10MG, Sigma; rolipram: HY-16900, MedChemExpress; H-89 dihydrochloride: HY-15979A, MedChemExpress). All four were diluted to stocks of 1 mM in 10% DMSO/H_2O. Clonidine (powder) was dissolved at 10 mM in H_2O and stored as 1mM in H_2O stock (Clonidine hydrochloride: C7897-25MG, Sigma). SQ22536 (powder) was dissolved at 10 mM in 1 x PBS stock (SQ22536: HY-100396, [25 mg], MedChemExpress). For each experiment the control concentration of % DMSO, H_2O or 1 x PBS were kept equal to that of the treatment condition. For example, 5µM of rolipram from a 10% DMSO stock would result in 0.05% of DMSO. The maximum % DMSO was 0.2% for 20µM of ropinirole treatment. Concentration ranges were based around concentrations of published zebrafish work: starting with 2.5µM for clonidine, ropinirole (*119*) and H-89 (*69*), with 5µM for forskolin, rolipram (*69*) and SQ22536. SQ22536 concentrations were increased up to 500 µM (*120*) due to limited behavioural effects at lower doses.

Western blotting: List of primary antibodies
anti-CREB-Ser133 (Cell Signaling 9198S, 1:1000)
anti-CREB (Sigma MAB5432, 1:500)
anti-PKA-C-Thr197 (Cell Signaling #5661, 1:1000)
anti-Vinculin (Abcam ab129002, 1:1000)

ATAC-seq: Sample preparation and sequencing
Cells were pelleted (120 g, 5 min) and following (*72*) resuspended for lysis in 600 µl of nuclear extraction buffer (250 mM sucrose, 25 mM KCl, 5 mM MgCl2, 20 mM HEPES-KOH [pH 7.8], 0.5% IGEPAL CA-630, 0.2 mM spermidine, 0.5 mM spermidine, nuclease-free water), and homogenized on ice using a Dounce homogenizer (Sigma). Following (*112*), nuclei were isolated (1000 g, 7 min) and tagmented in 50 µl Nextera transposase mix (25 µl 2x TD buffer, 3.5 µl TN5, 21.5 µl H_2O; 20034210) for 30 min at 37 °C and 400 rpm. DNA was purified (Qiagen MinElute, 28004). Libraries were prepared at the CRG Genomics facility using NEBNext Q5 Hot Start HiFi Master Mix (New England Biolabs, M0543L) and dual-index Nextera primers (12-14 PCR cycles) at a final concentration of 1.25 µM, purified with AgenCourt AMPure XP beads (Beckman Coulter, A63882), QC-checked by Bioanalyzer/Fragment Analyzer (5067-4626; DNF-474), quantified by qPCR using the KAPA Library Quantification Kit KK4835 (Roche, 07960204001), and sequenced (2×51+10+10 bp) on an Illumina NovaSeq 6000 (~67–70 M reads/sample) (Data S4).

Fig. S1.

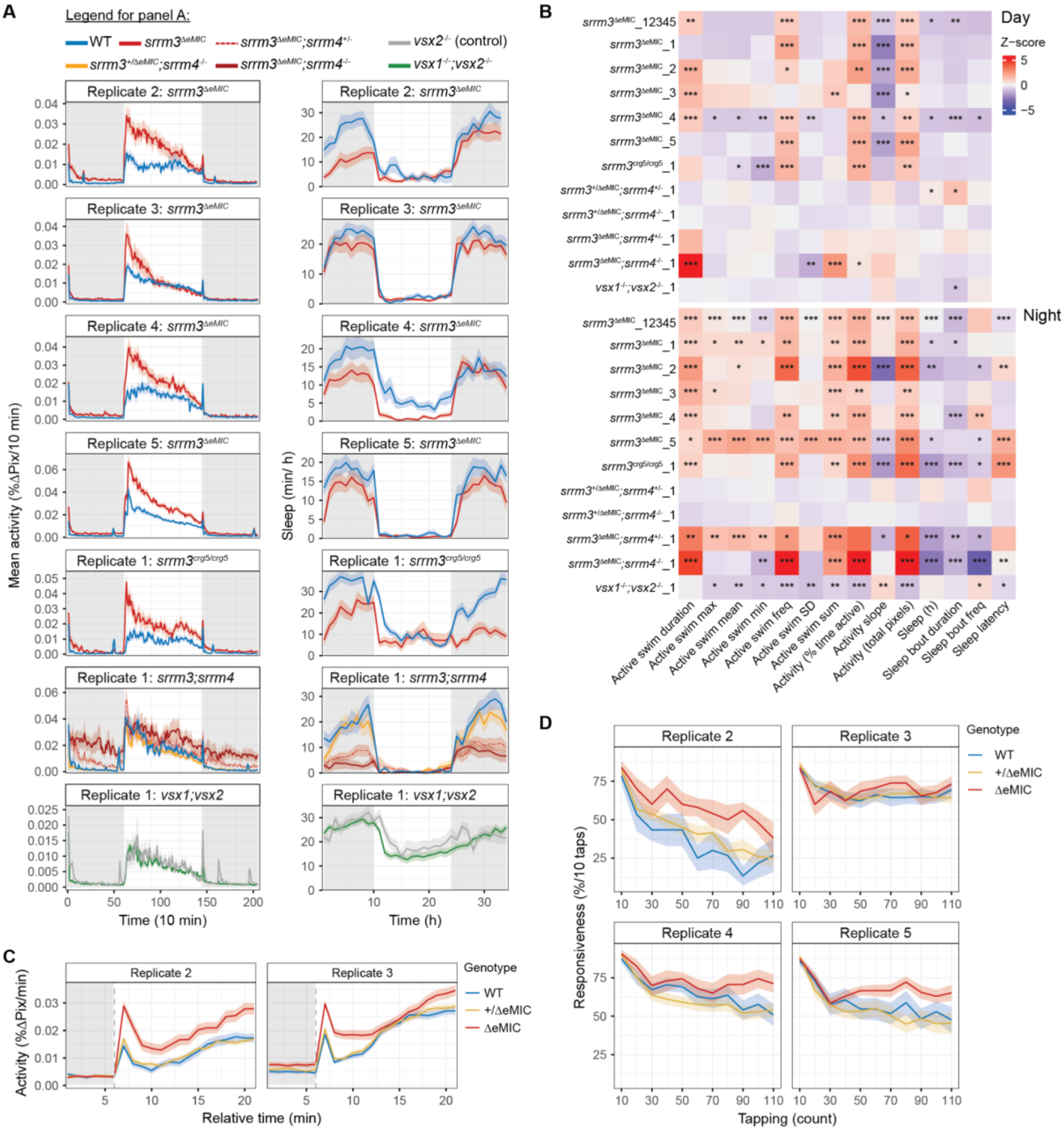

Supplementary Fig. 1. ***srrm3***$^{\Delta\mathbf{eMIC}}$ **larvae exhibit hyperactivity, sleep loss, sensory hypersensitivity, and excess thigmotaxis.**

(A) Representative traces (mean ± SEM) of locomotor activity (%ΔPix/10 min; left) and sleep (min/h; right) recorded over 74 h (5-8 dpf) under a 14 h light / 10 h dark cycle (white and grey backgrounds, respectively). For visual clarity, heterozygous traces are omitted except for the *srrm3*$^{\Delta eMIC}$;*srrm4*$^{+/-}$ and *srrm3*$^{+/\Delta eMIC}$;*srrm4*$^{-/-}$ double mutants, as heterozygotes closely resemble WT. n = 10-43 larvae per condition.

(B) Summary heatmap of behavioural parameters quantified during daytime (6 dpf) and nighttime (5-6 dpf and 6-7 dpf). Values are shown as z-scores averaged across larvae and normalized to WT or control (*vsx2*$^{-/-}$) siblings. Numbers following genotype labels (1-5) denote biological replicates; “12345” indicates averages across all five clutches used for linear mixed effects model (LMM) statistics.

(C) Light-on response at 6 dpf. Traces show mean ± SEM activity (%ΔPix/min), averaged across five transitions per larva and plotted by clutch and genotype. N = 2 clutches, 12-45 larvae per genotype and clutch.
(D) Habituation to repeated mechanical tapping at 6 dpf. Traces show mean ± SEM responsiveness (% responding per 10 taps) during 10 stimuli at 90 s inter-stimulus intervals followed by 100 stimuli at 5 s intervals. N = 4 clutches, 5-46 larvae per genotype and clutch. Biological replicates in panels **A, C, D** are numbered starting from replicate 2, as replicate 1 is shown in Fig. 1. Detailed statistics and sample sizes across panels are provided in Data S1.

Fig. S2.

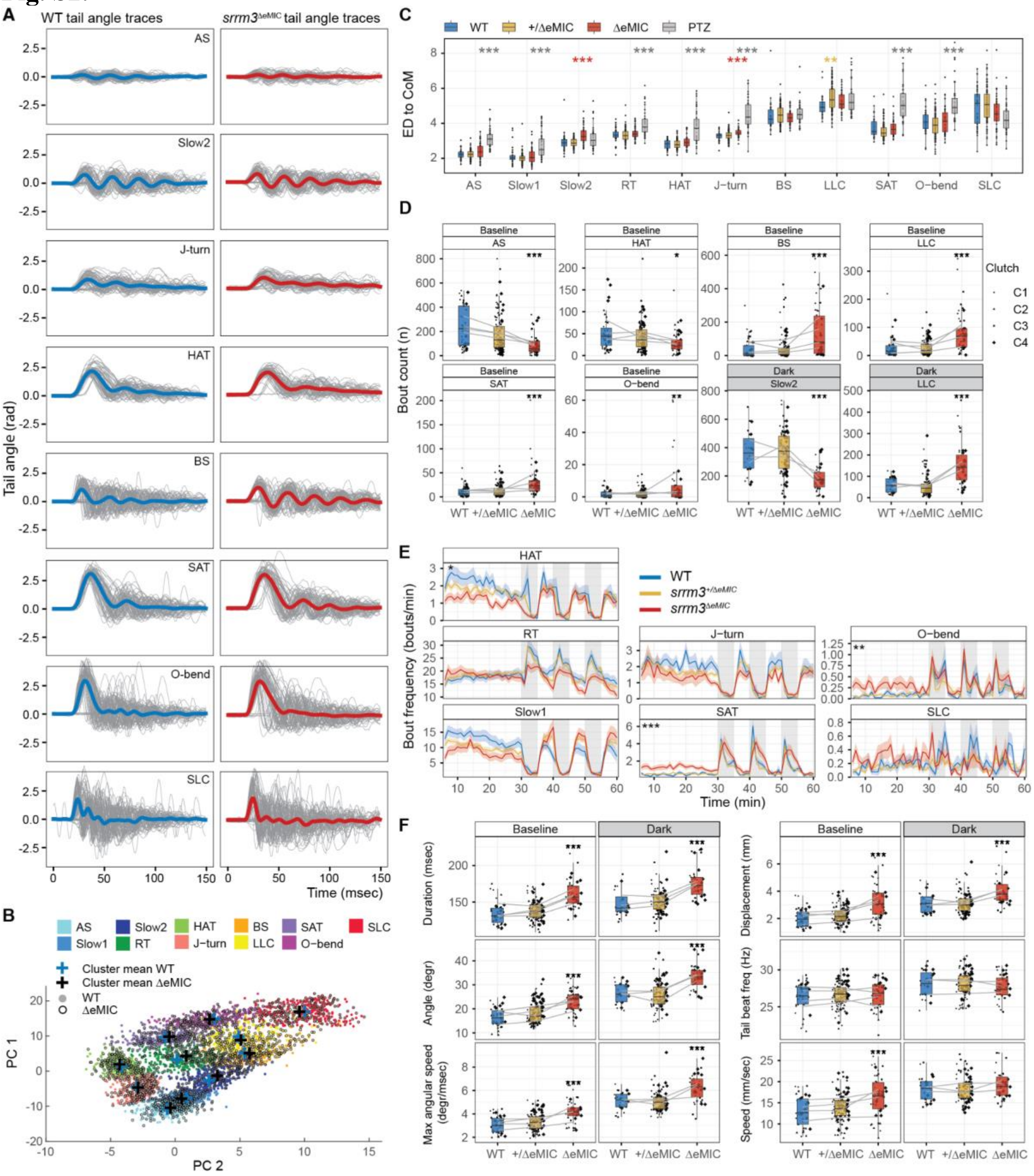

Supplementary Fig. 2. Bout type usage and kinematics indicate elevated arousal in *srrm3*$^{\Delta eMIC}$ larvae.

(A) Representative tail-angle traces (7th tail segment) for eight bout types (remaining types in Fig. 2B). Grey lines show 70 randomly sampled bouts per type; bold lines indicate the mean. Data are from one clutch (8 WT, 14 *srrm3*$^{\Delta eMIC}$ larvae).

(B) Clustering of swim bouts in principal component (PC) space based on kinematic features (*35*). Points represent individual bouts (200 randomly sampled per bout type); crosses indicate genotype-specific cluster means (WT: blue, n = 41 larvae; *srrm3*$^{\Delta eMIC}$: black, n = 46 larvae).

(C) Median Euclidean distance (ED) of each larva's bouts to the cluster CoM for each bout type. Dots represent larvae. Statistics by Wilcoxon test; star colour indicates comparison relative to WT. P-values were FDR-corrected across bout types. Y-axis limits are set for

visualization (1.5-8.5). Sample sizes: WT (n = 41), *srrm3*$^{+/\Delta eMIC}$ (n = 110), *srrm3*$^{\Delta eMIC}$ (n = 46), 10 mM PTZ-treated (n = 43; mixed genotypes).
(D) Bout counts for bout types significantly different between WT and *srrm3*$^{\Delta eMIC}$ siblings, shown separately for baseline and dark conditions.
(E) Mean bout frequency over time (bouts/min) (remaining types in Fig. 2D). Grey shading indicates dark periods. Traces show mean ± SEM.
(D,E) Statistics by negative binomial generalized LMM (GLMM); p-values Bonferroni-corrected across bout types within each condition (baseline or dark). Baseline: N = 4 clutches; dark: N = 3 clutches; 7-33 larvae per genotype and clutch.
(F) Mean kinematic parameters by bout type and condition (baseline, dark). Statistics by LMM with Satterthwaite-approximated *t*-tests and Bonferroni correction across bout types within each condition (baseline or dark).
Dots in **D,F** represent individual larvae (shaped by clutch); grey lines connect clutch means. Detailed statistics and sample sizes are provided in Data S2.
Bout abbreviations: AS, approach swim; RT, routine turn; HAT, high-angle turn; BS, burst swim; SAT, spot avoidance turn; SLC, short-latency C-start; LLC, long-latency C-start.

Fig. S3.

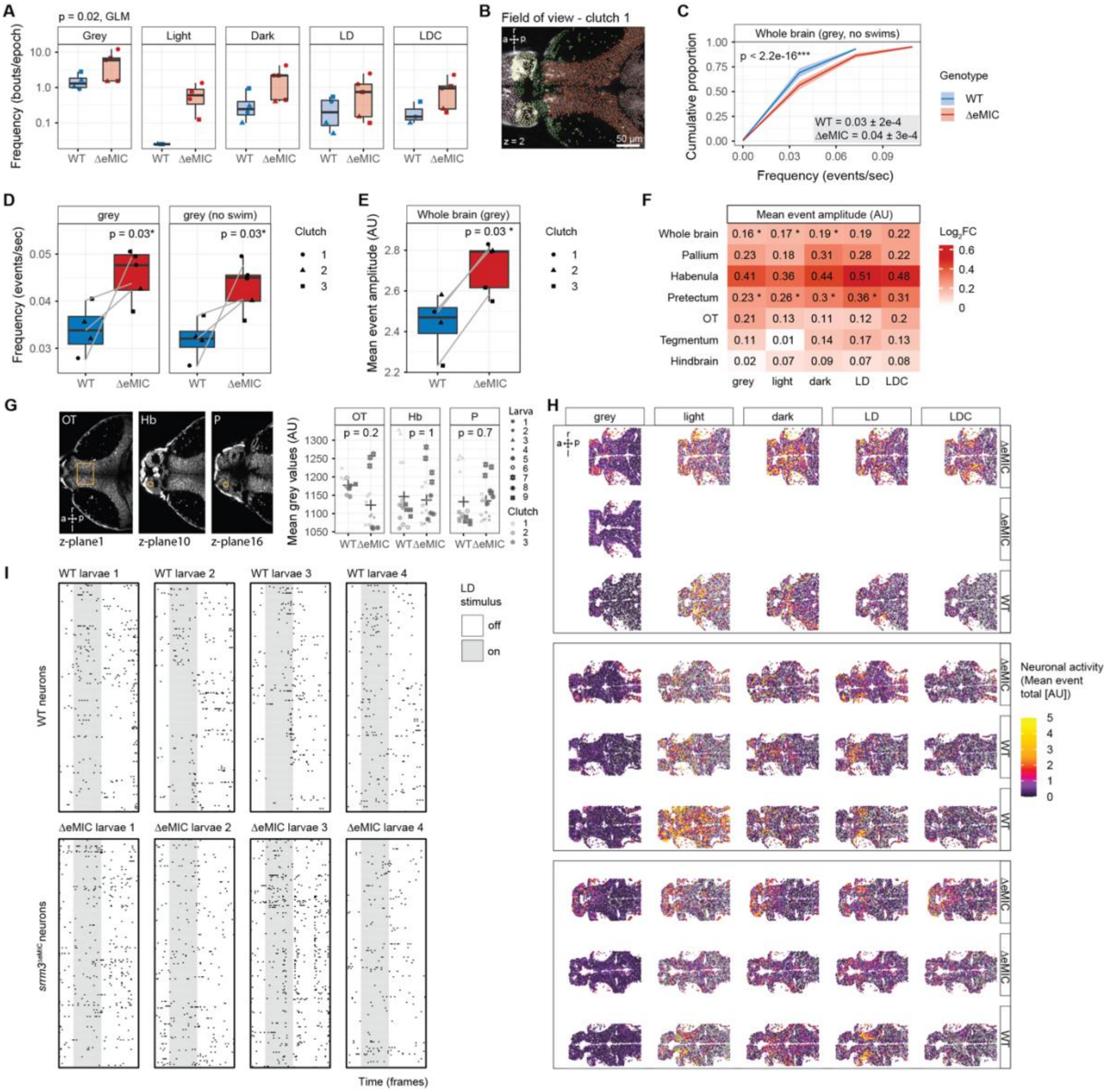

Supplementary Fig. 3. ***srrm3*$^{\Delta eMIC}$ larvae show elevated baseline neuronal activity and altered responses to visual stimuli.**

(A) Swim bout frequency (bouts/epoch; $\log_{10}$ scale) across visual stimuli. Dots represent individual larvae (shaped by clutch). Statistics by GLM controlling for stimulus and larva (frequency: 1.3 ± 0.6 bouts/epoch; p = 0.02). n = 4 WT and 5 *srrm3*$^{\Delta eMIC}$ larvae from 3 clutches.

(B) Representative FOV for clutch 1 showing one z-plane with regions of interest (ROIs) coloured by Zebrafish Brain Browser (ZBB) anatomical region. Pink, pallium; yellow, habenula; red, optic tectum; brown, hindbrain; green, others.

(C) Empirical cumulative distribution functions (ECDFs) of event frequency for the whole brain at baseline, excluding swimming periods. Lines show mean ± SEM and are truncated at y = 0.97. Insets show mean ± SEM across neurons. Statistics by Wilcoxon test comparing n = 36,951 WT and 38,940 srrm3$^{\Delta eMIC}$ neurons from 4 WT and 5 *srrm3*$^{\Delta eMIC}$ larvae.

(D) Event frequency at baseline across the whole brain, including all frames (left) or excluding swim frames (right).

(E) Mean event amplitude at baseline across the whole brain.

(D,E) Dots represent larval means; lines connect clutch means. Statistics by Wilcoxon test (n = 4-5 larvae/genotype; 3 clutches).
(F) Heatmap of mean event amplitude $\log_2$ fold-change ($\log_2$FC) (WT vs srrm3$^{\Delta eMIC}$). Blue indicates higher amplitudes in WT, red higher in *srrm3*$^{\Delta eMIC}$. Statistics by Wilcoxon test (n = 4-5 larvae/genotype; 3 clutches).
(G) GCaMP6s baseline fluorescence measured at 800 nm (activity-independent) at constant laser power and gain settings across larvae. Left: representative anatomies showing ROIs (orange) in optic tectum (OT), habenula (Hb), and pallium (P). Right: mean intensity per larva and region. Statistics by Wilcoxon test (n = 4-5 larvae/genotype; 3-4 measurements/region; 3 clutches).
(H) ZBB-registered anatomy plots for one z-plane showing mean event total (AU) across all stimuli and larvae. Values >5 were clipped for visualization. Boxes indicate larvae from the same clutch.
(I) Raster plots of inferred events from 243 habenula neurons across 4 larvae per genotype during the first well-aligned looming-dot (LD) epoch (60 frames, ~15 s). Grey shading indicates stimulus period.
Orientation labels in **B,G,H**: a, anterior; p, posterior; r, right; l, left.

Fig. S4.

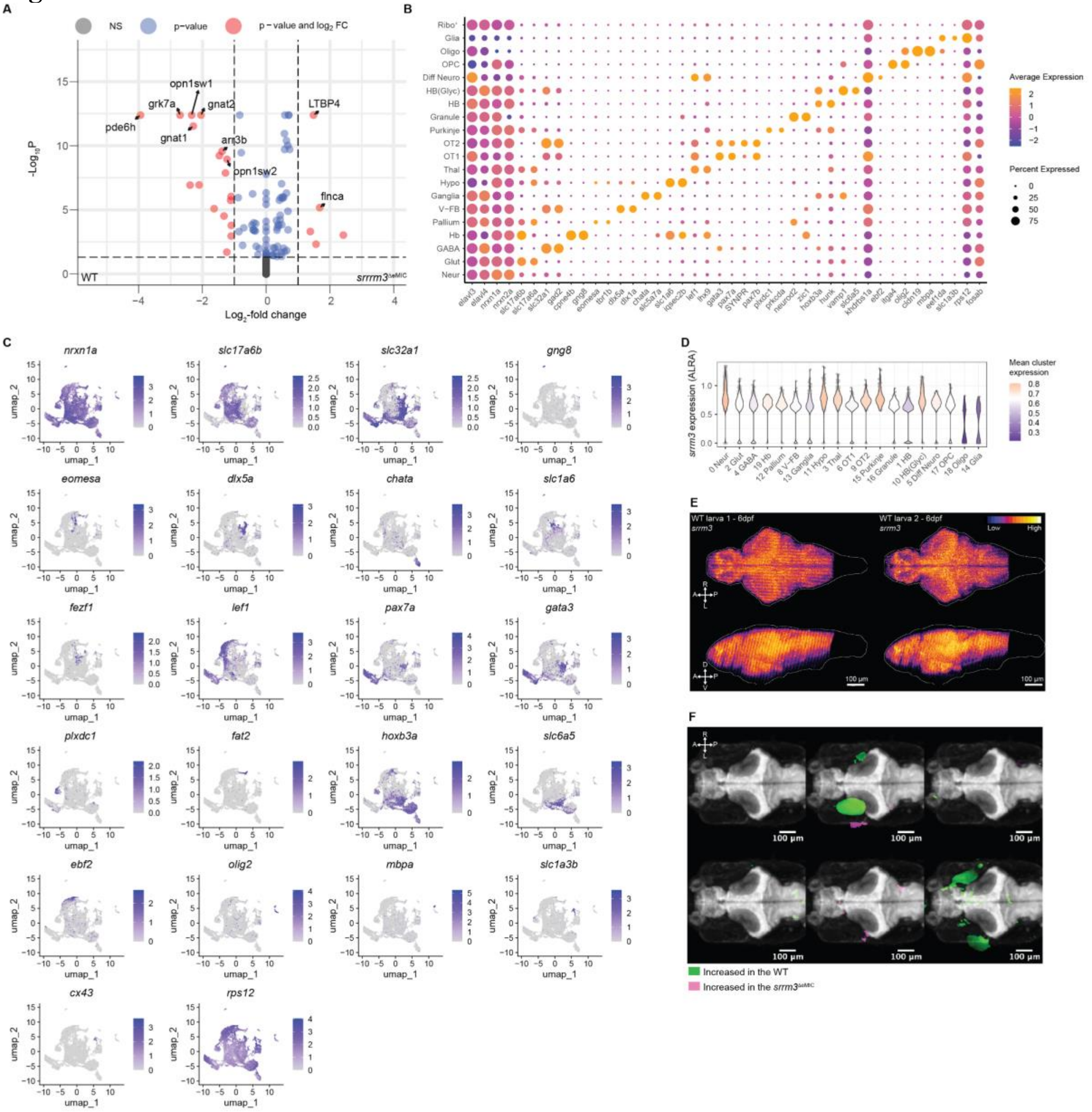

Supplementary Fig. 4. *srrm3*$^{\Delta eMIC}$ larvae show reduced microexon inclusion without changes in cell-type composition.

(A) Volcano plot of the 8,000 most differentially expressed genes (DEGs; ranked by log$_2$FC) comparing *srrm3*$^{\Delta eMIC}$ and WT neurons from non-enucleated 5 dpf larvae (Data S6). Cut-offs for visualisation: p.adj < 0.05 and |log$_2$FC| > 1. Upregulated genes are shown on the right, downregulated on the left. N = 2 biological replicates per genotype (20-26 larvae/replicate).

(B) Dot plot showing two representative marker genes per single-cell cluster (Data S6). Markers were expressed in >20% of cells with mean log$_2$FC > 0.25 versus all other clusters (Wilcoxon rank-sum test). N = 2 biological replicates per genotype (13-21 larvae/replicate).

(C) UMAP feature plots of canonical marker genes defining major neuronal and glial populations (see panel labels). Colour indicates log-transformed, batch-corrected expression counts. Neuronal: *nrxn1a*$^+$; GABAergic: *slc32a1*$^+$; Glutamatergic: *slc17a6b*$^+$; Habenula: *gng8*$^+$; Pallium: *eomesa*$^+$; Subpallium/hypothalamus/preoptic: *dlx5a*$^+$; Ganglia (acetylcholine$^+$): *chata*$^+$; Hypothalamus: *slc1a6*$^+$ (glutamatergic), *fezf1*$^+$ (GABAergic); Dorsal diencephalon (thalamus): lef1$^+$; OT1: *pax7a*$^+$; OT2: *gata3*$^+$; Purkinje: *plxdc1*$^+$; Cerebellum (granule): *fat2*$^+$; Hindbrain: *hoxb3a*$^+$; Hindbrain (glycinergic): *slc6a5*$^+$; Differentiating

midbrain: *ebf2*$^+$; Oligodendrocytes: *olig2*$^+$; OPCs: *mbpa*$^+$; Glia: *slc1a3b*$^+$, *cx43*$^+$; Ribosomal: *rps12*$^+$.
(D) Violin plot of *srrm3* expression across WT clusters. Colours indicate relative cluster-average expression (purple, below average; white, average; orange, above average). Expression values per cell (dots) were *ALRA*-imputed for sparse single-cell matrices (Table S1).
(E) Maximum-intensity projections of whole-mount HCR for *srrm3* mRNA in two WT larvae at 6 dpf, shown in dorsal (top) and sagittal (bottom) views. Warmer colours indicate higher transcript abundance.
(F) Structural brain differences identified by MAPMapping overlaid onto an *elavl3* in-house reference brain. Colours denote significant regional delta means (green, higher in WT; magenta, higher in *srrm3*$^{\Delta eMIC}$). N = 6 clutches (8-11 larvae per genotype).
Orientation labels in **E,F**: A, anterior; P, posterior; R, right; L, left; D, dorsal; V, ventral.
Cluster names for **B,D** provided in Materials and Methods.

Fig. S5.

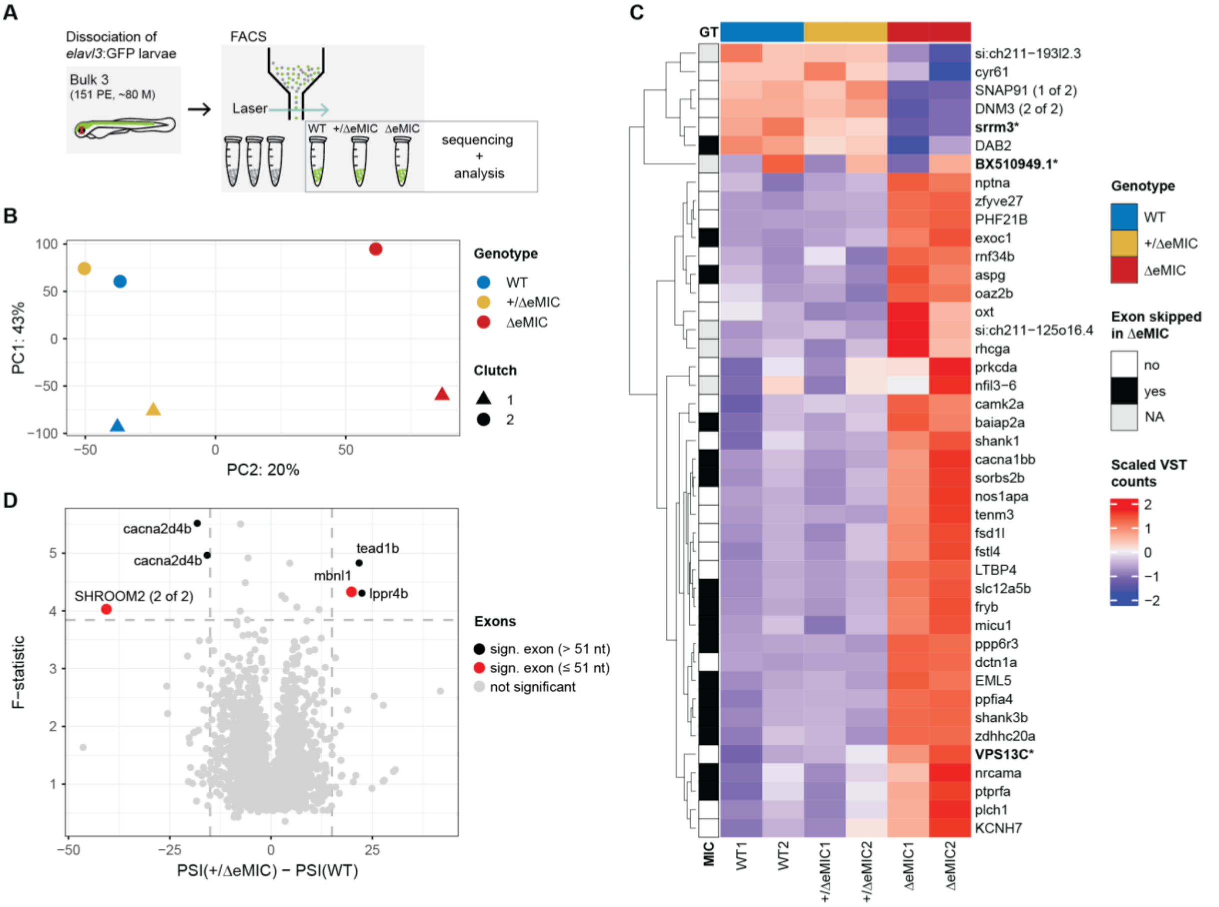

Supplementary Fig. 5. *srrm3*$^{+/\Delta eMIC}$ larvae show splicing and transcriptional profiles largely resembling WT.

(A) Experimental schematic for bulk RNA-seq 3 from FACS-sorted *elavl3*:GFP^{+} cells of WT, *srrm3*$^{+/\Delta eMIC}$, and *srrm3*$^{\Delta eMIC}$ larvae. N = 2 biological replicates with n = 11-15 larvae per sample.

(B) PCA of variance-stabilized gene counts. Each point represents one sample, shaped by clutch and coloured by genotype.

(C) Heatmap of genes differentially expressed between WT and *srrm3*$^{\Delta eMIC}$ (p.adj < 0.05, $|\log_2\text{FC}| > 0.5$), displayed as row-scaled variance-stabilized counts. Top annotation indicates genotype (GT). Left annotation denotes whether the gene harbours a microexon skipped in *srrm3*$^{\Delta eMIC}$ (ΔPSI < -15, black), no skipped exon (white), or no detected splice event (grey). Gene names in bold with asterisks indicate genes also differentially expressed between WT and *srrm3*$^{+/\Delta eMIC}$ (p.adj < 0.05).

(D) Volcano plot of differential splicing in WT vs *srrm3*$^{+/\Delta eMIC}$ (ΔPSI vs F-statistic). Significantly differentially spliced exons (F > 3.8, $|\Delta\text{PSI}| > 15$) are highlighted: microexons (≤51 nt, red), longer exons (>51 nt, black), and non-significant events (grey). Dashed lines indicate significance thresholds.

Fig. S6.

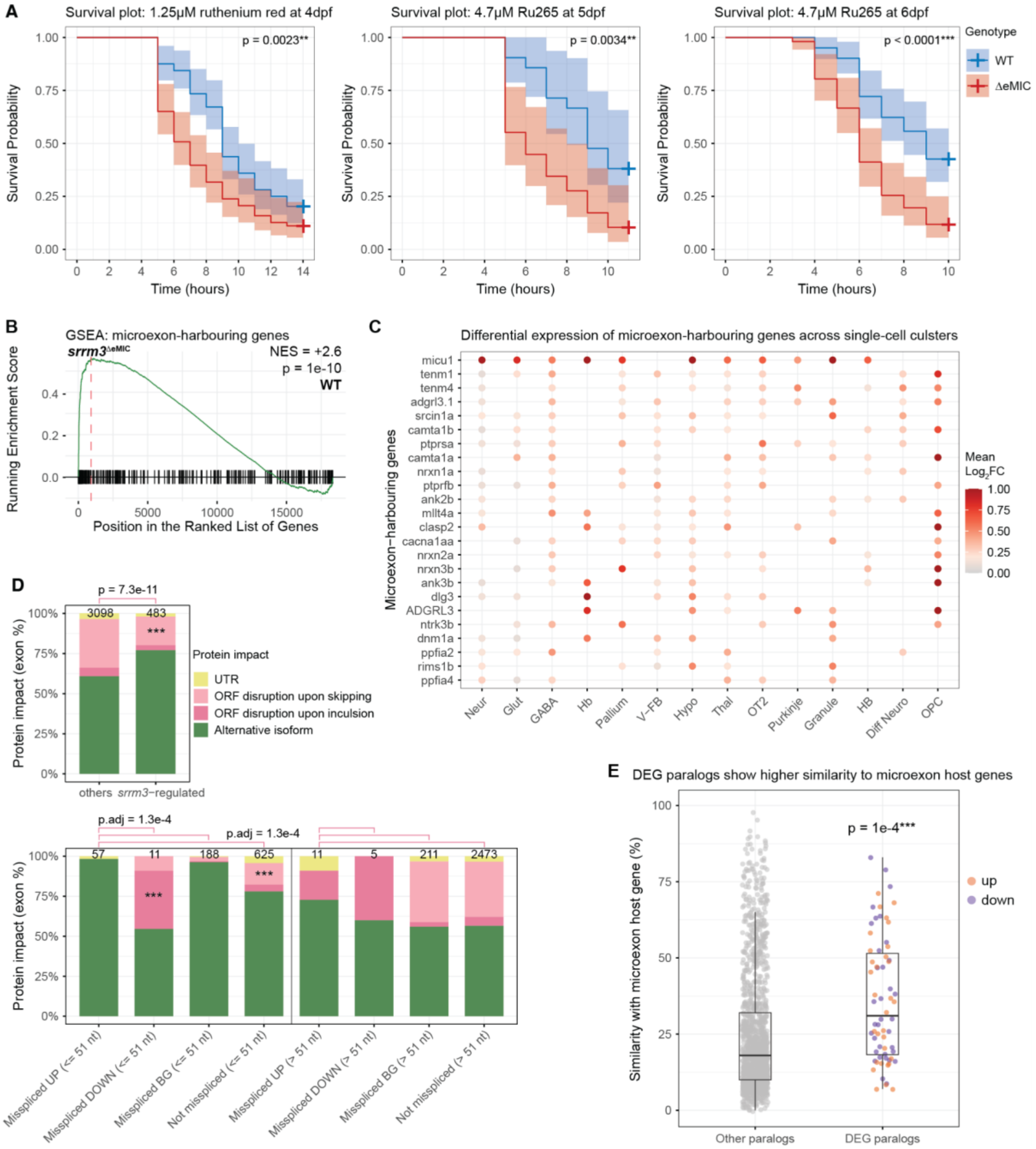

Supplementary Fig. 6. Genes upregulated in *srrm3*$^{\Delta eMIC}$ disproportionately harbour mis-spliced microexons.

(A) Kaplan-Meier survival curves following inhibition of the mitochondrial calcium uniporter. Left: 1.25 μM ruthenium red at 4 dpf (N = 4 clutches, 11-19 larvae/genotype); centre: 4.7 μM Ru265 at 5 dpf (N = 2 clutches, 6-30 larvae/genotype); right: 4.7 μM Ru265 at 6 dpf (N = 4 clutches, 8-20 larvae/genotype). Lines show survival probability ±95% confidence interval (CI) per genotype and clutch. Statistics by log-rank (Mantel-Cox) test.

(B) Gene set enrichment analysis (GSEA) of microexon-harbouring genes (≤51 nt, ΔPSI < -15) ranked by log_2FC between WT and *srrm3*$^{\Delta eMIC}$.

(C) *srrm3*-regulated microexon-harbouring genes contributing to the GSEA core enrichment in > 5 clusters, shown across single-cell clusters and coloured by mean log_2FC (values capped at 1 for visualisation). Full cluster names in Materials and Methods.

(D) Predicted protein impact of alternative exon inclusion or skipping. Top: comparison of exon proportion disrupting the open reading frame (ORF) for *srrm3*-regulated (|ΔPSI| > 15) vs all other exons. Bottom: analysis stratified by mis-splicing status (|ΔPSI| > 15), transcript regulation (up/down), and exon length (≤51 nt or >51 nt). Asterisks and p-values shown in case of significant differences in the proportion of exons disrupting the ORF for "Misspliced UP" vs any other same-length category. Statistical comparisons indicated by pink lines. Statistics by two-sided Fisher's exact tests with Bonferroni correction. Total exon counts indicated above bars. UTR, untranslated region.

(E) Paralogous genes that are differentially expressed (up- or downregulated) show greater sequence similarity to genes harbouring *srrm3*-regulated microexons (≤ 51 nt, ΔPSI < -15) than all other paralogs (permutation test). Each dot represents one paralog pair.

Fig. S7.

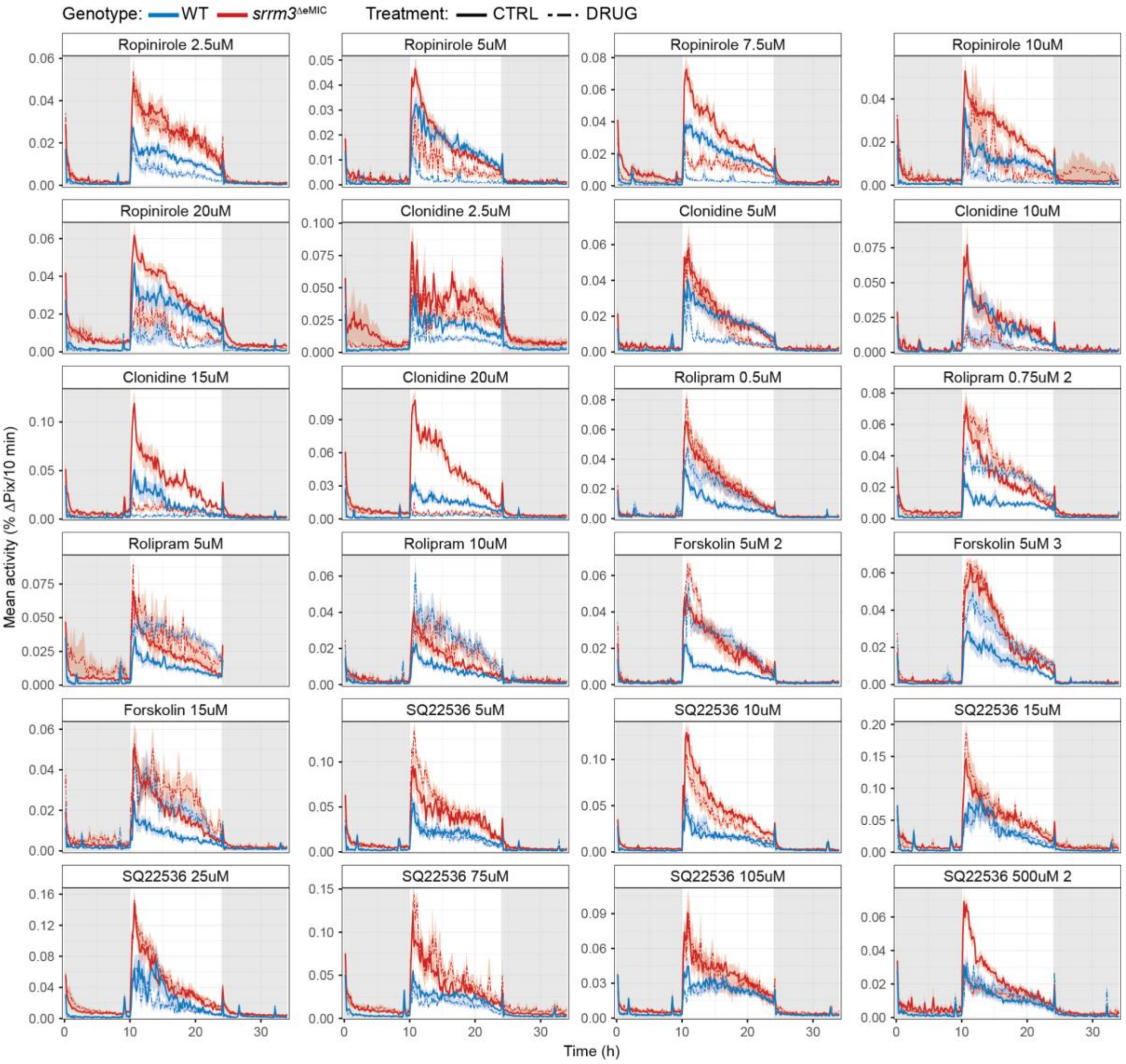

Supplementary Fig. 7. cAMP signalling is central to *srrm3*$^{\Delta eMIC}$ daytime hyperactivity. Mean locomotor activity (%ΔPix/10 min) of WT siblings (blue) and *srrm3*$^{\Delta eMIC}$ larvae (red) during a 34 h recording period (5-7 dpf) under a 14 h light / 10 h dark cycle (white/grey background respectively). Larvae were either drug-treated (dashed lines) with the indicated concentrations of ropinirole, clonidine, rolipram, forskolin, or SQ22536, or control-treated (solid lines). Traces show mean ± SEM across larvae from the same clutch, genotype, and treatment. n = 4-22 larvae per genotype and treatment.

Fig. S8.

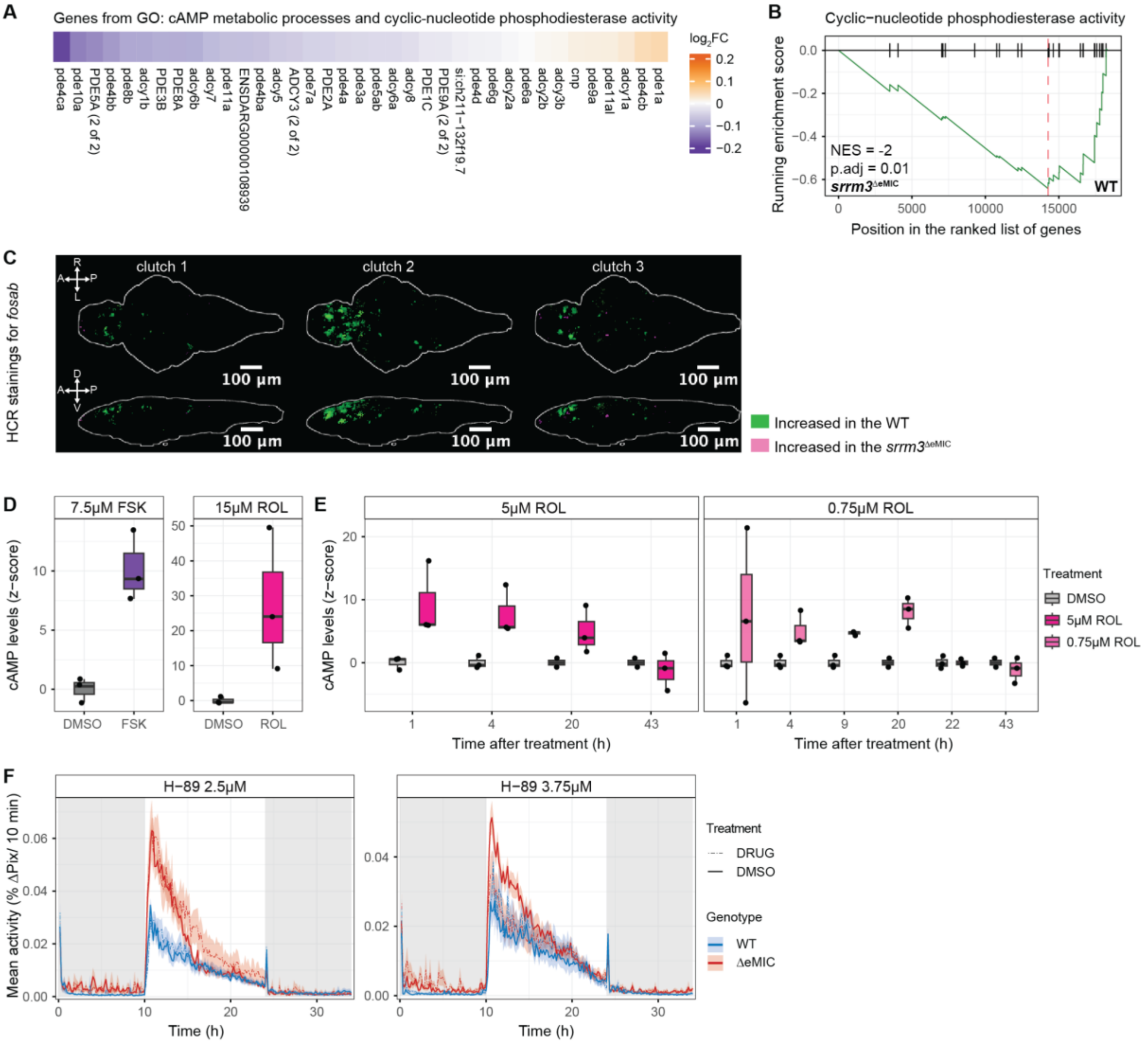

Supplementary Fig. 8. cAMP-PKA pathway misregulation and time-dependent cAMP dynamics in *srrm3*$^{\Delta eMIC}$ larvae.

(A) Log$_2$FC between WT and *srrm3*$^{\Delta eMIC}$ larvae for genes belonging to significantly enriched cAMP-related GO terms ("cyclic-nucleotide phosphodiesterase activity" and "cAMP metabolic process"). Purple indicates higher expression in the WT and orange in the *srrm3*$^{\Delta eMIC}$.

(B) GSEA of log$_2$FC values between WT and *srrm3*$^{\Delta eMIC}$ larvae for the GO term "cyclic-nucleotide phosphodiesterase activity."

(C) Maximum-intensity z-projections of MAPMapping-derived significant delta means for *fosab* HCR staining in WT and *srrm3*$^{\Delta eMIC}$ larvae (Zeitgeber [ZT]16), shown dorsally (top) and sagittally (bottom). Green indicates higher *fosab* in WT; pink indicates higher *fosab* in *srrm3*$^{\Delta eMIC}$. N = 3 clutches, 6-10 larvae per genotype. Orientation: A, anterior; P, posterior; R, rightward; L, leftward; D, dorsal; V, ventral.

(D) Total cAMP levels (z-score) in WT larvae (5 dpf) 1 h after treatment with forskolin (FSK) or rolipram (ROL) compared to DMSO-treated controls. Each dot represents a pool of 10 larvae. Comparisons were performed within clutch. n = 3 samples per condition.

(E) Time course of total cAMP levels (z-score) in WT larvae (5-7 dpf) following rolipram (ROL) treatment. Each dot represents a pool of 8-10 larvae. Comparisons at each concentration and time point were performed within clutch. n = 2-3 samples per treatment and time point.
(F) Mean locomotor activity (%ΔPix/10 min) of WT siblings (blue) and *srrm3*$^{\Delta eMIC}$ larvae (red), either H-89-treated (dashed) or DMSO-treated controls (solid), during a 34 h recording (5-7 dpf) under a 14 h light / 10 h dark cycle (white/grey background respectively). Traces show mean ± SEM. n = 6-15 larvae per genotype and treatment and clutch.

Fig. S9.

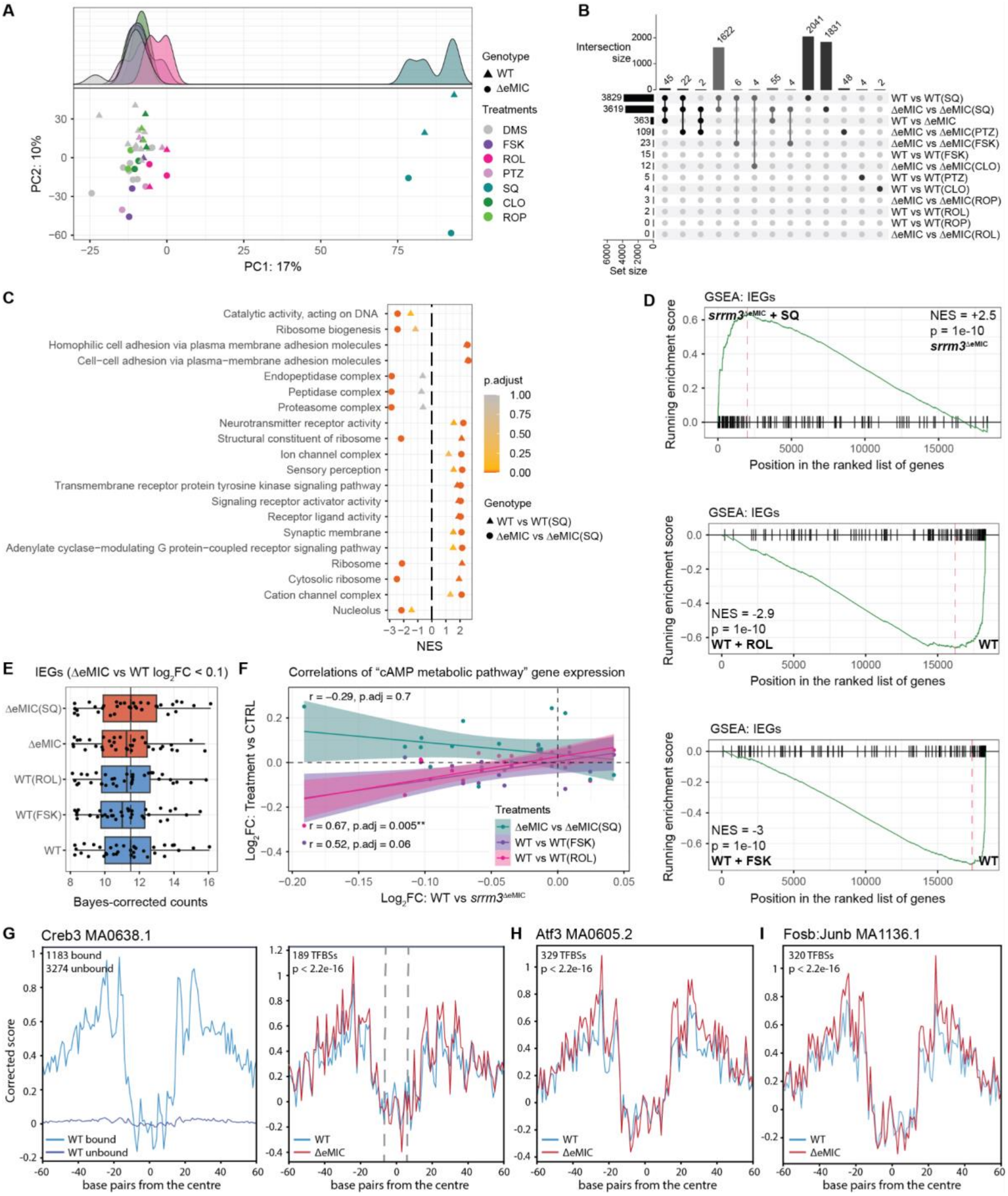

Supplementary Fig. 9. cAMP pathway modulators partially mimic and reverse *srrm3*$^{\Delta eMIC}$-induced gene expression changes.

(A) PCA of the 5,000 most variable genes after variance stabilisation and batch correction. PC1 separates SQ22536-treated samples from all other conditions. Each point represents one sample, shaped by genotype and coloured by treatment.

(B) UpSet plot showing overlaps (minimum overlap = 2) of DEGs ($p < 0.05$) across conditions; bar height indicates intersection size; grey shading indicates number of overlapping sets (1-3).

(C) Top 20 GO terms (p.adj < 0.05) enriched among DEGs in ΔeMIC vs ΔeMIC(SQ) plotted by normalized enrichment score (NES). Circles indicate ΔeMIC vs ΔeMIC(SQ); triangles indicate WT vs WT(SQ). Positive NES reflects higher expression in the treated condition.
(D) GSEAs of mouse ortholog immediate early genes (IEGs) (*61*), using all expressed genes ranked by log_2FC for ΔeMIC vs ΔeMIC(SQ) (top), WT vs WT(ROL) (middle), and WT vs WT(FSK) (bottom).
(E) Bayes-corrected counts of IEGs downregulated in ΔeMIC vs WT ($log_2FC < -0.1$). Each dot represents the median expression of one gene across clutches within a treatment group.
(F) Correlation of log_2FC values for cAMP metabolic pathway genes between WT vs *srrm3*$^{\Delta eMIC}$ and drug treatments: Mimic conditions (FSK, ROL) correlate positively [WT vs WT(FSK): r = 0.52, p.adj = 0.06; WT vs WT(ROL): r = 0.67, p.adj = 0.005], whereas rescue (SQ) shows an inverse trend [ΔeMIC vs ΔeMIC(SQ): r = -0.29, p.adj = 0.7]. Lines show linear regression fits. Pearson correlations with Bonferroni-adjusted p-values are shown.
(G–I) *TOBIAS* footprinting analyses (Table S1) showing differential transcription factor binding. **(G)** Creb3 binding sites (left: bound vs unbound in WT; right: sites with stronger binding in ΔeMIC [$log_2FC > 0.25$] compared to WT). **(H)** Atf3 and **(I)** Fosb:Junb binding sites enriched in ΔeMIC ($log_2FC > 0.25$) compared to WT. N = 1 biological replicate (11 larvae per sample). Transcription factor binding site (TFBS) counts are indicated; p-values are from *TOBIAS* across all sites. Genes associated with differential binding are listed in Data S3.
Treatment abbreviations: CLO, clonidine; DMSO, dimethyl sulfoxide; FSK, forskolin; PTZ, pentylenetetrazol; ROL, rolipram; ROP, ropinirole; SQ, SQ22536.

Table S1. Overview of key softwares/algorithms/packages

Context	Type	Description	Application	Ref.
**Behaviour**	Software/ algorithm	FramebyFrame	Behavioural parameters	Kroll *et al.,* 2025 (*101*)
**2P imaging**	Software/ algorithm	Advanced normalization tools (ANTs) (v2.3.5)	Image registration	Avants *et al.*, 2021 (*121*)
**2P imaging**	Software/ algorithm	MATLAB script for cell detection	ROI extraction	Kawashima *et al.,* 2016 (*122*)
**2P imaging**	Software/ algorithm	Zebrafish Brain Browser (ZBB)	Reference for image registration	Marquart *et al.,* 2015 (*123*), Marquart *et al.,* 2017 (*124*)
**2P imaging**	Software/ algorithm	MATLAB script for motion correction	Motion correction	Bianco and Engert 2015 (*105*)
**2P imaging**	Software/ algorithm	Online Active Set method to Infer Spikes (OASIS)	Deconvolution of calcium data	Friedrich *et al.,* 2017 (*51*)
**scRNA-seq**	R package	findPC (v1.0)	PC selection	Zhuang *et al.,* 2022 (*113*)
**scRNA-seq**	R package	speckle (v1.4.0), propeller	Testing for differences in cell type proportions	Phipson *et al.,* 2022 (*125*)
**scRNA-seq**	R package	Adaptively-thresholded Low Rank Approximation (*ALRA)* (v0.0.0.9000)	Zero-preserving imputation	Linderman *et al.,* 2022 (*126*)
**scRNA-seq**	R package	clusterProfiler (v4.12.0)	GSEA	Wu *et al.,* 2021 (*127*)
**scRNA-seq**	Software/ algorithm	DanioCell	Assigning cluster identities	Shainer *et al.,* 2023 (*128*)
**scRNA-seq**	R package	Seurat (v4.3.0)	Analysis pipeline	Stuart *et al.,* 2019 (*129*)
**RNA-seq**	Software/ algorithm	Salmon (v1.5.1)	Alignment, transcript expression quantification	Patro *et al.,* 2017 (*130*)
**RNA-seq**	R package	tximport (v1.32.0)	Importing transcript-level abundance	Soneson *et al.,* 2015 (*131*)

			estimates from Salmon into R for count-based statistical inferences	
**RNA-seq**	R package	limma (v3.60.0)	Differential expression analysis	Ritchie *et al.,* 2015 (*132*)
**RNA-seq**	R package	DESeq2 (v1.44.0)	Differential expression analysis	Love *et al.,* 2014 (*133*)
**RNA-seq**	R package	Weighted correlation network analysis (*WGCNA*) (v1.72.5)	Removing of batch effects	Langfelder and Horvath 2008 (*134*)
**RNA-seq**	Software/ algorithm	STRINGdb (v2.16.0)	Protein-protein interaction	Franceschini *et al.,* 2013 (*135*)
**RNA-seq**	Software/ algorithm	Ensembl biomart (v112)	Resources for genomes and annotations	Harrison *et al.,* 2024 (*136*)
**AS**	Software/ algorithm	vast-tools (v2.5.1) and Vast database (VastDB)	AS analysis, annotations	Tapial *et al.,* 2017 (*17*)
**AS**	R package	betAS (v1.2.0)	Statistical comparisons of AS events	Ascensão-Ferreira *et al.,* 2024 (*137*)
**HCR**	Software/ algorithm	MapMAPPING	Brain volume and pixel-wise intensity comparisons	Randlett *et al.,* 2015 (*110*), Thyme *et al.,* 2019 (*138*)
**HCR**	Software/ algorithm	Computational Morphometry Toolkit (*CMTK*)	Brain image registration	Rohlfing 2011 (*139*)
**HCR**	Python package	scikit-image (v0.26.0)	Multi-Otsu thresholding background vs signal	Liao *et al.,* 2001 (*140*)
**ATAC-seq**	Software/ algorithm	trimmomatic (v0.39)	Read trimming	Bolger *et al.,* 2014 (*141*)
**ATAC-seq**	Software/ algorithm	bwa (v0.7.18-r1243-dirty)	Read alignment	Li 2013 (*142*)
**ATAC-seq**	Software/ algorithm	samtools (v1.21)	Read sorting	Li *et al.,* 2009 (*143*)
**ATAC-seq**	Software/ algorithm	deeptools (v3.5.5)	Tn5 offset-correction and read filtering	Ramírez *et al.,* 2016 (*144*)

**ATAC-seq**	Software/ algorithm	macs3 (v3.0.2)	Peak calling	Zhang *et al.,* 2008 (*145*)
**ATAC-seq**	Software/ algorithm	featureCounts (v2.0.8)	Read counts per peak	Liao *et al.,* 2014 (*146*)
**ATAC-seq**	Software/ algorithm	TOBIAS (v0.17.1)	Differential motif occupancy; footprinting	Bentsen *et al.,* 2020 (*147*)

Overview of key softwares/algorithms and packages used for this study, detailing the context the tool was used in and appropriate references.

Data S1. (separate file)
Statistics and sample sizes of pixel-based behavioural assays. Model estimates ± SE and sample sizes for the thigmotaxis assay, the dark-light assay, the tapping habituation assay and all sleep/wake parameters tested (*102*) for replicates of *srrm3* the *vsx1;vsx2* experiment and the *srrm3;srrm4* (Fig. 1) but also for the individual microexon mutants (Fig. 4J).

Data S2. (separate file)
Statistics and sample sizes for the bout type and bout kinematics analysis. Sample sizes and statistics for bout usage and kinematics, long bouts and PCA/Euclidean distance, specifying when individual bouts or larvae were excluded from the analysis (Fig. 2).

Data S3. (separate file)
ATAC-seq: Footprinting output using *TOBIAS*. Differential binding scores from *TOBIAS* (Fig. 8G). TFBSs associated with significant Creb-related motifs with information on the peak annotation and gene (ENSEMBL ID).

Data S4. (separate file)
Metadata for sequencing experiments. Information about bulk (Fig. 4, Fig. 6, Fig. 7), single-cell (Fig. 4, Fig. 5), and ATAC-sequencing experiments (Fig. 8), including sample size, treatments, RNA integrity number (RIN), life cells (%) after FACS-sorting, paired vs single end, sequencing depth, read length, mappability, the type of analysis the data was used for.

Data S5. (separate file)
HCR probes. Sequences of the oligonucleotides ordered for the HCR probes of *fosab* and *srrm3*.

Data S6. (separate file)
Gene- and exon lists. DEGs between WT and *srrm3*$^{\Delta eMIC}$ (Fig. S4A, bulk 1); marker genes from the single-cell RNA-seq data (Fig. 4B, Fig. S4B, C); DEGs per cluster from the single-cell RNA-seq data (Fig. 5E); PSI comparing exon inclusion between WT and *srrm3*$^{\Delta eMIC}$ (Fig. 4D-I, bulk 1); DEGs between WT and *srrm3*$^{\Delta eMIC}$ (Fig. 5, bulk 2); List of GO terms for up- and downregulated genes between WT and *srrm3*$^{\Delta eMIC}$ (Fig. 5C, bulk 2); DEGs between WT, *srrm3*$^{+/\Delta eMIC}$ and *srrm3*$^{\Delta eMIC}$ (Fig. S5, bulk 3); PSI comparing exon inclusion between WT, *srrm3*$^{+/\Delta eMIC}$ and *srrm3*$^{\Delta eMIC}$ (Fig. S5, bulk 3); DEGs between drug-treated WT and *srrm3*$^{\Delta eMIC}$ compared to their within-genotype matched controls (Fig. 8, bulk 2).

REFERENCES

1. J. K. Kanwal, E. Coddington, R. Frazer, D. Limbania, G. Turner, K. J. Davila, M. A. Givens, V. Williams, S. R. Datta, S. Wasserman, Internal state: Dynamic, interconnected communication loops distributed across body, brain, and time. *Integr. Comp. Biol.* **61**, 867–886 (2021).

2. L. C. Mayes, A developmental perspective on the regulation of arousal states. *Semin. Perinatol.* **24**, 267–279 (2000).

3. T. W. Robbins, Arousal systems and attentional processes. *Biol. Psychol.* **45**, 57–71 (1997).

4. C. H. Poth, Readiness for perception and action: Towards a more mechanistic understanding of phasic alertness. *J. Cogn.* **8**, 19 (2025).

5. S. J. Lane, M. A. Leão, V. Spielmann, Sleep, sensory integration/processing, and autism: A scoping review. *Front. Psychol.* **13**, 877527 (2022).

6. R. D. Nath, C. N. Bedbrook, M. J. Abrams, T. Basinger, J. S. Bois, D. A. Prober, P. W. Sternberg, V. Gradinaru, L. Goentoro, The jellyfish Cassiopea exhibits a sleep-like state. *Curr. Biol.* **27**, 2984–2990.e3 (2017).

7. J. C. Hendricks, S. M. Finn, K. A. Panckeri, J. Chavkin, J. A. Williams, A. Sehgal, A. I. Pack, Rest in Drosophila is a sleep-like state. *Neuron* **25**, 129–138 (2000).

8. P. J. Shaw, C. Cirelli, R. J. Greenspan, G. Tononi, Correlates of sleep and waking in Drosophila melanogaster. *Science* **287**, 1834–1837 (2000).

9. I. V. Zhdanova, S. Y. Wang, O. U. Leclair, N. P. Danilova, Melatonin promotes sleep-like state in zebrafish. *Brain Res.* **903**, 263–268 (2001).

10. D. A. Prober, J. Rihel, A. A. Onah, R.-J. Sung, A. F. Schier, Hypocretin/orexin overexpression induces an insomnia-like phenotype in zebrafish. *J. Neurosci.* **26**, 13400–13410 (2006).

11. T. Yokogawa, W. Marin, J. Faraco, G. Pézeron, L. Appelbaum, J. Zhang, F. Rosa, P. Mourrain, E. Mignot, Characterization of sleep in zebrafish and insomnia in hypocretin receptor mutants. *PLOS Biol.* **5**, e277 (2007).

12. M. Hirshkowitz, Normal human sleep: An overview. *Med. Clin. North Am.* **88**, 551–565 (2004).

13. P. Peirano, C. Algarín, R. Uauy, Sleep-wake states and their regulatory mechanisms throughout early human development. *J. Pediatr.* **143**, S70–S79 (2003).

14. C. N. Chiu, D. A. Prober, Regulation of zebrafish sleep and arousal states: Current and prospective approaches. *Front. Neural Circuits* **7**, 58 (2013).

15. P. V. Nguyen, N. H. Woo, Regulation of hippocampal synaptic plasticity by cyclic AMP-dependent protein kinases. *Prog. Neurobiol.* **71**, 401–437 (2003).

16. C. C. Y. Leung, Y. H. Wong, Role of G protein-coupled receptors in the regulation of structural plasticity and cognitive function. *Molecules* **22**, 1239 (2017).

17. J. Tapial, K. C. H. Ha, T. Sterne-Weiler, A. Gohr, U. Braunschweig, A. Hermoso-Pulido, M. Quesnel-Vallières, J. Permanyer, R. Sodaei, Y. Marquez, L. Cozzuto, X. Wang, M. Gómez-Velázquez, T. Rayon, M. Manzanares, J. Ponomarenko, B. J. Blencowe, M. Irimia, An atlas of alternative splicing profiles and functional associations reveals new regulatory programs and genes that simultaneously express multiple major isoforms. *Genome Res.* **27**, 1759–1768 (2017).

18. D. Schmucker, J. C. Clemens, H. Shu, C. A. Worby, J. Xiao, M. Muda, J. E. Dixon, S. L. Zipursky, Drosophila Dscam is an axon guidance receptor exhibiting extraordinary molecular diversity. *Cell* **101**, 671–684 (2000).

19. M. Irimia, R. J. Weatheritt, J. D. Ellis, N. N. Parikshak, T. Gonatopoulos-Pournatzis, M. Babor, M. Quesnel-Vallières, J. Tapial, B. Raj, D. O'Hanlon, M. Barrios-Rodiles, M. J. E. Sternberg, S. P. Cordes, F. P. Roth, J. L. Wrana, D. H. Geschwind, B. J. Blencowe, A highly

conserved program of neuronal microexons is misregulated in autistic brains. *Cell* **159**, 1511–1523 (2014).

20. Y. I. Li, L. Sanchez-Pulido, W. Haerty, C. P. Ponting, RBFOX and PTBP1 proteins regulate the alternative splicing of micro-exons in human brain transcripts. *Genome Res.* **25**, 1–13 (2015).

21. A. Torres-Méndez, S. Pop, S. Bonnal, I. Almudi, A. Avola, R. J. V. Roberts, C. Paolantoni, A. Alcaina-Caro, A. Martín-Anduaga, I. U. Haussmann, V. Morin, F. Casares, M. Soller, S. Kadener, J.-Y. Roignant, L. Prieto-Godino, M. Irimia, Parallel evolution of a splicing program controlling neuronal excitability in flies and mammals. *Sci. Adv.* **8**, eabk0445 (2022).

22. T. Gonatopoulos-Pournatzis, M. Wu, U. Braunschweig, J. Roth, H. Han, A. J. Best, B. Raj, M. Aregger, D. O'Hanlon, J. D. Ellis, J. A. Calarco, J. Moffat, A.-C. Gingras, B. J. Blencowe, Genome-wide CRISPR-Cas9 interrogation of splicing networks reveals a mechanism for recognition of autism-misregulated neuronal microexons. *Mol. Cell* **72**, 510–524.e12 (2018).

23. Y. Nakano, S. Wiechert, B. Bánfi, Overlapping activities of two neuronal splicing factors switch the GABA effect from excitatory to inhibitory by regulating REST. *Cell Rep.* **27**, 860–871.e8 (2019).

24. A. Torres-Méndez, S. Bonnal, Y. Marquez, J. Roth, M. Iglesias, J. Permanyer, I. Almudí, D. O'Hanlon, T. Guitart, M. Soller, A.-C. Gingras, F. Gebauer, F. Rentzsch, B. J. Blencowe, J. Valcárcel, M. Irimia, A novel protein domain in an ancestral splicing factor drove the evolution of neural microexons. *Nat. Ecol. Evol.* **3**, 691–701 (2019).

25. M. Quesnel-Vallières, M. Irimia, S. P. Cordes, B. J. Blencowe, Essential roles for the splicing regulator nSR100/SRRM4 during nervous system development. *Genes Dev.* **29**, 746–759 (2015).

26. K. A. Han, T.-H. Yoon, J. Kim, J. Lee, J. Y. Lee, G. Jang, J. W. Um, J. K. Kim, J. Ko, Specification of neural circuit architecture shaped by context-dependent patterned LAR-RPTP microexons. *Nat. Commun.* **15**, 1624 (2024).

27. T. Mackensen, M. Irimia, From tiny exons to big insights: The expanding field of microexons. *Annu. Rev. Genomics Hum. Genet.* **26**, 77–102 (2025).

28. H. Park, Y. Choi, H. Jung, S. Kim, S. Lee, H. Han, H. Kweon, S. Kang, W. S. Sim, F. Koopmans, E. Yang, H. Kim, A. B. Smit, Y. C. Bae, E. Kim, Splice-dependent trans-synaptic PTPδ-IL1RAPL1 interaction regulates synapse formation and non-REM sleep. *EMBO J.* **39**, e104150 (2020).

29. L. Lopez-Blanch, C. Rodríguez-Marin, F. Mantica, L. P. Iñiguez, J. Permanyer, E. M. Kita, T. Mackensen, M. Codina-Tobias, F. Romero-Ferrero, J. Fernandez-Albert, M. Cuadrado, X. R. Bustelo, G. de Polavieja, M. Irimia, Phenotypic impact of individual conserved neuronal microexons and their master regulators in zebrafish. *Elife* **13**, RP104275 (2025).

30. M. Quesnel-Vallières, Z. Dargaei, M. Irimia, T. Gonatopoulos-Pournatzis, J. Y. Ip, M. Wu, T. Sterne-Weiler, S. Nakagawa, M. A. Woodin, B. J. Blencowe, S. P. Cordes, Misregulation of an activity-dependent splicing network as a common mechanism underlying autism spectrum disorders. *Mol. Cell* **64**, 1023–1034 (2016).

31. C. Garcia-Cabau, A. Bartomeu, G. Tesei, K. C. Cheung, J. Pose-Utrilla, S. Picó, A. Balaceanu, B. Duran-Arqué, M. Fernández-Alfara, J. Martín, C. De Pace, L. Ruiz-Pérez, J. García, G. Battaglia, J. J. Lucas, R. Hervás, K. Lindorff-Larsen, R. Méndez, X. Salvatella, Mis-splicing of a neuronal microexon promotes CPEB4 aggregation in ASD. *Nature* **637**, 496–503 (2025).

32. A. Parras, H. Anta, M. Santos-Galindo, V. Swarup, A. Elorza, J. L. Nieto-González, S. Picó, I. H. Hernández, J. I. Díaz-Hernández, E. Belloc, A. Rodolosse, N. N. Parikshak, O. Peñagarikano, R. Fernández-Chacón, M. Irimia, P. Navarro, D. H. Geschwind, R. Méndez, J. J. Lucas, Autism-like phenotype and risk gene mRNA deadenylation by CPEB4 mis-splicing. *Nature* **560**, 441–446 (2018).

33. I. Ollà, A. F. Pardiñas, A. Parras, I. H. Hernández, M. Santos-Galindo, S. Picó, L. F. Callado, A. Elorza, C. Rodríguez-López, G. Fernández-Miranda, E. Belloc, J. T. R. Walters, M. C. O'Donovan, R. Méndez, C. Toma, J. J. Meana, M. J. Owen, J. J. Lucas, Pathogenic mis-splicing of CPEB4 in schizophrenia. *Biol. Psychiatry* **94**, 341–351 (2023).

34. F. Ferrarelli, Sleep abnormalities in schizophrenia: State of the art and next steps. *Am. J. Psychiatry* **178**, 903–913 (2021).

35. J. C. Marques, S. Lackner, R. Félix, M. B. Orger, Structure of the zebrafish locomotor repertoire revealed with unsupervised behavioral clustering. *Curr. Biol.* **28**, 181–195.e5 (2018).

36. T. Mueller, M. F. Wullimann, Anatomy of neurogenesis in the early zebrafish brain. *Brain Res. Dev. Brain Res.* **140**, 137–155 (2003).

37. L. Ciampi, F. Mantica, L. López-Blanch, J. Permanyer, C. Rodriguez-Marín, J. Zang, D. Cianferoni, S. Jiménez-Delgado, S. Bonnal, S. Miravet-Verde, V. Ruprecht, S. C. F. Neuhauss, S. Banfi, S. Carrella, L. Serrano, S. A. Head, M. Irimia, Specialization of the photoreceptor transcriptome by *Srrm3*-dependent microexons is required for outer segment maintenance and vision. *Proc. Natl. Acad. Sci. U.S.A.* **119**, e2117090119 (2022).

38. J. Letelier, L. Buono, M. Almuedo-Castillo, J. Zang, C. Mounieres, S. González-Díaz, R. Polvillo, E. Sanabria-Reinoso, J. Corbacho, A. Sousa-Ortega, R. Diez Del Corral, S. C. F. Neuhauss, J. R. Martínez-Morales, Mutation of *vsx* genes in zebrafish highlights the robustness of the retinal specification network. *Elife* **12**, e85594 (2023).

39. F. Emran, J. Rihel, J. E. Dowling, A behavioral assay to measure responsiveness of zebrafish to changes in light intensities. *J. Vis. Exp.* **20**, 923 (2008).

40. Y.-J. Wang, S.-J. Cai, J.-L. Cui, Y. Chen, X. Tang, Y.-H. Li, Correlation between photoreceptor injury-regeneration and behavior in a zebrafish model. *Neural Regen. Res.* **12**, 795–803 (2017).

41. S. Banerjee, L. E. Ranspach, X. Luo, L. T. Cianciolo, J. Fogerty, B. D. Perkins, R. Thummel, Vision and sensorimotor defects associated with loss of Vps11 function in a zebrafish model of genetic leukoencephalopathy. *Sci. Rep.* **12**, 3511 (2022).

42. S. C. Baraban, M. R. Taylor, P. A. Castro, H. Baier, Pentylenetetrazole induced changes in zebrafish behavior, neural activity and c-fos expression. *Neuroscience* **131**, 759–768 (2005).

43. S. J. Schnörr, P. J. Steenbergen, M. K. Richardson, D. L. Champagne, Measuring thigmotaxis in larval zebrafish. *Behav. Brain Res.* **228**, 367–374 (2012).

44. S. Pintos, T. Lucon-Xiccato, L. M. Vera, C. Bertolucci, Daily rhythms in the behavioural stress response of the zebrafish Danio rerio. *Physiol. Behav.* **268**, 114241 (2023).

45. A. H. Groneberg, J. C. Marques, A. L. Martins, R. Diez Del Corral, G. G. de Polavieja, M. B. Orger, Early-life social experience shapes social avoidance reactions in larval zebrafish. *Curr. Biol.* **30**, 4009–4021.e4 (2020).

46. A. S. Andalman, V. M. Burns, M. Lovett-Barron, M. Broxton, B. Poole, S. J. Yang, L. Grosenick, T. N. Lerner, R. Chen, T. Benster, P. Mourrain, M. Levoy, K. Rajan, K. Deisseroth, Neuronal dynamics regulating brain and behavioral state transitions. *Cell* **177**, 970–985.e20 (2019).

47. J. C. Marques, M. Li, D. Schaak, D. N. Robson, J. M. Li, Internal state dynamics shape brainwide activity and foraging behaviour. *Nature* **577**, 239–243 (2020).

48. L. Turrini, M. Sorelli, G. de Vito, C. Credi, N. Tiso, F. Vanzi, F. S. Pavone, Multimodal characterization of seizures in zebrafish larvae. *Biomedicines* **10**, 951 (2022).

49. C. Singh, G. Oikonomou, D. A. Prober, Norepinephrine is required to promote wakefulness and for hypocretin-induced arousal in zebrafish. *Elife* **4**, e07000 (2015).

50. N. Vladimirov, Y. Mu, T. Kawashima, D. V. Bennett, C.-T. Yang, L. L. Looger, P. J. Keller, J. Freeman, M. B. Ahrens, Light-sheet functional imaging in fictively behaving zebrafish. *Nat. Methods* **11**, 883–884 (2014).

51. J. Friedrich, P. Zhou, L. Paninski, Fast online deconvolution of calcium imaging data. *PLOS Comput. Biol.* **13**, e1005423 (2017).

52. T.-W. Chen, T. J. Wardill, Y. Sun, S. R. Pulver, S. L. Renninger, A. Baohan, E. R. Schreiter, R. A. Kerr, M. B. Orger, V. Jayaraman, L. L. Looger, K. Svoboda, D. S. Kim, Ultrasensitive fluorescent proteins for imaging neuronal activity. *Nature* **499**, 295–300 (2013).

53. K. Wang, J. Hinz, Y. Zhang, T. R. Thiele, A. B. Arrenberg, Parallel channels for motion feature extraction in the pretectum and tectum of larval zebrafish. *Cell Rep.* **30**, 442–453.e6 (2020).

54. A. M. Fernandes, D. S. Mearns, J. C. Donovan, J. Larsch, T. O. Helmbrecht, Y. Kölsch, E. Laurell, K. Kawakami, M. Dal Maschio, H. Baier, Neural circuitry for stimulus selection in the zebrafish visual system. *Neuron* **109**, 805–822.e6 (2021).

55. C. C. S. Calhoun, M. E. S. Capps, K. Muya, W. C. Gannaway, V. Martina, C. L. Conklin, M. C. Klein, J. M. Webster, E. G. Torija-Olson, S. B. Thyme, Removal of developmentally regulated microexons has a minimal impact on larval zebrafish brain morphology and function. *eLife* **13**, RP101790 (2025).

56. D. Vecellio Reane, C. Cerqua, S. Sacconi, L. Salviati, E. Trevisson, A. Raffaello, The splicing of the mitochondrial calcium Uniporter genuine activator MICU1 is driven by RBFOX2 splicing factor during myogenic differentiation. *Int. J. Mol. Sci.* **23**, 2517 (2022).

57. R. A. Jain, M. A. Wolman, K. C. Marsden, J. C. Nelson, H. Shoenhard, F. A. Echeverry, C. Szi, H. Bell, J. Skinner, E. N. Cobbs, K. Sawada, A. D. Zamora, A. E. Pereda, M. Granato, A forward genetic screen in zebrafish identifies the G-protein-coupled receptor CaSR as a modulator of sensorimotor decision making. *Curr. Biol.* **28**, 1357–1369.e5 (2018).

58. J. P. Barrios, W.-C. Wang, R. England, E. Reifenberg, A. D. Douglass, Hypothalamic dopamine neurons control sensorimotor behavior by modulating brainstem premotor nuclei in zebrafish. *Curr. Biol.* **30**, 4606–4618.e4 (2020).

59. R. Maggio, M. Scarselli, F. Novi, M. J. Millan, G. U. Corsini, Potent activation of dopamine D_3/D_2 heterodimers by the antiparkinsonian agents, S32504, pramipexole and ropinirole. *J. Neurochem.* **87**, 631–641 (2003).

60. D. Kurko, Z. Kapui, J. Nagy, B. Lendvai, S. Kolok, Analysis of functional selectivity through G protein-dependent and -independent signaling pathways at the adrenergic α_{2C} receptor. *Brain Res. Bull.* **107**, 89–101 (2014).

61. Y. E. Wu, L. Pan, Y. Zuo, X. Li, W. Hong, Detecting activated cell populations using single-cell RNA-seq. *Neuron* **96**, 313–329.e6 (2017).

62. M. Sheng, G. McFadden, M. E. Greenberg, Membrane depolarization and calcium induce c-fos transcription via phosphorylation of transcription factor CREB. *Neuron* **4**, 571–582 (1990).

63. X. Tao, S. Finkbeiner, D. B. Arnold, A. J. Shaywitz, M. E. Greenberg, Ca^{2+} influx regulates BDNF transcription by a CREB family transcription factor-dependent mechanism. *Neuron* **20**, 709–726 (1998).

64. E. Benito, L. M. Valor, M. Jimenez-Minchan, W. Huber, A. Barco, cAMP response element-binding protein is a primary hub of activity-driven neuronal gene expression. *J. Neurosci.* **31**, 18237–18250 (2011).

65. Y. Furukawa-Hibi, J. Yun, T. Nagai, K. Yamada, Transcriptional suppression of the neuronal PAS domain 4 (Npas4) gene by stress via the binding of agonist-bound glucocorticoid receptor to its promoter. *J. Neurochem.* **123**, 866–875 (2012).

66. J. B. Calais, S. S. Valvassori, W. R. Resende, G. Feier, M. C. P. Athié, S. Ribeiro, W. F. Gattaz, J. Quevedo, E. B. Ojopi, Long-term decrease in immediate early gene expression after electroconvulsive seizures. *J. Neural Transm. (Vienna)* **120**, 259–266 (2013).

67. R. Sasson, E. Rimon, A. Dantes, T. Cohen, V. Shinder, A. Land-Bracha, A. Amsterdam, Gonadotrophin-induced gene regulation in human granulosa cells obtained from IVF patients. Modulation of steroidogenic genes, cytoskeletal genes and genes coding for apoptotic signalling and protein kinases. *Mol. Hum. Reprod.* **10**, 299–311 (2004).

68. G. W. Reierson, C. A. Mastronardi, J. Licinio, M.-L. Wong, Chronic imipramine downregulates cyclic AMP signaling in rat hippocampus. *Neuroreport* **20**, 307–311 (2009).

69. P. R. Lundegaard, C. Anastasaki, N. J. Grant, R. R. Sillito, J. Zich, Z. Zeng, K. Paranthaman, A. P. Larsen, J. D. Armstrong, D. J. Porteous, E. E. Patton, MEK inhibitors reverse cAMP-mediated anxiety in zebrafish. *Chem. Biol.* **22**, 1335–1346 (2015).

70. J. Vandamme, D. Castermans, J. M. Thevelein, Molecular mechanisms of feedback inhibition of protein kinase A on intracellular cAMP accumulation. *Cell. Signal.* **24**, 1610–1618 (2012).

71. M. M. Reimer, A. Norris, J. Ohnmacht, R. Patani, Z. Zhong, T. B. Dias, V. Kuscha, A. L. Scott, Y.-C. Chen, S. Rozov, S. L. Frazer, C. Wyatt, S.-I. Higashijima, E. E. Patton, P. Panula, S. Chandran, T. Becker, C. G. Becker, Dopamine from the brain promotes spinal motor neuron generation during development and adult regeneration. *Dev. Cell* **25**, 478–491 (2013).

72. J. Fernandez-Albert, M. Lipinski, M. T. Lopez-Cascales, M. J. Rowley, A. M. Martin-Gonzalez, B. Del Blanco, V. G. Corces, A. Barco, Immediate and deferred epigenomic signatures of in vivo neuronal activation in mouse hippocampus. *Nat. Neurosci.* **22**, 1718–1730 (2019).

73. A. Steven, M. Friedrich, P. Jank, N. Heimer, J. Budczies, C. Denkert, B. Seliger, What turns CREB on? And off? And why does it matter? *Cell. Mol. Life Sci.* **77**, 4049–4067 (2020).

74. Z. Wu, M. Nicoll, R. J. Ingham, AP-1 family transcription factors: A diverse family of proteins that regulate varied cellular activities in classical hodgkin lymphoma and ALK+ ALCL. *Exp. Hematol. Oncol.* **10**, 4 (2021).

75. T. M. Reed, D. R. Repaske, G. L. Snyder, P. Greengard, C. V. Vorhees, Phosphodiesterase 1B knock-out mice exhibit exaggerated locomotor hyperactivity and DARPP-32 phosphorylation in response to dopamine agonists and display impaired spatial learning. *J. Neurosci.* **22**, 5188–5197 (2002).

76. L. Yang, L.-J. Shi, J. Yu, Y.-Q. Zhang, Activation of protein kinase A in the amygdala modulates anxiety-like behaviors in social defeat exposed mice. *Mol. Brain* **9**, 3 (2016).

77. D. Chen, J. Wang, J. Cao, G. Zhu, cAMP-PKA signaling pathway and anxiety: Where do we go next? *Cell. Signal.* **122**, 111311 (2024).

78. M. Sheng, M. E. Greenberg, The regulation and function of c-fos and other immediate early genes in the nervous system. *Neuron* **4**, 477–485 (1990).

79. L. F. Michael, H. Asahara, A. I. Shulman, W. L. Kraus, M. Montminy, The phosphorylation status of a cyclic AMP-responsive activator is modulated via a chromatin-dependent mechanism. *Mol. Cell. Biol.* **20**, 1596–1603 (2000).

80. J. R. Casanova, M. Nishimura, J. W. Swann, The effects of early-life seizures on hippocampal dendrite development and later-life learning and memory. *Brain Res. Bull.* **103**, 39–48 (2014).

81. C. E. Vásquez, R. Riener, E. Reynolds, G. B. Britton, NMDA receptor dysregulation in chronic state: A possible mechanism underlying depression with BDNF downregulation. *Neurochem. Int.* **79**, 88–97 (2014).

82. C. M. Atkins, M. C. Falo, O. F. Alonso, H. M. Bramlett, W. D. Dietrich, Deficits in ERK and CREB activation in the hippocampus after traumatic brain injury. *Neurosci. Lett.* **459**, 52–56 (2009).

83. H. Bito, K. Deisseroth, R. W. Tsien, CREB phosphorylation and dephosphorylation: A Ca^{2+}- and stimulus duration-dependent switch for hippocampal gene expression. *Cell* **87**, 1203–1214 (1996).

84. H. Wang, J. Xu, P. Lazarovici, R. Quirion, W. Zheng, CAMP response element-binding protein (CREB): A possible signaling molecule link in the pathophysiology of schizophrenia. *Front. Mol. Neurosci.* **11**, 255 (2018).

85. N. Sugo, Y. Atsumi, N. Yamamoto, Transcription and epigenetic factor dynamics in neuronal activity-dependent gene regulation. *Trends Genet.* **41**, 425–436 (2025).

86. F. Mantica, M. Irimia, Gene duplication and alternative splicing as evolutionary drivers of proteome specialization. *Bioessays* **47**, e202400202 (2025).

87. A. C. Emery, M. V. Eiden, T. Mustafa, L. E. Eiden, Rapgef2 connects GPCR-mediated cAMP signals to ERK activation in neuronal and endocrine cells. *Sci. Signal.* **6**, ra51 (2013).

88. S. T. Wong, J. Athos, X. A. Figueroa, V. V. Pineda, M. L. Schaefer, C. C. Chavkin, L. J. Muglia, D. R. Storm, Calcium-stimulated adenylyl cyclase activity is critical for hippocampus-dependent long-term memory and late phase LTP. *Neuron* **23**, 787–798 (1999).

89. N. T. Hofer, A. Pinggera, Y. V. Nikonishyna, P. Tuluc, E. M. Fritz, G. J. Obermair, J. Striessnig, Stabilization of negative activation voltages of Cav1.3 L-Type Ca^{2+}-channels by alternative splicing. *Channels (Austin)* **15**, 38–52 (2021).

90. M. Srinivasan, C. F. Edman, H. Schulman, Alternative splicing introduces a nuclear localization signal that targets multifunctional CaM kinase to the nucleus. *J. Cell Biol.* **126**, 839–852 (1994).

91. J. Juan-Mateu, S. Bajew, M. Miret-Cuesta, L. P. Íñiguez, A. Lopez-Pascual, S. Bonnal, G. Atla, S. Bonàs-Guarch, J. Ferrer, J. Valcárcel, M. Irimia, Pancreatic microexons regulate islet function and glucose homeostasis. *Nat. Metab.* **5**, 219–236 (2023).

92. J. F. García-Moreno, L. Romão, Perspective in alternative splicing coupled to nonsense-mediated mRNA decay. *Int. J. Mol. Sci.* **21**, 9424 (2020).

93. Y. Li, P. Zhang, T.-Y. Choi, S. K. Park, H. Park, E.-J. Lee, D. Lee, J. D. Roh, W. Mah, R. Kim, Y. Kim, H. Kwon, Y. C. Bae, S.-Y. Choi, A. M. Craig, E. Kim, Splicing-dependent trans-synaptic SALM3-LAR-RPTP interactions regulate excitatory synapse development and locomotion. *Cell Rep.* **12**, 1618–1630 (2015).

94. M. Toutant, J. M. Studler, F. Burgaya, A. Costa, P. Ezan, M. Gelman, J. A. Girault, Autophosphorylation of Tyr397 and its phosphorylation by Src-family kinases are altered in focal-adhesion-kinase neuronal isoforms. *Biochem. J.* **348**, 119–128 (2000).

95. C. Griffin, K. Coppenrath, D. Khan, Z. Lin, M. Horb, J.-P. Saint-Jeannet, Deletion of *sf3b4* causes splicing defects and gene dysregulation that disrupt craniofacial development and survival. *Dis. Model. Mech.* **18**, dmm052169 (2025).

96. L. Zixuan, P. Wenling, D. Xueling, H. Qing, Z. Zeping, S. Yaxuan, Role of the cAMP-PKA-CREB pathway in depression: Mechanisms and therapeutic implications. *Brain Res.* **1865**, 149895 (2025).

97. A. Martinez-Pizarro, S. Picó, M. Alvárez, J. Pose-Utrilla, L. L. Holm, T. K. Doktor, B. S. Andresen, J. J. Lucas, L. R. Desviat, Oligonucleotides targeting the 3' splice site downstream of a microexon as an innovative therapy for autism. *NAR Mol. Med.* **2**, ugaf035 (2025).

98. M. Potiri, C. Moschou, Z. Erpapazoglou, G. Rouni, A. Kotsoni, M. Andreadou, M. Dragolia, V. Ntafis, J. Schrader, J. Juan-Mateu, V. Kostourou, S. G. Dedos, M. E. Rogalska, P. Kafasla, A neural alternative splicing program controls cellular function and growth in Pancreatic Neuroendocrine tumours. bioRxiv 2024.06.13.598849 [Preprint] (2024). https://doi.org/10.1101/2024.06.13.598849.

99. A. J. Best, U. Braunschweig, M. Wu, S. Farhangmehr, A. Pasculescu, J. J. Lim, L. C. Comsa, M. Jen, J. Wang, A. Datti, J. L. Wrana, S. P. Cordes, R. Al-Awar, H. Han, B. J. Blencowe, High-throughput sensitive screening of small molecule modulators of microexon alternative splicing using dual Nano and Firefly luciferase reporters. *Nat. Commun.* **15**, 6328 (2024).

100. C. Kosuta, K. Daniel, D. L. Johnstone, K. Mongeon, K. Ban, S. LeBlanc, S. MacLeod, K. Et-Tahiry, M. Ekker, A. MacKenzie, I. Pena, High-throughput DNA extraction and genotyping of 3dpf zebrafish larvae by fin clipping. *J. Vis. Exp.* **136**, 58024 (2018).

101. F. Kroll, J. Donnelly, G. G. Özcan, E. Mackay, J. Rihel, Behavioural pharmacology predicts disrupted signalling pathways and candidate therapeutics from zebrafish mutants of Alzheimer's disease risk genes. *Elife* **13**, RP96839 (2025).

102. C. Pantoja, A. Hoagland, E. C. Carroll, V. Karalis, A. Conner, E. Y. Isacoff, Neuromodulatory regulation of behavioral individuality in zebrafish. *Neuron* **91**, 587–601 (2016).

103. J. M. Wooldridge, *Introductory Econometrics: A Modern Approach* (Cengage Learning, ed. 6, 2016).

104. P. Antinucci, M. Folgueira, I. H. Bianco, Pretectal neurons control hunting behaviour. *Elife* **8**, e48114 (2019).

105. I. H. Bianco, F. Engert, Visuomotor transformations underlying hunting behavior in zebrafish. *Curr. Biol.* **25**, 831–846 (2015).

106. B. Raj, D. E. Wagner, A. McKenna, S. Pandey, A. M. Klein, J. Shendure, J. A. Gagnon, A. F. Schier, Simultaneous single-cell profiling of lineages and cell types in the vertebrate brain. *Nat. Biotechnol.* **36**, 442–450 (2018).

107. B. Raj, J. A. Farrell, J. Liu, J. El Kholtei, A. N. Carte, J. Navajas Acedo, L. Y. Du, A. McKenna, Đ. Relić, J. M. Leslie, A. F. Schier, Emergence of neuronal diversity during vertebrate brain development. *Neuron* **108**, 1058–1074.e6 (2020).

108. H. M. T. Choi, M. Schwarzkopf, M. E. Fornace, A. Acharya, G. Artavanis, J. Stegmaier, A. Cunha, N. A. Pierce, Third-generation in situ hybridization chain reaction: Multiplexed, quantitative, sensitive, versatile, robust. *Development* **145**, dev165753 (2018).

109. H. S. Bruce, G. Jerz, S. Kelly, J. McCarthy, A. Pomerantz, G. Senevirathne, A. Sherrard, D. A. Sun, C. Wolff, N. H. Patel, "Hybridization chain reaction (HCR) in situ protocol" (2021).

110. O. Randlett, C. L. Wee, E. A. Naumann, O. Nnaemeka, D. Schoppik, J. E. Fitzgerald, R. Portugues, A. M. B. Lacoste, C. Riegler, F. Engert, A. F. Schier, Whole-brain activity mapping onto a zebrafish brain atlas. *Nat. Methods* **12**, 1039–1046 (2015).

111. J. J. Woods, J. J. Wilson, Inhibitors of the mitochondrial calcium uniporter for the treatment of disease. *Curr. Opin. Chem. Biol.* **55**, 9–18 (2020).

112. J. D. Buenrostro, P. G. Giresi, L. C. Zaba, H. Y. Chang, W. J. Greenleaf, Transposition of native chromatin for fast and sensitive epigenomic profiling of open chromatin, DNA-binding proteins and nucleosome position. *Nat. Methods* **10**, 1213–1218 (2013).

113. H. Zhuang, H. Wang, Z. Ji, findPC: An R package to automatically select the number of principal components in single-cell analysis. *Bioinformatics* **38**, 2949–2951 (2022).

114. H. C. Park, C. H. Kim, Y. K. Bae, S. Y. Yeo, S. H. Kim, S. K. Hong, J. Shin, K. W. Yoo, M. Hibi, T. Hirano, N. Miki, A. B. Chitnis, T. L. Huh, Analysis of upstream elements in the HuC promoter leads to the establishment of transgenic zebrafish with fluorescent neurons. *Dev. Biol.* **227**, 279–293 (2000).

115. J. A. Lister, C. P. Robertson, T. Lepage, S. L. Johnson, D. W. Raible, nacre encodes a zebrafish microphthalmia-related protein that regulates neural-crest-derived pigment cell fate. *Development* **126**, 3757–3767 (1999).

116. B. V. North, D. Curtis, P. C. Sham, A note on the calculation of empirical P values from Monte Carlo procedures. *Am. J. Hum. Genet.* **71**, 439–441 (2002).

117. B. Phipson, G. K. Smyth, Permutation P-values should never be zero: Calculating exact P-values when permutations are randomly drawn. *Stat. Appl. Genet. Mol. Biol.* **9**, Article39 (2010).

118. H. Fotowat, F. Engert, Neural circuits underlying habituation of visually evoked escape behaviors in larval zebrafish. *Elife* **12**, e82916 (2023).

119. J. Rihel, D. A. Prober, A. Arvanites, K. Lam, S. Zimmerman, S. Jang, S. J. Haggarty, D. Kokel, L. L. Rubin, R. T. Peterson, A. F. Schier, Zebrafish behavioral profiling links drugs to biological targets and rest/wake regulation. *Science* **327**, 348–351 (2010).

120. L. Sheets, D. G. Ransom, E. M. Mellgren, S. L. Johnson, B. J. Schnapp, Zebrafish melanophilin facilitates melanosome dispersion by regulating dynein. *Curr. Biol.* **17**, 1721–1734 (2007).

121. B. B. Avants, N. J. Tustison, G. Song, P. A. Cook, A. Klein, J. C. Gee, A reproducible evaluation of ANTs similarity metric performance in brain image registration. *Neuroimage* **54**, 2033–2044 (2011).

122. T. Kawashima, M. F. Zwart, C.-T. Yang, B. D. Mensh, M. B. Ahrens, The serotonergic system tracks the outcomes of actions to mediate short-term motor learning. *Cell* **167**, 933–946.e20 (2016).

123. G. D. Marquart, K. M. Tabor, M. Brown, J. L. Strykowski, G. K. Varshney, M. C. LaFave, T. Mueller, S. M. Burgess, S.-I. Higashijima, H. A. Burgess, A 3D searchable database of transgenic zebrafish Gal4 and Cre lines for functional neuroanatomy studies. *Front. Neural Circuits* **9**, 78 (2015).

124. G. D. Marquart, K. M. Tabor, E. J. Horstick, M. Brown, A. K. Geoca, N. F. Polys, D. D. Nogare, H. A. Burgess, High-precision registration between zebrafish brain atlases using symmetric diffeomorphic normalization. *Gigascience* **6**, 1–15 (2017).

125. B. Phipson, C. B. Sim, E. R. Porrello, A. W. Hewitt, J. Powell, A. Oshlack, Propeller: Testing for differences in cell type proportions in single cell data. *Bioinformatics* **38**, 4720–4726 (2022).

126. G. C. Linderman, J. Zhao, M. Roulis, P. Bielecki, R. A. Flavell, B. Nadler, Y. Kluger, Zero-preserving imputation of single-cell RNA-seq data. *Nat. Commun.* **13**, 192 (2022).

127. T. Wu, E. Hu, S. Xu, M. Chen, P. Guo, Z. Dai, T. Feng, L. Zhou, W. Tang, L. Zhan, X. Fu, S. Liu, X. Bo, G. Yu, clusterProfiler 4.0: A universal enrichment tool for interpreting omics data. *Innovation (Camb.)* **2**, 100141 (2021).

128. I. Shainer, E. Kuehn, E. Laurell, M. Al Kassar, N. Mokayes, S. Sherman, J. Larsch, M. Kunst, H. Baier, A single-cell resolution gene expression atlas of the larval zebrafish brain. *Sci. Adv.* **9**, eade9909 (2023).

129. T. Stuart, A. Butler, P. Hoffman, C. Hafemeister, E. Papalexi, W. M. Mauck III, Y. Hao, M. Stoeckius, P. Smibert, R. Satija, Comprehensive integration of single-cell data. *Cell* **177**, 1888–1902.e21 (2019).

130. R. Patro, G. Duggal, M. I. Love, R. A. Irizarry, C. Kingsford, Salmon provides fast and bias-aware quantification of transcript expression. *Nat. Methods* **14**, 417–419 (2017).

131. C. Soneson, M. I. Love, M. D. Robinson, Differential analyses for RNA-seq: Transcript-level estimates improve gene-level inferences. *F1000Res.* **4**, 1521 (2015).

132. M. E. Ritchie, B. Phipson, D. Wu, Y. Hu, C. W. Law, W. Shi, G. K. Smyth, limma powers differential expression analyses for RNA-sequencing and microarray studies. *Nucleic Acids Res.* **43**, e47 (2015).

133. M. I. Love, W. Huber, S. Anders, Moderated estimation of fold change and dispersion for RNA-seq data with DESeq2. *Genome Biol.* **15**, 550 (2014).

134. P. Langfelder, S. Horvath, WGCNA: An R package for weighted correlation network analysis. *BMC Bioinformatics* **9**, 559 (2008).

135. A. Franceschini, D. Szklarczyk, S. Frankild, M. Kuhn, M. Simonovic, A. Roth, J. Lin, P. Minguez, P. Bork, C. von Mering, L. J. Jensen, STRING v9.1: Protein-protein interaction networks, with increased coverage and integration. *Nucleic Acids Res.* **41**, D808–D815 (2013).

136. P. W. Harrison, M. R. Amode, O. Austine-Orimoloye, A. G. Azov, M. Barba, I. Barnes, A. Becker, R. Bennett, A. Berry, J. Bhai, S. K. Bhurji, S. Boddu, P. R. Branco Lins, L. Brooks, S. B. Ramaraju, L. I. Campbell, M. C. Martinez, M. Charkhchi, K. Chougule, A. Cockburn, C. Davidson, N. H. De Silva, K. Dodiya, S. Donaldson, B. El Houdaigui, T. E. Naboulsi, R. Fatima, C. G. Giron, T. Genez, D. Grigoriadis, G. S. Ghattaoraya, J. G. Martinez, T. A. Gurbich, M. Hardy, Z. Hollis, T. Hourlier, T. Hunt, M. Kay, V. Kaykala, T. Le, D. Lemos, D. Lodha, D. Marques-Coelho, G. Maslen, G. A. Merino, L. P. Mirabueno, A. Mushtaq, S. N. Hossain, D. N. Ogeh, M. P. Sakthivel, A. Parker, M. Perry, I. Piližota, D. Poppleton, I. Prosovetskaia, S. Raj, J. G. Pérez-Silva, A. I. A. Salam, S. Saraf, N. Saraiva-Agostinho, D. Sheppard, S. Sinha, B. Sipos, V. Sitnik, W. Stark, E. Steed, M.-M. Suner, L. Surapaneni, K. Sutinen, F. F. Tricomi, D. Urbina-Gómez, A. Veidenberg, T. A. Walsh, D. Ware, E. Wass, N. L. Willhoft, J. Allen, J. Alvarez-Jarreta, M. Chakiachvili, B. Flint, S. Giorgetti, L. Haggerty, G. R. Ilsley, J. Keatley, J. E. Loveland, B. Moore, J. M. Mudge, G. Naamati, J. Tate, S. J. Trevanion, A. Winterbottom, A. Frankish, S. E. Hunt, F. Cunningham, S. Dyer, R. D. Finn, F. J. Martin, A. D. Yates, Ensembl 2024. *Nucleic Acids Res.* **52**, D891–D899 (2024).

137. M. Ascensão-Ferreira, R. Martins-Silva, N. Saraiva-Agostinho, N. L. Barbosa-Morais, betAS: Intuitive analysis and visualization of differential alternative splicing using beta distributions. *RNA* **30**, 337–353 (2024).

138. S. B. Thyme, L. M. Pieper, E. H. Li, S. Pandey, Y. Wang, N. S. Morris, C. Sha, J. W. Choi, K. J. Herrera, E. R. Soucy, S. Zimmerman, O. Randlett, J. Greenwood, S. A. McCarroll, A. F. Schier, Phenotypic landscape of schizophrenia-associated genes defines candidates and their shared functions. *Cell* **177**, 478–491.e20 (2019).

139. T. Rohlfing, User guide to the computational morphometry toolkit (The Insight Journal, 2011); https://doi.org/10.54294/ttdjo3.

140. P.-S. Liao, T.-S. Chen, P. Chung, A fast algorithm for multilevel thresholding. *J. Inf. Sci. Eng.* **17**, 713–727 (2001).

141. A. M. Bolger, M. Lohse, B. Usadel, Trimmomatic: A flexible trimmer for Illumina sequence data. *Bioinformatics* **30**, 2114–2120 (2014).

142. H. Li, Aligning sequence reads, clone sequences and assembly contigs with BWA-MEM. arXiv:1303.3997 [q-bio.GN] (2013).

143. H. Li, B. Handsaker, A. Wysoker, T. Fennell, J. Ruan, N. Homer, G. Marth, G. Abecasis, R. Durbin, 1000 Genome Project Data Processing Subgroup, The sequence alignment/map format and SAMtools. *Bioinformatics* **25**, 2078–2079 (2009).

144. F. Ramírez, D. P. Ryan, B. Grüning, V. Bhardwaj, F. Kilpert, A. S. Richter, S. Heyne, F. Dündar, T. Manke, deepTools2: A next generation web server for deep-sequencing data analysis. *Nucleic Acids Res.* **44**, W160–W165 (2016).

145. Y. Zhang, T. Liu, C. A. Meyer, J. Eeckhoute, D. S. Johnson, B. E. Bernstein, C. Nusbaum, R. M. Myers, M. Brown, W. Li, X. S. Liu, Model-based analysis of ChIP-Seq (MACS). *Genome Biol.* **9**, R137 (2008).

146. Y. Liao, G. K. Smyth, W. Shi, featureCounts: An efficient general purpose program for assigning sequence reads to genomic features. *Bioinformatics* **30**, 923–930 (2014).

147. M. Bentsen, P. Goymann, H. Schultheis, K. Klee, A. Petrova, R. Wiegandt, A. Fust, J. Preussner, C. Kuenne, T. Braun, J. Kim, M. Looso, ATAC-seq footprinting unravels kinetics

of transcription factor binding during zygotic genome activation. *Nat. Commun.* **11**, 4267 (2020).
